# MGAS: a powerful tool for multivariate gene-based genome-wide association analysis

**DOI:** 10.1093/bioinformatics/btu783

**Published:** 2014-11-26

**Authors:** Sophie Van der Sluis, Conor V. Dolan, Jiang Li, Youqiang Song, Pak Sham, Danielle Posthuma, Miao-Xin Li

**Affiliations:** ^1^Department of Complex Trait Genetics, Section Clinical Genetics, Center for Neurogenomics and Cognitive Research (CNCR), VU Medical Center, Amsterdam, The Netherlands, ^2^Department of Biological Psychology, VU University Amsterdam, Amsterdam, The Netherlands,^ 3^Department of Biochemistry, ^4^State Key Laboratory for Cognitive and Brain Sciences, ^5^The Centre for Reproduction, Development and Growth, ^6^The Centre for Genomic Sciences and ^7^Department of Psychiatry, The University of Hong Kong, Pokfulam, Hong Kong and ^8^Department of Complex Trait Genetics, Center for Neurogenomics and Cognitive Research (CNCR), VU University Amsterdam, Amsterdam, The Netherlands

## Abstract

**Motivation**: Standard genome-wide association studies, testing the association between one phenotype and a large number of single nucleotide polymorphisms (SNPs), are limited in two ways: (i) traits are often multivariate, and analysis of composite scores entails loss in statistical power and (ii) gene-based analyses may be preferred, e.g. to decrease the multiple testing problem.

**Results**: Here we present a new method, multivariate gene-based association test by extended Simes procedure (MGAS), that allows gene-based testing of multivariate phenotypes in unrelated individuals. Through extensive simulation, we show that under most trait-generating genotype–phenotype models MGAS has superior statistical power to detect associated genes compared with gene-based analyses of univariate phenotypic composite scores (i.e. GATES, multiple regression), and multivariate analysis of variance (MANOVA). Re-analysis of metabolic data revealed 32 False Discovery Rate controlled genome-wide significant genes, and 12 regions harboring multiple genes; of these 44 regions, 30 were not reported in the original analysis.

**Conclusion**: MGAS allows researchers to conduct their multivariate gene-based analyses efficiently, and without the loss of power that is often associated with an incorrectly specified genotype–phenotype models.

**Availability and implementation**: MGAS is freely available in KGG v3.0 (http://statgenpro.psychiatry.hku.hk/limx/kgg/download.php). Access to the metabolic dataset can be requested at dbGaP (https://dbgap.ncbi.nlm.nih.gov/). The R-simulation code is available from http://ctglab.nl/people/sophie_van_der_sluis.

**Contact**: mxli@hku.hk

**Supplementary information**: Supplementary data are available at *Bioinformatics* online.

## 1 Introduction

Standard genome-wide association studies (GWAS) involve the univariate regression of one trait on a large number of genetic variants (single nucleotide polymorphisms, i.e. SNPs), while adapting the nominal α criterion level for the extensive multiple testing (typically α = 5 × 10^−^^8^). This analysis is limited in two important ways. First, genes, not SNPs, are the functional unit in the genome (Huang *et al*., 2011; Li *et al*., 2011). Although SNPs may have different allele frequencies and linkage disequilibrium (LD) structure across human populations, genic function is highly consistent. Furthermore, analysing genes rather than SNPs decreases the multiple testing problem, and relaxing the significance threshold in gene-based analyses improves the statistical power of GWAS studies (Petersen *et al*., 2013).

**Fig. 1.**
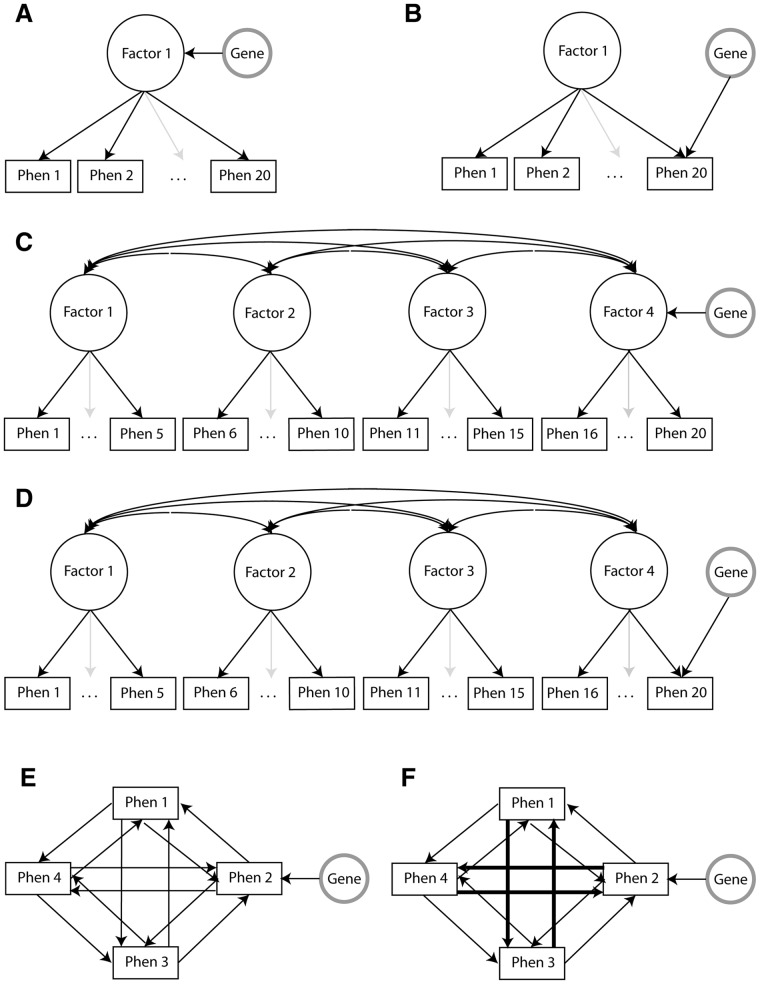
Schematic representation of six trait-generating genotype–phenotype models. (**A**) 1-factor model with all SNPS within a gene affecting the latent factor, and through the latent factor all underlying phenotypes. (**B**) 1-factor model with all SNPs within a gene affecting one underlying phenotype directly. (**C**) 4-factor model with all SNPS within a gene affecting only one of the four latent factors, and all phenotypes underlying that factor. (**D**) 4-factor model with all SNPS within a gene affecting one underlying phenotype directly. (**E**) Network model in which all phenotypes are equally and bidirectionally related, yielding a phenotypic variance–covariance matrix mimicking that of a 1-factor model. All SNPs within a gene affect one phenotype directly and all related phenotypes indirectly. (**F**) Network model distinguishing four clusters of phenotypes; all phenotypes are bidirectionally related, with relations being stronger within, compared with between, clusters, yielding a phenotypic variance–covariance matrix mimicking that of a 4-factor model. All SNPs within a gene affect one phenotype directly and all related phenotypes indirectly. See Supplementary Material for specific simulation settings

Second, traits of interest are often multivariate in nature, i.e. multiple phenotypes are measured to cover the full extent of a trait. For instance, cognitive ability is usually measured through test batteries covering various cognitive abilities (e.g. vocabulary, memory). Similarly, diagnostic interviews and clinical questionnaires cover a collection of symptoms to deal with the symptomatic heterogeneity. In the GWAS context, this multivariate information is generally reduced to a univariate score, i.e. a univariate full-scale IQ score, or a binary case–control index. Reducing multivariate to univariate data nearly always results in loss of information. Specifically, whether univariate composite scores exhaustively summarize all information in the multivariate data (i.e. are sufficient statistics) depends on the true trait-generating genotype–phenotype model, i.e. the model that describes how the multiple phenotypes and genes jointly generate the observed trait (Van der Sluis *et al.*, 2010, 2013). (Specifically, in factor analytic terms, sum scores are only sufficient statistics if (a) all correlations between the phenotypes are explained by one latent trait or factor, (b) all phenotypes have identical factor loadings, (c) all phenotypes have identical residual variances [i.e. the phenotypes adhere to a so-called Rasch (1980) model], and the genetic effect is on the latent factor (illustrated in Fig. 1A). In all other cases, the sum score is not a sufficient statistic and conducting association analysis on the sum score will result in a considerable loss in power to detect genetic effects (Van der Sluis *et al.*, 2010). Noteworthy: the variance–covariance structure of data generated through a network model may closely mimic the variance-covariance structure of data generated through a 1-factor model (Van der Sluis *et al*., 2013; Van der Maas *et al.*, 2006), implicating that factor analytic results are not sufficient to determine the true trait-generating model.) Various simulation studies have shown that overreliance on the unidimensional trait-generating model, and the associated use of univariate composite scores, can result in considerable loss of statistical power to detect genetic variants (Medland and Neale, 2010; Minica *et al*., 2010; Van der Sluis *et al.*, 2010, 2013).

Gene-based methods for the univariate trait setting are available (e.g. GATES, VEGAS, JAG, Li *et al*., 2011; Lips *et al*., 2012; Liu *et al.*, 2010; Ruano *et al*., 2010), as are multivariate methods for the genome-wide SNP-based setting [e.g. MultiPhen, canonical correlation analysis (CCA), i.e. multivariate analysis of variance (MANOVA) with the SNP-effect treated as covariate, TATES and JAMP: Ferreira and Purcell, 2009; O'Reilly *et al*., 2012; Van der Sluis *et al.*, 2013; http://ctglab.nl/software/]. However, to date, only two recently published methods (Basu *et al.*, 2013; Tang and Ferreira, 2012) incorporates both. In fact, the rapid multivariate multiple linear regression (RMMLR) method proposed by Basu *et al.* is essentially equivalent to the multivariate gene-based CCA/MANOVA proposed by Tang and Ferreira, leaving only one MANOVA-based method. MANOVA (and thus RMMLR), however, is known to be powerful specifically if the genetic variant affects only one or a few of multiple correlated phenotypes, but power decreases when the variant affects all or many of the phenotypes (Cole *et al.*, 1994; Medland and Neale, 2010; Minica *et al.*, 2010). Here, we present a multivariate gene-based genome-wide analysis tool that is based on an extended Simes test (Li *et al.*, 2011), MGAS (Multivariate Gene-based Association test by extended Simes procedure). MGAS combines *P*-value information obtained in standard univariate genome-wide SNP-based association software to arrive at a multivariate gene-based *P*-value *P*_MGAS_. The standard GWAS can thus be considered an MGAS preprocessing step. MGAS is implemented in knowledge-based mining system for genome-wide genetic studies (KGG v3.0), is freely available (http://statgenpro.psychiatry.hku.hk/limx/kgg/download.php), and has a user-friendly graphical interface for loading *P*-value files and genetic and phenotypic correlational information, and for visualizing results and annotating sequence variants and interesting genes.

## 2 Methods and results

### 2.1 The MGAS algorithm

Suppose *m* phenotypes and *n* SNPs located in one gene. Regular GWAS software [For data including unrelated individuals: e.g. PLINK, Mach2dat/DSL, SNPtest, Gen/ProbABEL and FaST-LMM (Purcell *et al.*, 2007; Aulchenko *et al*., 2007, 2010; Li *et al*., 2009, 2010; Marchini *et al*., 2007; Lippert *et al.*, 2011).] tests the *m* × *n* univariate phenotype-SNP associations using a statistically appropriate method (e.g. linear or logistic regression depending on the measurement scale of the phenotypes). MGAS subsequently combines the resulting, ascendingly ordered, *P*-values *p*_1 _… *p_m_*_ __×__ _*_n_* to obtain one overall, multivariate gene-based *P*-value *P*_MGAS_ as follows:

(1) $$P_{\text{MGAS}}=\text{min}\left(\frac{q_{e}p_{j}}{q_{ej}}\right)$$

Here, *q_e_* denotes the effective number of independent *P*-values within a gene. Whereas the total number of *P*-values equals *m* × *n*, the effective number is corrected for the fact that the *P*-values are dependent, i.e. correlated due to both the correlations between the phenotypes and the correlations between the SNPs. Parameter *q_ej_* denotes the effective number of *P*-values among the top *j P*-values, where *j* runs from 1 to *m* × *n*, and *p_j_* denotes the *j*th *P*-value in the list of ordered *P*-values. *P*_MGAS_ is thus the smallest weighted *P*-value associated with the null hypothesis that there are no associations between the *m* phenotypes and the *n* SNPs within the gene, and the alternative hypothesis that at least one of the *m* phenotypes is associated to at least one of the *n* SNPs in the gene.

Let *p*_1_ and *p*_2_ denote the *P*-values associated with the test of the association between phenotype *m*_1_ and SNP *n*_1_, and between phenotype *m*_2_ and SNP *n*_2_. The correlation between *p*_1_ and *p*_2_ depends on the observed correlations between the SNPs *r_n_*_1_*_n_*_2_, and the observed correlation between the phenotypes *r_m_*_1_*_m_*_2_. An estimate of the effective number of *P*-values among the top *j P*-values, *q_ej_*, is obtained through eigenvalue decomposition of the correlation matrix Φ among the *m* × *n* ascendingly ordered *P*-values. This correlation matrix is not observed, but can be accurately approximated from the *n* × *n* SNP correlation matrix, Ω, and the *m* × *m* phenotypic correlation matrix, Σ (see Section 2.2 later). Specifically, *q_ej_* is calculated as:

(2) $$q_{ej}=j-\overset{j}{\underset{i=1}{\sum }}I\left(\lambda_{i}\right)\left(\lambda_{i}-1\right)$$

where *j* denotes the number of top *j P*-values, *λ_i_* denotes the *i*th eigenvalue, and *I*(*λ_i_*) is an indicator function, which takes on value 0 if *λ_i_* ≤ 1 and value 1 if *λ_i_* > 1:

(3) $$I\left(\lambda_{i}\right)=\{\begin{array}{c} \;0,\lambda_{i}\;\leq \;1\\ 1,{\;\lambda }_{i}>1\; \end{array}$$

The effective number of *P*-values *q_ej_* is thus calculated as the observed number of *P*-values *j* minus the sum of the difference between *λ_i_* and 1 for those eigenvalues > 1. If all *j P*-values are uncorrelated (i.e. if the phenotypes *m* and/or SNPs *n* involved in the statistical tests giving rise to the *j P*-values, are uncorrelated), then all *j* eigenvalues equal 1, and *q_ej_* = *j* − 0 = *j*. Conversely, if the *j P*-values are perfectly correlated (i.e. if the phenotypes *m* and the SNPs *n* are perfectly correlated), then the first eigenvalue equals *j*, all other eigenvalues equal 0, and *q_ej_* = *j* − (*j* − 1) = 1 (i.e. testing the association of perfectly correlated phenotypes with perfectly correlated SNPs yields only one unique unit of information). In practice, we expect that *q_ej_* will generally be smaller than *j* but larger than 1, since the phenotypic correlations and the SNP correlations likely take on values between 0 and |1|. Finally, *q_e_* is a special case of *q_ej_*, i.e. *q_e_* = *q_ej_* when *j* = *m* × *n*, i.e. when the selection of *P*-values *j* covers all *m* × *n P*-values.

It is important to note that as *q_e_* is by definition ≥*q_ej_*, the weighted *p_j_* is always ≥ the unweighted *p_j_*. This weighting procedure has three consequences. First, when all *P*-values are large (i.e. non-significant tests), the originally largest *P*-value can sometimes be selected by the MGAS procedure as *P*_MGAS_, i.e. as the smallest weighted *P*-value that is selected as the multivariate gene-based *P*-value. Because for the largest *P*-value the weight *q_e_*/*q_ej_* = 1, and *q_e_*/*q_ej_* > 1 for all other *P*-values, the largest *P*-value before weighting can become the smallest *P*-value after weighting when all *P*-values were high to begin with. Second, because for most *P*-values *q_e_*/*q_ej_* > 1, weighted *P*-values can become >1, especially when the unweighted *P*-values are large to begin with. However, *P*_MGAS_ cannot exceed 1. Third, the search for the smallest weighted *P*-value can stop once *q_e_p_j_*/*q_ej_* is ≤ *p_j_*_ __+__ __1_: as the weight *q_e_*/*q_ej_* is always ≥1, no subsequent weighted *P*-values will be ≤ *q_e_p*_j_/*q_ej_*. This rule saves computation time for calculating the effective number of independent tests.

### 2.2 Approximation of P-value correlations

The *m* × *n* correlation matrix between the *P*-values is not observed in practice. Following Li *et al.*, 2011 we used simulation to derive the mathematical relationship between the observed SNP-correlations Ω and observed phenotypic correlation Σ on the one hand, and the correlations between the *P*-values Φ on the other, under the assumption of no genetic association (i.e. under the null hypothesis). Assuming two correlated bi-allelic SNPs and two correlated phenotypes, genotype and phenotype data were simulated for *N* = 4000 subjects for every combination of allele frequencies (assuming Hardy–Weinberg equilibrium), LD coefficient *r*, and phenotypic correlation ρ. For each LD coefficient *r*, the SNP haplotypes were randomly generated by reference to a categorical distribution. For quantitative phenotypes, phenotypic scores were randomly drawn from a bivariate normal distribution $N∼\left(\left[\begin{array}{c} 0\\ 0 \end{array}\right],\left[\begin{array}{cc} 1 & \text{ρ}\\ \text{ρ} & 1 \end{array}\right]\right)$, and a linear regression model (Wald test) was used to evaluate the statistical association between each phenotype and each SNP. For qualitative phenotypes, the bivariate Bernouilli distribution with correlation ρ was used to produce pairs of dichotomous phenotypic scores for each subject, and a logistic regression model (χ^2^ test) was used to evaluate the association between each phenotype and each SNP. Allele frequencies, *r*, and ρ were increased from the smallest values to the largest values with steps of 0.05 to generate a series of data points. For each setting, 10 000 datasets were generated, yielding 10 000 sets of *P*-values from which the correlation between *P*-values could be estimated. Our simulations then showed that the correlation matrix of the *m* × *n* *P*-values Φ could be accurately approximated using a sixth-order polynomial function featuring the Kronecker multiplication of the observed SNP-correlation matrix Ω and observed phenotypic correlation matrix Σ, with coefficient of determination *R*^2 ^= 0.995:

(4) $$\text{Φ}=f\left(\Sigma ⊗\Omega =X\right)\approx 0.3867X^{6}+0.0021X^{5}-0.1347X^{4}-\;0.0104X^{3}+0.7276X^{2}+0.0068X$$

Neither sample size nor allele frequencies affected the accuracy of this approximation (see Supplementary Fig. S1).

MGAS as implemented in KGG v3.0 calculates the expected matrix Φ using the user-provided correlation matrices Σ and Ω. The first can be calculated from the raw phenotype data, while the latter can either be calculated from the actual genotype data or can be obtained from reference genotype data in HapMap (http://hapmap.ncbi.nlm.nih.gov) or the 1000 Genomes Project (http://www.1000genomes.org/). KGG v3.0 facilitates the building of Ω from both observed and reference data.

### 2.3 MGAS running time: the divide-and-conquer algorithm

To speed up computation of *P*_MGAS_ for a gene with a large number of SNPs and many phenotypes, we developed a divide-and-conquer algorithm. The *m* × *n* weighted *P*-values are partitioned into *K* clusters according to their expected correlations using a cutoff of *c* = 0.50. The *P*-value of each block is calculated by the extended Simes test (Li *et al*., 2011), and the SNP determining the blockwise *P*-value is marked as the key SNP of that block. The block-wise *P*-values are then combined by the same extended Simes test again to produce the multivariate gene-based *P*-value *P*_MGAS_ in which the correlations between the key SNPs are used to correct for the dependency of the *K* block-wise *P*-values. This algorithm avoids computationally intensive algebraic operation in large correlation matrices [e.g. with 20 phenotypes and a gene covering 200 SNPs, the correlation matrix in the analysis has dimensions (20 × 200) × (20 × 200)]. Depending on the number of phenotypes and SNPs per gene, building the genomic correlation matrix and running the MGAS analysis takes 15 min (9 phenotypes and 328 836 SNPs, see empirical example later) to 2 hr (20 phenotypes and 2.4 million SNPs) on an ordinary desktop computer with Intel(R) Core(TM) i7-3770, CPU 3.40 GhZ, RAM 8.00 GB and 64-bit Windows 7.0 Enterprise 2009.

### 2.4 Type I error rate and power: simulation

#### 2.4.1 Simulation settings

To examine the Type I error rate and the power of MGAS to detect associated genes, multivariate genotype–phenotype data were simulated for 2000 unrelated subjects (see Supplementary Information for details). Phenotype data included 20 standard normally distributed phenotypes. The covariance structure of the phenotypic data was either a unidimensional common factor model (Fig. 1A and B), a 4-common factor model (Fig. 1C and D) or a network model in which correlations between phenotypes are the result of direct, mutual relations between the individual phenotypes (Fig. 1E and F; the relevance of network models for psychological traits like cognition, depression and personality has been demonstrated; Borsboom *et al.*, 2011; Cramer *et al*., 2010, 2012; Van der Maas *et al.*, 2006). Network data were generated such that the associated phenotypic covariance structure either mimicked a unidimensional factor model (i.e. all phenotypes mutually affect each other to a similar extent; Fig. 1E), or a 4-factor model (i.e. four clusters of phenotypes are specified; Fig. 1F). Within and between factors or clusters, phenotypes correlated 0.56 and 0.13, respectively. In these six phenotypic settings, the effects of all SNPs within a gene were either modeled at the latent level (Fig. 1A and C), or directly on one specific phenotype (Fig. 1B–F).

Genetic data included a small gene (10 SNPs) or a larger gene (60 SNPs, assuming equal coverage) with varying numbers of disease-susceptibility loci (DSL, i.e. a SNP that is causally related to one or more phenotypes), and LD blocks. Table 1 shows an overview of all simulation settings (see also Supplementary Tables S1a–e for a visual representation of the LD structure and a detailed description of all simulation settings).

**Fig. 2.**
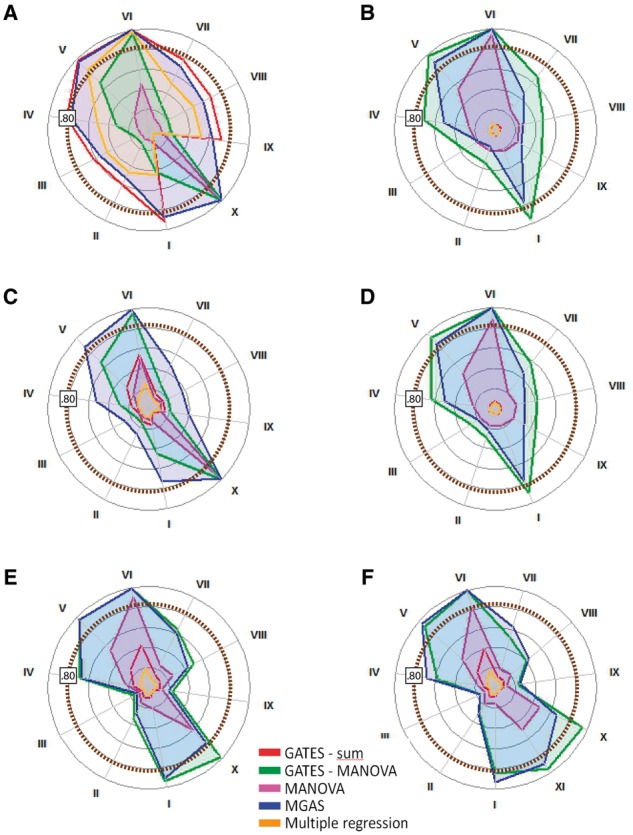
Radial power plots for six trait-generating genotype–phenotype models (A–F) and various genetic situations (I–XI). (**A**) 1-factor model with gene effects on the latent factor. (**B**) 1-factor model with gene affecting only one phenotype directly. (**C**) 4-factor model with gene effect on only one of four latent factors. (**D**) 4-factor model with gene affecting only one phenotype directly. (**E**) Network model mimicking 1-factor model with gene affecting one phenotype directly and all related phenotypes indirectly. (**F**) Network model mimicking 4-factor model with gene affecting one phenotype directly and all related phenotypes indirectly. I–III represent results for the large gene (60 SNPs): (**I**): eight LD blocks, one DSL; (**II**): eight LD blocks, eight DSL; (**III**): eight LD blocks, eight DSL of opposite effect. IV–XI represent result for a small gene (10 SNPs): (**IV**): one LD block, one DSL; (**V**): one LD block, two DSL; (**VI**): one LD block, four DSL; (**VII**): two LD blocks, one DSL; (**VIII**): two LD blocks, two DSL; (**IX**): two LD block, two DSL of opposite effect; (**X**): one LD block, one DSL conveying opposite effects on different phenotypes (that in network models resided in the same cluster); (**XI**): one LD block, one DSL conveying opposite effects on phenotypes in different clusters. Specific power results and Type I error rates for all scenarios are in Supplementary Tables S2–S7. Because MGAS has less power to detect larger genes (see Supplementary Material), small genes and large genes were simulated to explain 0.5 and 1% of the variance, respectively. Power results of the five methods are thus not directly comparable between scenarios (see Supplementary Material)

**Table 1.** Overview simulation settings

		Number of LD blocks	Number of DSL	Opposite effects
Small gene	Scenario 1	1	0	
(10 SNPs)	Scenario 2 (IV)	1	1	No
	Scenario 3 (X, XI)	1	1	Yes^a^
	Scenario 4 (V)	1	2	No
	Scenario 5	1	2	Yes^b^
	Scenario 6 (VI)	1	4	No
	Scenario 7	1	4	Yes^b^
	Scenario 8 (VII)	2	1	No
	Scenario 9 (VIII)	2	2	No
	Scenario 10 (IX)	2	2	Yes^b^
Large gene	Scenario 11	8	0	
(60 SNPs)	Scenario 12 (I)	8	1	No
	Scenario 13 (II)	8	8	No
	Scenario 14 (III)	8	8	Yes^b^

Note. DSL: disease susceptibility locus. Roman numerals refer to the power results in Figure 2A.

^a^In latent factor models (Fig. 1A and C), the one DSL affected half of the phenotypic indicators of a factor positively and the other half negatively. In unclustered networks (Fig. 1E), the one DSL affected one phenotype positively and another phenotype in the network negatively. In clustered networks (Fig. 1F), the one DSL affected either two phenotypes in the same cluster, or two phenotypes in different clusters, in opposite directions.

^b^Half of the DSL in a gene conveyed a positive effect, the others a negative effect of the same magnitude (Fig. 1A and C) or phenotypes (Fig. 1C–F).

In all simulations, the minor allele frequency of all SNPs was 0.2. Within and between LD blocks, LD measure *r* was set to 0.9 and 0, respectively. The simulated regression weights of DSL were chosen to account for 0.5% of the phenotypic variance in small genes and 1% in large genes (see Section 2.5 later). Note that in the univariate regression of one phenotype on a DSL, the effect of the DSL can be larger than simulated when the DSL is in high LD with other DSL (i.e. the effect is augmented because of the correlation of the DSL to other DSL in the same LD block: see Supplementary Information Simulations). Hence, power comparisons should only be made within each scenario, not between scenarios.

In each scenario, data were analysed in five ways, all yielding a gene-based *P*-value. First, the sum score, calculated across all 20 phenotypes, was regressed on all SNPs within a gene, and the *P*-values from these univariate tests were submitted to the GATES-sum procedure. Second, this sum score was submitted to a multiple regression model, in which all SNPs within a gene were included as predictors. Third, MANOVA was conducted with all 20 phenotypes as dependent variables, and all SNPs within a gene as predictors (Tang and Ferreira, 2012). In both multiple regression and MANOVA, fixing all predictor effects to zero simultaneously yields a gene-based test with the number of degrees of freedom equal to the number of SNPs in a gene. Note that for these simulation settings (i.e. unrelated individuals, no covariates and an additive codominant SNP), MANOVA is equivalent to CCA (Tang and Ferreira, 2012) and the RMMLR method (Basu *et al.*, 2013). Fourth, as various studies (e.g. Cole *et al*., 1994; Medland and Neale, 2010; Minica *et al*., 2010; Galesloot *et al.*, 2014) have shown that MANOVA featuring one predictor (i.e. SNP) has excellent power to pick up trait-specific effects (i.e. only one of multiple correlated phenotypes is affected), and SNPs with opposite effects, we performed a combination of MANOVA and GATES (i.e. GATES-MANOVA). That is: given *m* phenotypes and *n* SNPs, *n* multivariate SNP-based MANOVAs with all *m* phenotypes as dependent variables and each SNP separately as predictor, produced *n P*-values that were combined to one multivariate gene-based *P*-value using GATES. Finally, all *m* phenotypes were individually regressed on all *n* SNPs, and the *m* × *n P*-values from these univariate tests were submitted to the MGAS procedure. Importantly, the first two methods summarize the multivariate phenotypic information into one composite score, while the latter three methods preserve the multivariate nature of the phenotypic input.

For all six trait-generating genotype–phenotype models, we simulated and analysed 2000 datasets using the freely available program R (R Development Core Team, 2011), and we counted the number of times that gene-based *P*-values indicated significance given α = 0.05.

#### 

#### 2.4.2 Simulation results

Type I error rates were correct for all methods in all scenarios (Supplementary Tables S2–S7 for precise power and Type I error estimates), bar a small tendency toward inflation (∼0.063) for MGAS over multiple simulation runs (not shown) when analysis concerned a large gene in the clustered network scenario (Fig. 1F), suggesting that MGAS can be slightly liberal in this scenario. To study the Type I error rate for smaller α levels, we ran the scenario illustrated in Figure 1A 100 000 times for a small gene and obtained values of 0.01017, 0.00134 and 0.00011 for α = 0.01, 0.001 and 0.0001, respectively, suggesting correct Type I error rates for more stringent α-levels. In addition, we used real genome-wide genetic data of *N* = 4763 subjects from the Northern Finland Birth Cohort (NFBC1966, Sabatti *et al.*, 2009, see Section 2.6 later), simulated 10 sets of 9 phenotypes, of which the correlational structure mimicked the observed correlations of the NFBC1966 metabolic phenotypes (see Supplementary Table S8), and performed 10 whole-genome multivariate gene-based association analyses using MGAS. These analyses showed that both small (≤10 SNPs) and larger (>10 SNPs) genes had empirical Type I error rates very close to the nominal alpha bar some stochastic fluctuation (Table 2), suggesting that MGAS has correct Type I error rate regardless of LD pattern and gene size. Indeed, *qq*-plots of the thus obtained MGAS *P*-values against a uniform distribution showed no aberrations (see Supplementary Fig S2).

**Table 2.** Empirical Type I error rates in 10 genome scans using MGAS

			Nominal α	
		0.01	0.001	0.0001
Small genes	≤10	0.0107	0.00116	0.000112
Larger genes	>10	0.0128	0.00151	0.000108

Note. Type I error rates of MGAS for small genes (≤10 SNPs) and larger genes (>10 SNPs) obtained in 10 whole-genome scans of real genomic data and nine simulated phenotypes with realistic correlational structure.

**Table 3.** Top genes/regions identified by MGAS

Gene	*P*_MGAS_	Chrom	SNP	GeneFeature	BMI	CRP	DBP	GLU	HDL	INS	LDL	SBP	TG
CETP	2.64E − 27	16	rs5882	Exonic	0.0334	0.2277	0.8921	0.7482	0.0123	0.6682	0.9856	0.7947	0.2581
CETP			rs7499892	Intronic	0.0533	0.3716	0.2021	0.0581	2.29E−16	0.6125	0.8722	0.7906	0.6591
CETP			rs1532624	Intronic	0.1196	0.3991	0.7190	0.1250	2.97E−22	0.0855	0.2530	0.8547	0.0190
CETP			rs3764261	Upstream	0.7867	0.2128	0.5447	0.2744	6.97E−29	0.5155	0.1550	0.9677	0.0776
CETP			rs4784744	Intronic	0.9047	0.2958	0.3813	0.6869	0.0015	0.0104	0.0309	0.6400	0.0094
CRP	4.79E−21	1	rs2794520	Downstream	0.5436	2.92E−22	0.5156	0.5865	0.2572	0.3577	0.5440	0.2896	0.6739
CRP			rs2808630	Downstream	0.7177	0.1592	0.6079	0.8536	0.4855	0.5051	0.6753	0.7260	0.5424
PSRC1	3.66E−11	1	rs646776	Downstream	0.1401	0.2212	0.6273	0.8053	0.1416	0.0778	2.19E−12	0.5509	0.5495
PSRC1			rs14000	3UTR	0.1916	0.09687	0.0892	0.2581	0.6445	0.1435	0.1921	0.5668	0.6507
CELSR2	1.07E−10	1	rs646776	Downstream	0.1401	0.2212	0.6273	0.8053	0.1416	0.0778	2.19E−12	0.5509	0.5495
CELSR2			rs14000	3UTR	0.1916	0.0969	0.0892	0.2581	0.6445	0.1435	0.1921	0.5668	0.6507
CELSR2			rs4970833	Intronic	0.4169	0.4745	0.903	0.1187	0.0869	0.9570	0.0002	0.1492	0.0062
CELSR2			rs585362	Upstream	0.4312	0.1302	0.9314	0.6189	0.1309	0.5631	0.0008	0.9039	0.2540
CELSR2			rs608196	Intronic	0.5951	0.0257	0.2761	0.2334	0.6895	0.5521	0.2932	0.8240	0.7671
CELSR2			rs611917	Intronic	0.6200	0.9362	0.6067	0.2556	0.3181	0.4759	2.49E−07	0.7242	0.4669
CELSR2			rs437444	Exonic	0.9746	0.0124	0.2472	0.0730	0.0753	0.5549	0.2433	0.0343	0.6468
GCKR	9.72E−09	2	rs780090	Upstream	0.4083	0.6039	0.077	0.7938	0.9573	0.9804	0.0014	0.9441	0.0012
GCKR			rs1260326	Exonic	0.4920	0.0731	0.5136	0.3899	0.1862	0.4225	0.3074	0.3802	3.56E−10
GCKR			rs780092	Intronic	0.7381	0.8739	0.9692	0.4828	0.2053	0.8628	0.6050	0.4487	0.0028
GCKR			rs780094	Intronic	0.8774	0.1497	0.3194	0.2858	0.3188	0.2942	0.4940	0.1694	0.2486
G6PC2	1.38E−08	2	rs3821117	Intronic	0.2223	0.4850	0.7182	0.6035	0.4264	0.2869	0.7342	0.1685	0.9942
G6PC2			rs560887	Intronic	0.6882	0.4081	0.9984	5.69E−10	0.7242	0.9469	0.5264	0.9864	0.5293
G6PC2			rs491443	Downstream	0.7258	0.3268	0.4481	0.03655	0.7391	0.9486	0.9368	0.4742	0.6680
HNF1A-AS1	2.48E−08	12	rs2254779	ncRNA	0.5124	0.1338	0.2373	0.9861	0.7389	0.9154	0.6181	0.2521	0.9738
HNF1A-AS1			rs7953249	Downstream	0.5548	1.44E−09	0.4425	0.9052	0.4757	0.5462	0.2955	0.6843	0.9386
HNF1A	5.55E−08	12	rs1169302	Intronic	0.3462	1.20E−07	0.8893	0.0273	0.1185	0.4196	0.3375	0.1404	0.7278
HNF1A			rs2464196	Exonic	0.5168	4.78E−09	0.6792	0.8519	0.7413	0.2968	0.3052	0.6581	0.7054
HNF1A			rs1169300	Intronic	0.6507	2.01E−09	0.6845	0.8948	0.8545	0.3737	0.1907	0.7302	0.6947
HNF1A			rs735396	Intronic	0.7766	2.13E−07	0.9390	0.9274	0.9260	0.1533	0.1433	0.9335	0.2894
HNF1A			rs1169307	Intronic	0.8254	2.76E−05	0.7082	0.7806	0.0823	0.7835	0.3745	0.813	0.9292

Note. Top genes/regions identified by MGAS (multivariate gene-based *P*-value *P*_MGAS_ < 1.0E−07: see Supplementary Table S9 for the entire list of False Discovery Rate controlled significant genes). Abbreviations metabolic phenotypes: body mass index (BMI), C-reactive protein (CRP), diastolic blood pressure (DBP), glucose (GLU), high-density Lipoprotein (HDL), insulin (INS), low-density lipoprotein (LDL), systolic blood pressure (SBP) and triglycerides (TG).

Power results and Type I error rates for all scenarios are in Supplementary Tables S2–S7. Figure 2 depicts power results for the six trait-generating genotype–phenotype models, except for the scenarios including multiple opposite-effect DSL from the same LD block (Scenarios 5 and 7 in Table 1) since none of the five methods had reasonable power (max 9%) to detect these.

As expected (Medland and Neale, 2010; Minica *et al*., 2010; Van der Sluis *et al*., 2010, 2013), the methods that use phenotypic sum scores rather than the multivariate information (i.e. GATES and multiple regression, represented in red and orange, respectively) perform well when the true trait-generating model is a unidimensional factor model with the gene-effect on the latent factor, because only in that specific case the phenotypic sum score is a sufficient statistic (Figs 1A and 2A). That the phenotypic sum score is not a sufficient statistic under the other five simulated genotype–phenotype models is evident from the power results of GATES and multiple regression: the power to detect DSL-harboring genes hardly ever exceeds 20%, and is often close to 5% as expected under the null hypothesis of no genetic signal.

From previous studies (Cole *et al.*, 1994; Galesloot *et al.*, 2014; Medland and Neale, 2010; Minica *et al.*, 2010) MANOVA is known to be particularly powerful when one predictor (e.g. SNP) affects only one of multiple highly correlated phenotypes (Fig. 2B and D), or when that one predictor affects correlated phenotypes in opposite directions (scenario *X* in Fig. 2). Indeed, GATES-MANOVA has the best power in these scenarios, with MGAS as second best (MGAS has 0–22% less power, with a median of −12%). When the gene affects multiple phenotypes simultaneously (i.e. pleiotropy, Fig. 2A and C), MGAS outperforms GATES-MANOVA (−1 to 45% more power, median 22%), but when the genotype–phenotype data have a network structure, MGAS and GATES-MANOVA perform similarly, except, as expected, when one DSL affects highly correlated phenotypes oppositely (power differences range between −28 and 10%, with a median of 0).

In general, MGAS outperforms all other gene-based methods, except when the trait-generating genotype–phenotype model is a unidimensional factor model with the gene-effect on the factor (Fig. 2A), in which case it is sometimes slightly outperformed by the GATES procedure (MGAS has −9% less, to 95% more, power, with a median of 6%). MGAS outperforms MANOVA and multiple regression considerably in this scenario (compared with multiple regression: 0–95%, median 12%; compared with MANOVA: 0–76%, median 52%), and for all other trait-generating genotype–phenotype models, MGAS has (much) more power than GATES-sum, MANOVA and multiple regression (calculated across all other five genotype–phenotype scenarios; compared with GATES-sum: 5–94%, median 45%; compared with multiple regression: 7–95%, median 45%; compared with MANOVA: −0.04 to 77%, median 22%). Although MANOVA including all (highly correlated) SNPs as predictors often outperforms GATES and multiple regression, it is always less powerful than MGAS.

Overall, all methods have less power to detect genes harboring multiple small-effect DSL, unless these are in high LD, in which case MGAS and GATES-MANOVA show sufficient power.

Yet, none of the currently included methods can pick up genes with small-effect DSL that are in high LD but show opposite effects (Scenarios 5 and 7 in Table 1).

### 2.5 Particulars MGAS

Since MGAS revolves about selection of the best SNP (i.e. the SNP with the smallest weighted *P*-value), it is not directly sensitive to increased numbers of DSL within one gene. We note, however, that ‘indirectly’, MGAS does favor genes with multiple DSL because the weights *q_e_*/*q_ej_* [see Equations (1) and (2)] generally decrease quickly. For instance, assuming 10 phenotypes that correlate 0.53 and a gene harboring 10 SNPS that correlate 0.3, the first four weights equal 88.44, 44.94, 31.04 and 23.70. Assuming α = 0.01, to implicate a gene with one DSL, that DSL must thus have a *P*-value < 0.01/88.44 = 0.00011, while to implicate a gene harboring four DSL, the *P*-value of the DSL with the weakest signal only needs to be <0.01/23.70 = 0.0004, i.e. can be approximately four times higher.

At the same time, because the weights *q_e_*/*q_ej_* are based on eigenvalue decomposition, a gene represented by multiple DSL in high LD can be easier to detect than a gene represented by multiple DSL (same signal) in low LD. For instance, while the first four weights *q_e_*/*q_ej_* are 35.66, 19.68, 13.59 and 10.38 when 10 phenotypes correlate 0.53 and 10 SNPs correlate 0.9, they equal 88.44, 44.94, 31.04 and 23.70 when the 10 SNPs correlate only 0.3. Assuming the same number of SNPs representing a gene and DSL with the same effect size, MGAS thus more easily detects genes with DSL that are part of an LD block. This is also illustrated by the relatively high power of MGAS in scenarios V and VI, compared with IV, in Figure 2A–F. However, adding highly correlated, non-causal SNPs to represent a gene more comprehensively quickly decreases the power of MGAS to detect the gene: because *j* in Equation (2) increases when one adds SNPs, the weights *q_e_*/*q_ej_* increase, thereby decreasing the probability that weighted *P*-values will be below a chosen criterion level α. For instance, assuming 10 phenotypes correlating 0.53 and a gene with 60 SNPs divided over six LD blocks (10 SNPs per block, LD = 0.9 within, and LD = 0 between blocks), the first four weights equal 213.94, 115.61, 79.20 and 60.24. If one was to select one SNP per block such that the gene is represented by six unrelated SNPs, the weights would drop to 49.85, 27.51, 19.00 and 14.51, respectively. That is: when adding highly correlated SNPs to the analysis, power only increases if these SNPs are actually DSL that convey an independent association signal. In practice, however, we do not know which SNPs are such DSL. Therefore, while imputation and sequencing yield dense genotype files, the power of MGAS (as of other SNP-based and gene-based tests) to detect associated genes will generally improve if one uses LD-based pruned data in the analysis since most SNPs are not DSL [a similar conclusion was reached for univariate gene-based tests by Petersen *et al*. (2013), who showed that inclusion of non-causal intra-genic variants often dilutes the signal of gene-based tests]. However, as always, the process of LD-based pruning implies a trade-off between gaining power by reducing the number of redundant tests, and losing power by potential pruning of real genetic signal. Whether *P*-value based pruning is suited for procedures like MGAS that are based on *P*-value selection, or maybe introduces bias through capitalization on chance, requires further study.

### 

### 2.6 Implementation: metabolism data

To illustrate the usefulness of MGAS, we re-analysed nine quantitative metabolic traits measured in the population-based NFBC1966 (*N* = 4763; Sabatti *et al.*, 2009), obtained from the dbGaP database. Data were available for triglycerides (TG), high-density lipoprotein (HDL), low-density lipoprotein (LDL), glucose (GLU), insulin (INS), C-reactive protein (CRP), body mass index (BMI) and systolic and diastolic blood pressure (SBP, DBP), and 328 836 SNPs, whose genomic positions were retrieved using SnpTracker (http://statgenpro.psychiatry.hku.hk/limx/snptracker). To run MGAS, KGG v3.0 requires a phenotypic correlation matrix and a file containing the *P*-values from the statistical association test between each SNP and each of the nine phenotypes. Phenotypic correlations, corrected for the covariates used in the original genome-wide analysis, were calculated from the original phenotype data downloaded from dbGaP (Supplementary Table S8). To follow the original analyses and results as closely as possible, we used the original *P*-value files provided by the authors and available in dbGaP (i.e. the *P*-values from the original 9, PCs and covariates corrected, genome-wide analyses). Combining these nine *P*-value files, we had information on 328 836 SNPs, 180 520 of which were located within genes, covering 21 153 genes in total. In KGG v3.0, SNPs were mapped onto genes defined by the RefGene database with 5 kb boundary extensions on both sides. When an SNP was located in overlapping regions of multiple genes, the SNP was assigned to all involved genes. The LD structure within each gene was determined in KGG v3.0 by reference to the HapMap LD data (http://hapmap.ncbi.nlm.nih.gov/downloads/ld_data/latest/).

To illustrate the added value of a test that is both multivariate and gene-based, we also conducted multivariate SNP-based analysis using TATES (Van der Sluis *et al*., 2013) and univariate gene-based analysis using GATES (Li *et al*., 2011) for comparison. TATES was conducted on the original *P*-values and thus concerned multivariate analysis of the nine continuous metabolic trait measures (genome-wide significant Bonferroni corrected α = 1.52 × 10^−^^07^). The NFBC1966 data do not contain univariate metabolic syndrome case/control status scores. Therefore, to conduct the univariate gene-based method GATES, we created a univariate composite score reflecting the number of endorsed metabolic risk factors using five standard clinical diagnostic criteria to identify metabolic syndrome (see http://www.ncbi.nlm.nih.gov/pmc/articles/PMC1880831/). These criteria are: TG ≥ 1.70; HDL males < 1.04, HDL females HDL < 1.30; GLU ≥ 6.1, BMI ≥ 25 and blood pressure ≥ 130/85. For each participant, we established whether they met the criteria (yes/no, coded as 1/0) and then calculated the sum of endorsed criteria. This sum, ranging from 0 to 5 (42.9, 32.9, 15.7, 6.7, 1.6 and 0.1 % of the participants met 0, 1, 2, 3, 4 or 5 of the criteria, respectively), was then treated as a continuous dependent variable in a genome-wide analysis that we conducted in PLINK (Purcell *et al.*, 2007), including, like in the original analyses, sex, use of oral contraception, pregnancy status and the first two PCs of a multidimensional scaling analysis as covariates (the first two PCs were used as covariates by the original authors as a proxy for geographical origin of the subjects). GATES analyses were also performed on the individual risk factors, i.e. the dichotomized variables TG, HDL, GLU, BMI and blood pressure, and on the nine continuous metabolic traits separately.

Using an original Benjamini False Discovery Rate (Benjamini and Hochberg, 1995) controlled genome-wide threshold of α = 1.80 × 10^−^^04^, MGAS identified 32 genome-wide significant genes and 12 genome-wide significant regions harboring multiple genes (Supplementary Table S9; *qq*-plots in Supplementary Figs S3 and S4). Of these 44 regions, 30 were not reported in the original analysis, but 39 have been identified before in unrelated studies on the separate metabolic traits according to the Catalog of Published Genome-wide Association Studies (http://www.genome.gov/gwastudies; Supplementary Table S10). Inspection of the univariate SNP-based *P*-values underlying the multivariate MGAS *P*-values showed that while the top regions (*P* < 1.0 × 10^−^^07^; Table 3) were only implicated in one of the nine metabolic traits, other regions showed associations of varying strength to multiple metabolic traits (e.g. APOB: *P*_MGAS_ = 1.13 × 10^−^^06^, associated to HDL, LDL and TG; TOMM40/PVRL2: *P*_MGAS_ = 1.47 × 10^−^^06^, associated to LDL, TG and CRP). Three of the five regions newly identified by MGAS were previously associated to disorders of which treatment/prognosis is known to be related to metabolism (see Supplementary Table S10). For instance, the region harboring GLT8D1, GNL3, SNORD19B and SNORD69 (here associated to GLU and LDL) has been associated to major psychiatric mood disorders (Goes *et al.*, 2012; McMahon *et al.*, 2010; Sklar *et al.*, 2011), and lipid and glucose abnormalities are reported in a considerable proportion of patients using atypical or second-generation antipsychotics (Pramyothin and Khaodhiar, 2010).

MGAS identified more genes compared with the multivariate SNP-based method TATES (20 genome-wide significant SNPs with *P* < 1.52 × 10^−^^07^ covering 10 genetic regions: Supplementary Table S11) and the univariate gene-based method GATES conducted on the sum of endorsed risk factors (Supplementary Table S12). GATES analyses based on the nine individual continuous metabolic phenotypes (Supplementary Table S13), however, yielded results largely similar to those obtained with MGAS (comparison in Supplementary Table S14), except that the MGAS *P*-values are properly corrected for the phenotypic correlations and multiple testing.

## 3 Discussion

We have shown that the new multivariate gene-based method MGAS has correct Type I error rate and performs well under a variety of trait-generating genotype–phenotype models. With respect to power, MGAS often outperforms other gene-based methods that do not require permutation, like the univariate methods GATES, multiple regression and the multivariate MANOVA [which, with our simulation settings, is equivalent to CCA (Tang and Ferreira, 2012) and RMMLR (Basu *et al*., 2013)].

MGAS, based on *P*-value information obtained in standard software like PLINK, has several important advantages. First, simultaneous analysis of phenotypes that have different measurement scales (e.g. continuous, ordered categorical, dichotomous) is unproblematic. Second, standard GWAS software deals with quality control in a genome-wide setting, covariates and population structure or stratification. MGAS, subsequently using the *P*-values resulting from these specialized packages, benefits from the strengths of such software. Third, MGAS is relatively fast as the method does not require permutation. Fourth, for the simulated genotype–phenotype models, MGAS often proved the most powerful gene-based methods, especially for the detection of pleiotropic genes (Fig. 1A and C). Only when the gene affects that part of one specific phenotype that shows no relation to other phenotypes in the analysis (i.e. gene-effect on the residual, Fig. 1B and D) is MGAS outperformed by GATES-MANOVA. As a powerful method, MGAS more easily detects genes that show relatively weak associations with one or multiple traits. This was nicely illustrated by our real data example, in which we identified 30 regions that were not reported in the original analysis.

It is important to note that, like all other multivariate gene-based methods included in our simulations, MGAS has difficulty detecting genes that harbor multiple DSL in high LD, with contrasting effects (Scenarios 5 and 7 in Supplementary Tables S2–S7). Although contrasting effects of SNPs in high LD, located within one gene, may indeed exist, e.g. as a balancing mechanism, the scope of this scenario, and thus of this shortcoming, is probably limited. Yet, multivariate gene-based methods that can detect such contrasting effects are currently lacking.

We close with some final remarks concerning the practical use of MGAS. First, MGAS, like TATES, cannot be used to analyse data collected in multiple, non-overlapping samples (e.g. trait *A* was measured in sample I, and trait *B* in sample II) because the resulting *P*-values will not show the expected correlations resulting from both the phenotypic correlations and the LD structure in the genome. Also, those *P*-values might be based on samples of different size, obtained using variable recruitment strategies, and might originate diverging analytic choices with respect to data cleaning and inclusions of covariates. Second, KGG v3.0 facilitates estimation of the genetic correlation matrix from a reference population like HapMap: actual genotypes of study samples do not need to be available. However, when the LD structure of the reference population differs from the LD in the actual study sample, MGAS can be liberal or conservative: we refer to Li *et al.* (2011) for an extensive discussion. Third, the phenotypic correlation matrix used in MGAS should be corrected for the same covariates that are used in the univariate association analyses that provide the input for MGAS (i.e. the original phenotypic scores need to be regressed on these covariates and the correlations between the residuals feature as input for MGAS).

Traits that have to date yielded few results in GWAS are often believed to be genetically complex, i.e. they involve many genes of small effect. However, genome-wide studies of complex traits have so far almost invariably relied on composite scores like sum scores or case–control dichotomies, thereby assuming the true trait-generating genotype–phenotype model to be a 1-factor model with the gene-effects on the factor (Fig. 1A). Our previous (Van der Sluis *et al*., 2010, 2013) and current simulations show that the abundance of null-results in the GWAS literature is not only in line with the hypothesis that many traits are genetically complex: the difficulty to identify associated genes could also indicate that the trait-generating genotype–phenotype model is misspecified. Here we showed that MGAS, which is exploratory in the sense that it does not require researchers to commit to one trait-generating genotype–phenotype model before conducting the association analysis, has excellent power under various trait-generating models. As such, MGAS allows researchers to conduct their multivariate gene-based analyses efficiently, and without the loss of power that is often associated with an incorrect trait-generating genotype–phenotype model. 

## Supplementary Material

Supplementary Data
