# Supplementary material for: MGAS: a powerful tool for multivariate gene-based genome-wide association analysis
Source: Bioinformatics. 2014 Nov 26;31(7):1007–15. doi: 10.1093/bioinformatics/btu783 (PMC4382905; doi:10.1093/bioinformatics/btu783)
Supplement: Supplementary Data [file supp_31_7_1007__index.html]

MGAS: a powerful tool for multivariate gene-based genome-wide association analysis — MGAS: a powerful tool for multivariate gene-based genome-wide association analysis — MGAS: a powerful tool for multivariate gene-based genome-wide association analysis — Supplementary Data 

# MGAS: a powerful tool for multivariate gene-based genome-wide association analysis

## Supplementary Data

files

**Files in this Data Supplement:**

- Supplementary Data - pdf file
