# Supplementary material for: MGAS: a powerful tool for multivariate gene-based genome-wide association analysis
Source: Bioinformatics. 2014 Nov 26;31(7):1007–15. doi: 10.1093/bioinformatics/btu783 (PMC4382905; doi:10.1093/bioinformatics/btu783)
Supplement: Supplementary Data [file supp_btu783_Supplement_BioInf_total_rev.pdf]

Supplement

MGAS: a powerful tool for multivariate gene-based genome-wide association analysis

Sophie van der Sluis[1]

Conor V. Dolan[2]

Jiang Li[3]

Youqiang Song[3,7,8,9]

Pak Sham[6,7,8,9]

Danielle Posthuma[1,4,5]

Miao-xin Li[6,7,8,9*]

[1] Department of Complex Trait Genetics, VU Medical Center (VUmc), Center for Neurogenomics and Cognitive Research (CNCR), Neuroscience Campus Amsterdam, De Boelelaan 1085, 1081 HV Amsterdam, the Netherlands

[2] Department of Biological Psychology, VU University Amsterdam, Van der Boechorststraat 1, 1081 BT Amsterdam, The Netherlands

[3] Department of Biochemistry, the University of Hong Kong, Pokfulam, Hong Kong

[4] Department of Functional Genomics, Center for Neurogenomics and Cognitive Research (CNCR), Neuroscience Campus Amsterdam, VU University, Amsterdam, The Netherlands

[5] Department of Child and Adolescent Psychiatry, Erasmus MC-University Medical Centre Rotterdam, Sophia Hospital Rotterdam, The Netherlands

[6] Department of Psychiatry, the University of Hong Kong, Pokfulam, Hong Kong

[7] State Key Laboratory for Cognitive and Brain Sciences, the University of Hong Kong, Pokfulam, Hong Kong

[8] The Centre for Reproduction, Development and Growth, the University of Hong Kong, Pokfulam, Hong Kong

[9] The Centre for Genomic Sciences, the University of Hong Kong, Pokfulam, Hong Kong

*Correspondence should be addressed to Miao-Xin Li (Tel: +852-2831 5105; E-mail: mxli@hku.hk)

Content

Supplemental Information Simulations p3

Figure S1: The relationship between r*ρ (i.e., correlations between genotypes r and correlations between phenotypes ρ: x-axis) and correlations between p-values (y-axis) p8

Figure S2: Example QQplots of 4 whole-genome multivariate gene-based Type I error rate simulations in which real genome-wide genetic data were used and simulated phenotypes with a correlational structure similar to that of the NFBC1966 metabolic phenotypes (Table S8). p9

Figure S3: QQplot of the orginal p-values (provided by the authors (Sabatti et al) and available in the dbGaP data base) per phenotype. p10

Figure S4: QQplot of the univariate SNP-based p-values (combined across all phenotypes), subdivided in all SNPs (green), SNPs in genes (orange), and SNPs outside genes (pink). The multivariate gene-based p-values calculated by MGAS are printed in blue. p11

Table S1a: Visual representation of the LD patterns used for the simulations including small genes (10 SNPs) p12

Table S1b: Visual representation of the LD patterns used for the simulations including small genes (10 SNPs) p13

Table S1c: Visual representation of the LD patterns used for the simulations including large genes (60 SNPs) p14

Table S1d: Visual representation of the LD patterns used for the simulations including large genes (60 SNPs) p15

Table S1e: Visual representation of the LD patterns used for the simulations including large genes (60 SNPs) p16

Table S2: 1 factor model, gene-effect on factor p17

Table S3: 1 factor model, gene-effect specific to one phenotype p18

Table S4: 4 factor model, gene-effect on one of the four factors p19

Table S5: 4 factor model, gene-effect specific to one phenotype p20

Table S6: Network model mimicking 1-factor model (i.e., all phenotypes equally strongly related), gene-effect specific to one phenotype p21

Table S7: Network model mimicking 4-factor model (i.e., clusters of phenotypes), gene-effect specific to one phenotype p22

Table S8: Phenotypic correlations between 9 metabolic traits, corrected for sex, oral contraceptive use and pregnancy p23

Table S9: List of FDR corrected genes/genetic regions identified by MGAS in the metabolic data

Table S10: Annotation of the genes/genetic regions identified by MGAS by reference to the Catalog of Published Genome-wide Association Studies (http://www.genome.gov/gwastudies/)

Table S11: Genome-wide significant SNPs identified by TATES using the 9 continuous metabolic phenotypes

Table S12: Genome-wide significant genes identified by GATES on a) the univariate sum score of five metabolic syndrome risk factors and b) the individual five dichotomous metabolic risk factors

Table S13: Genome-wide significant genes identified by GATES on the 9 continuous metabolic phenotypes separately

Table S14: Comparison results MGAS, TATES, and GATES when all analyses are conducted on the original 9 continuous metabolic phenotypes

Simulations

General

All simulations (results shown in Figure 2 and Supplementary Tables S2-S7) concerned N=2000 unrelated subjects and 20 standard normally distributed phenotypes. Simulated genes, covering 10 or 60 SNPs all with MAF=.2, explained 0, .5% or 1% of the variance, either on the latent level, or in a specific phenotype (see below). All simulations were repeated N_{rep}=2000 times.

Note that the standard error of the ML estimator of the p-value equals SE=√ ((p*(1-p))/N_{sim}), where p denotes the percentage of significant tests observed in the simulations (nominal p-value) given the chosen α. The 95% confidence interval for an unbiased nominal p-value when the gene-effect is actually zero (i.e., given α=.05, we expect p≈.05) corresponds to CI_{95}=(p -1.96*SE, p +1.96*SE). Given N_{rep}=2000 and α=.05, CI_{95} thus equals .0409 - .0605, i.e., p-values < .04 suggest that the MGAS-procedure is too conservative, while p-values >.06 suggest that the MGAS-procedure is too liberal.

Simulations and analyses were conducted in *R* (R Development Core Team, 2011, version 2.13.0), and run on the Genetic Cluster Computer.

Genetic data

Data were simulated for either a small gene (covering 10 SNPs) or a larger gene (covering 60 SNPs). All SNPs had minor allele frequency (MAF) of .20. Data were simulated such that within LD blocks, r=.9 (i.e., r^2=.81), and between LD blocks, r=0 (i.e., SNPs in different blocks did not correlate).

All genetic simulations settings are summarized in Table 1 of the main manuscript. For the small gene, 10 scenarios were simulated, with different numbers of Disease Susceptibility Loci (DSL, i.e., a SNP that is causally related to one or more phenotypes):

1. all SNPs in 1 LD block with 0 DSL (to control Type I error rate);
2. all SNPs in 1 LD block, 1 DSL;
3. all SNPs in 1 LD block, 1 DSL with opposite effects*;
4. all SNPs in 1 LD block, 2 DSL;
5. all SNPs in 1 LD block, 2 DSL with opposite effects**;
6. all SNPs in 1 LD block, 4 DSL;
7. all SNPs in 1 LD block, 4 DSL with opposite effects**;
8. SNPs divided over 2 LD blocks (5 SNPs each), 1 DSL in first block;
9. SNPs divided over 2 LD blocks (5 SNPs each), 1 DSL per block;
10. SNPs divided over 2 LD blocks (5 SNPs each), 1 DSL per block, opposite effects**.

For the large gene, 4 scenarios were simulated. In each of these scenarios, the gene covered 8 LD blocks: 4 blocks of 10 SNPs each, and 4 blocks of 5 SNPs each.

1. 0 DSL (to control Type I error rate);
2. 1 DSL in a large block;
3. 8 DSL, 1 in each LD block;
4. 8 DSL, 1 in each block, opposite effects**.

* In factor models (Figure 1a,c), the one DSL affected half of the phenotypic indicators of a factor positively and the other half negatively. In unclustered networks (Figure 1e), the one DSL affected 1 phenotype positively and another phenotype in the network negatively. In clustered networks (Figure 1f), the one DSL affected either 2 phenotypes in the same cluster, or 2 phenotypes in different clusters, in opposite directions.
** Half of the DSL in a gene conveyed a positive effect, the others a negative effect on the factors (Figure 1a,c) or phenotypes (Figure 1c,d,e,f).

See Supplemental Tables S1a-e for a visual representation of these LD settings.

Note that, assuming the same number of DSL with the same effect size, the power to detect a gene is negatively correlated to the size of the gene. Specifically, because the weight q_e, denoting the effective number of p-values (i.e., tests and thus the effective number of phenotypes + SNPs), will, given the same LD structure, invariably be higher for larger genes, the weight q_e/q_{ej} will also be higher. As a consequence, p-values of DSL with the same effect size will be multiplied by a larger weight when these DSL are located in a larger gene. That is, a stronger genetic signal is required to implicate a large gene. To obtain illustrative power results, we therefore chose to simulate the data such that the DSL in small genes together explained .5% of the variance, and the DSL in large genes together explained 1% variance. As a consequence, power results as depicted in Figure 2 and reported in Tables S2-S7 are not directly comparable across genes of different size.

When a simulated gene included multiple DSL, the effect was evenly divided over the multiple DSL (e.g., in a large gene with 2 DSL, both DSL were simulated to explain 1/2=.5% variance). Important to note is that because of the strong LD between the SNPs within an LD block, SNPs that were simulated to not have an effect are related to the phenotype if one regresses the phenotype on that SNP because of that SNP's strong LD with the actual DSL(s) in the block (i.e., the regression coefficient β in that regression will be equal to the β of the actual DSL weighted by the LD measure

r). In addition, when multiple DSL are simulated within a block, the effect of every DSL is augmented in the simple regression of a phenotype on that DSL because of the DSL's correlation to other DSL in a block (i.e., the β in that regression is equal to the simulated β of that DSL plus the β of other DSL in the block weighted by the LD measure r). As a consequence, a) the variance explained by the entire gene (the R^2 as calculated in e.g. multiple regression) was, given our simulation settings, larger for genes harbouring multiple DSL, and b) in the simple regression of a phenotype on a single DSL, the variance explained by the DSL was almost equal across scenarios 2, 3 and 4 of the small gene (i.e., when 1, 2 or 4 DSL resided in the same LD-block).

<u>*Phenotypic data*</u>

Phenotypic factor models

With m phenotypes and k common factors, phenotypic data were simulated according to the following model:

$$\Sigma = \Lambda\Psi\Lambda^t + \Theta, \tag{4}$$

where $\mathbf{\Sigma}$ denotes the mxm phenotypic variance-covariance matrix, $\mathbf{\Lambda}$ denotes the mxk matrix of factor loadings (with ^{t} denoting matrix transpose), $\mathbf{\Psi}$ denotes the kxk covariance matrix between the k latent factors, and $\mathbf{\Theta}$ denotes the diagonal mxm matrix of residual variances (i.e., the part of the phenotypic variances that is not explained by any of the k factors). In models with k>1, simple structure was maintained (i.e., all phenotypes loaded on only 1 latent factor).

Data were generated for models featuring either 1 or 4 factors. In the 1-factor models, factor loadings were set to .75, such that the latent factor explained $.75^2$=.56 % of the variance in each phenotype, and residual variances were equal to 1-$.75^2$=.44. Consequently, all intercorrelations between the 20 standard normally distributed phenotypes were .56. In the 1-factor model, all SNPs within a gene were either modelled to affect the latent factor (and through the latent factor all 20 phenotypes underlying the factor), or to affect one of the 20 phenotypes directly (see Figures 1a and 1b, respectively).

Note that when 1) all phenotypic intercorrelations are explained by 1 latent factor, 2) all phenotypes have equal factor loadings, and 3) all phenotypes have equal residual variances, the model is called a Rasch model (Rasch, 1980). Important to note is that *only* when the phenotypic data-generating model is a Rasch model *and* the effect of gene X is on the latent factor (i.e., Figure

1a), is the sum score calculated across all individual phenotypes a sufficient statistic for the genetic analysis of gene X, i.e., only then does the sum of scores on all phenotypes exhaustively summarize all phenotypic information that is relevant for the analysis testing the association with gene X. The sum score is, however, not a sufficient statistic for the genetic analysis when the genetic effect is directly on one of the phenotypes, or when the phenotypic Rasch model does not hold.

In the 4-factor models, 5 phenotypes were modelled per factors. All factor loadings were set to .75 within factors, all residual variances equalled $1\text{-}.75^2=.44$, and correlations between factors were set to .23. Consequently, correlations between phenotypes loading on the same factor equalled $.75^2=.56$, while correlations between phenotypes loading on different factors equalled .75*.23*.75=.13. Effects of all SNPs within a gene were either modelled on one of the four latent factors (and through that factor on all 5 phenotypes defining that factor), or directly on one of the phenotypes (see Figures 1c and 1d, respectively). Note that the phenotypic sum score is not a sufficient statistic for the genetic analysis when the phenotypic model is a 4-factor model.

Phenotypic network models

In all network simulations, we assumed the model to be stationary, i.e., the mutual interactions between the phenotypes have over time resulted in a stable variance-covariance matrix. Also, we assumed that all phenotypes were mutually related, i.e., in all regressions of the phenotypes on each other $\beta_{ij}\neq0$. We did, however, not allow instantaneous self-activation, i.e., the phenotypes were not allowed to affect themselves on the next time point (i.e., $\beta_{ii}=0$). (Note, however, that indirect self-activation on subsequent time points was allowed, i.e., phenotype A affects phenotype B on time point t=1, and as all phenotypes were simulated to be mutually related, it affects itself *via* phenotype B on t=2). Network data were simulated according to the model:

$$\Sigma = (I - B)^{-1}\Psi(I - B)^{-1t}, \qquad (5)$$

where **Σ** denotes the *mxm* phenotypic variance-covariance matrix, **I** is an *mxm* identity matrix, **B** is a full *mxm* matrix including the regression weights *β* of all phenotypes on each other (i.e., element **B**[i,j] includes the regression weight of phenotype *i* on phenotype *j*). The diagonal of **B** was fixed to zero to model absence of instantaneous self-activation. **Ψ** is the *mxm* diagonal matrix containing the variances of all phenotypes conditional on the other phenotypes.

Note that in network models, in contrast to the factor models, phenotype-specific genetic effects propagate through the network to indirectly affect other connected phenotypes in the

network. In phenotypic network models, genes thus affect all connected phenotypes, with the size of the effect depending on the strength of the phenotypic relations (i.e., edges) in the network. In all network simulations, all SNPs within a gene affected only the first phenotype in the network, but because all phenotypes are connected, the genetic effects do spread through the network. Two types of networks were simulated. First, all regression weights in the B matrix were set to .04197, resulting in phenotypic intercorrelations among all 20 phenotypes of .56. That is, the phenotypic variance-covariance matrix of these network data is similar to the phenotypic variance-covariance matrices resulting from the 1-factor Rasch models discussed above. Second, a network was simulated with 4 clusters of phenotypes that correlated .56 within clusters, and .13 between clusters, resulting in a phenotypic variance-covariance matrix that is similar to that resulting from the 4-factor models discussed above. The network models are illustrated in Figures 1e and 1f, respectively).

It is important to note that network models and factor models can yield phenotypic data with very similar variance-covariance structure, in spite of the different underlying data-generating processes. As a consequence, even if the variance-covariance structure of data can be well described by a 1-factor model, this does not necessarily imply that the 1-factor model is the actual data-generating model. This is relevant for genetic studies, because factor analytic results are often used to justify the reduction of multivariate data to univariate composite scores (e.g., sum scores, factor scores). Such reduction is, however, only valid if the data-generating process is a 1-factor model, but not if the data are generated by a network process.

R Development Core Team (2011). R: A language and environment for statistical computing. R Foundation for Statistical Computing, Vienna, Austria. ISBN 3-900051-07-0, URL http://www.R-project.org/.

Rasch G (1980) Probabilistic models for some intelligence and attainment tests. Chicago: The University of Chicago Press.

Figure S1

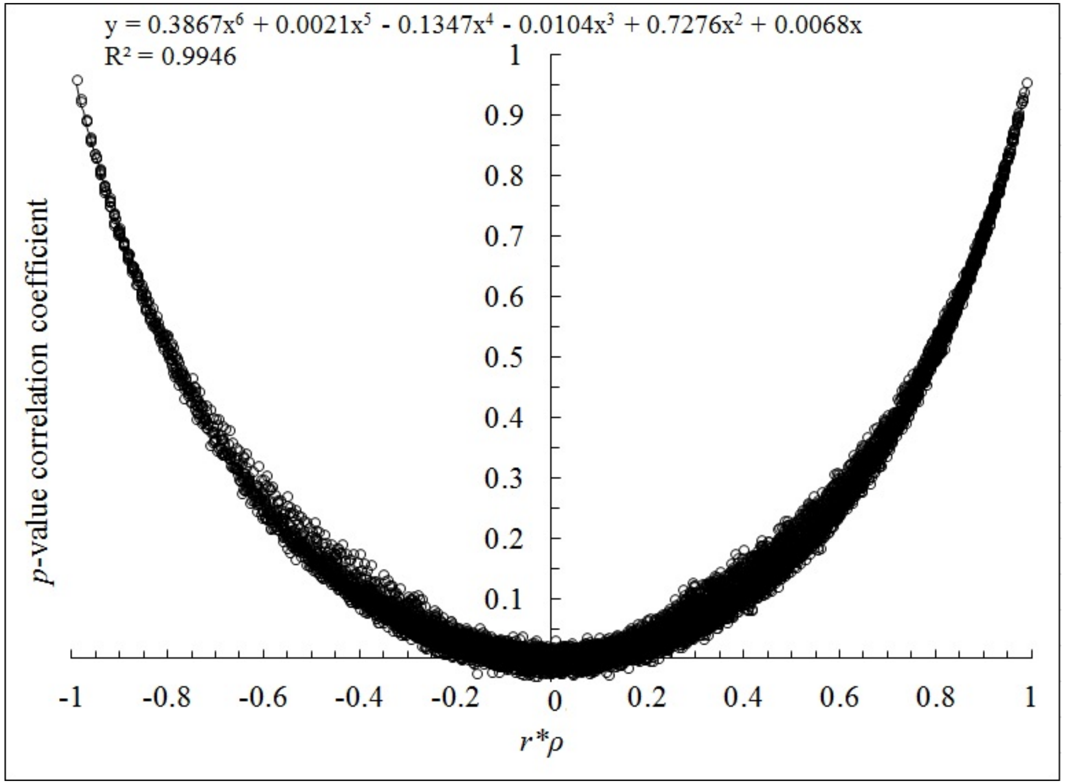

The relationship between $r*\rho$ (i.e., correlations between genotypes r and correlations between phenotypes ρ: x-axis) and correlations between p-values (y-axis) obtained in the regression of a phenotype on a genetic variant. Simulations (see original manuscript §2.2 for details) show that this relationship can be accurately described by a 6th order polynomial (coefficient of determination R^2=.995). Note that the data points produced for the quantitative and qualitative traits are mixed together to generate a unified approximate function. Note also that using the same 6th order polynomial, similarly high R^2 values were obtained for different simulation settings. For instance, when the simulated sample size was only N=2000, the R^2 was 0.998, and when 4 phenotypes and 4 SNPs were simulated in a sample of 4000 subjects, the R^2 was 0.997.

Figure S2

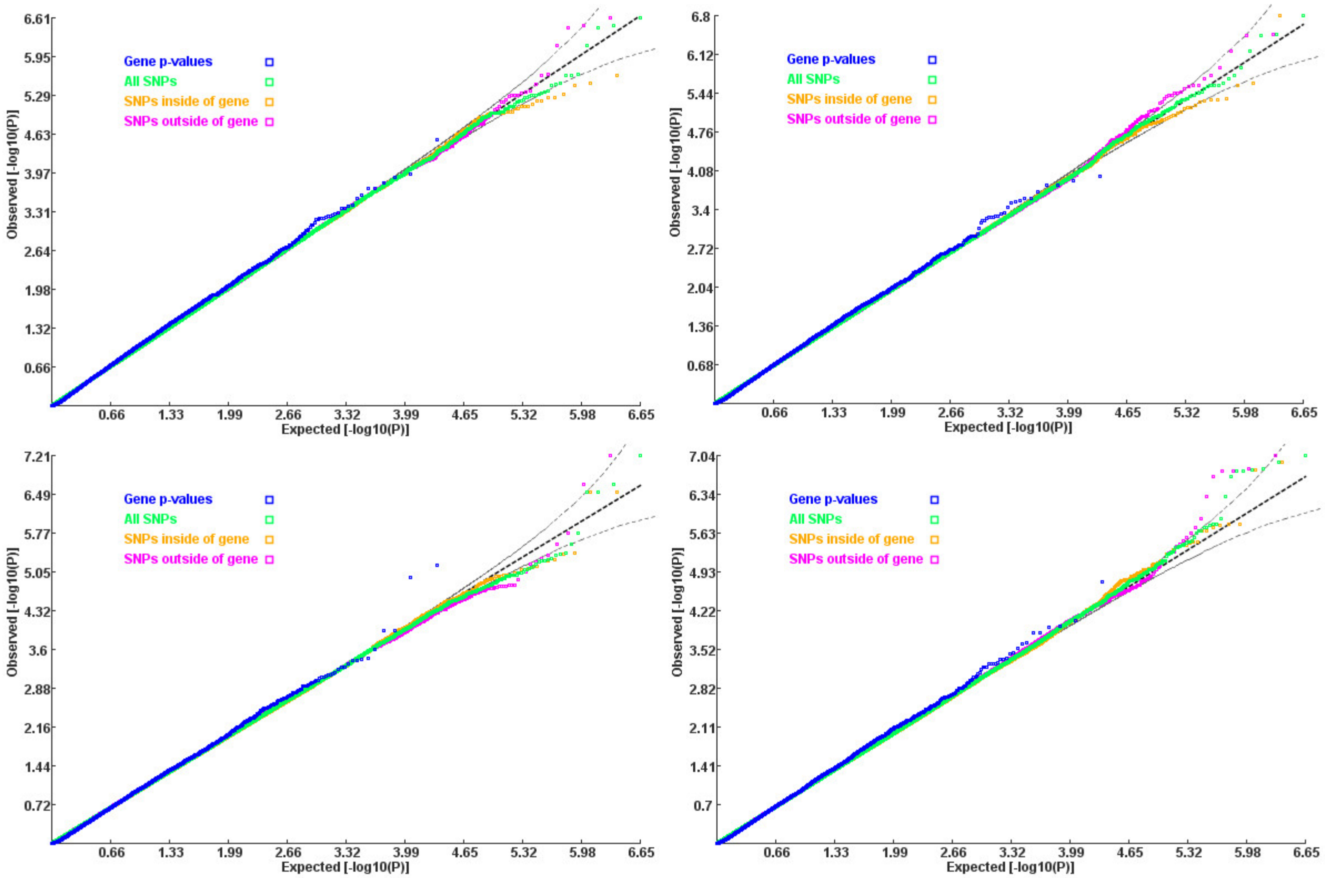

Example QQplots of 4 of the 10 whole-genome multivariate gene-based simulations in which we used real genome-wide genetic data of N=4,763 subjects from the Northern Finland Birth Cohort (NFBC1966, Sabatti *et al.*, 2009), and simulated 10 sets of 9 phenotypes, of which the correlational structure mimicked the observed correlations of the NFBC1966 metabolic phenotypes (see Supplemental Table S8), to check whether MGAS Type I error rates were correct regardless of LD pattern and gene size. Plotted are the univariate SNP-based p-values (i.e., p-values of all 9 phenotypes in one plot), subdivided in all SNPs (green), SNPs in genes (orange), and SNPs outside genes (pink). The multivariate gene-based p-values (blue) are calculated through the MGAS procedure and are assembled from the SNPs located within genes only.

Figure S3

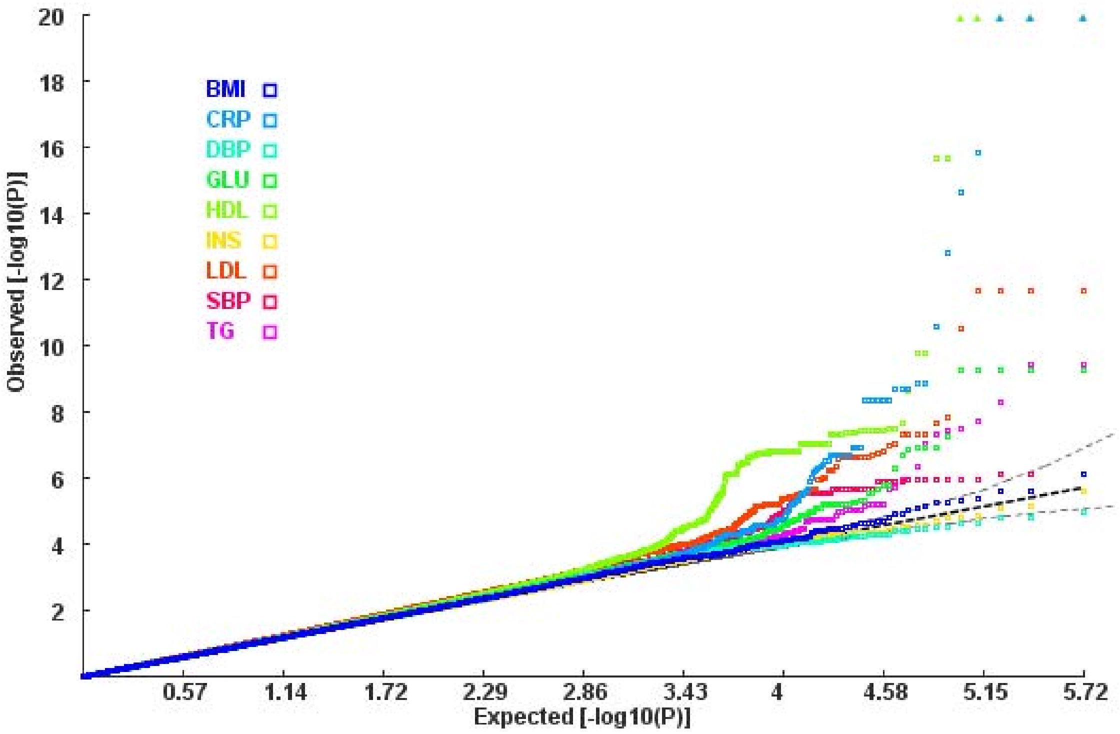

QQplot of the orginal p-values per phenotype obtained in covariate and PC corrected analyses, available in the dbGaP data base as provided by the original authors:

Sabatti, C., Service, S.K., Hartikainen, A-L, Pouta, A., Ripatti, S., Brodsky, J., et al. Genome-wide association analysis of metabolic traits in a birth cohort from a founder population. Nat. Genet., 41, 35-46 (2009).

Figure S4

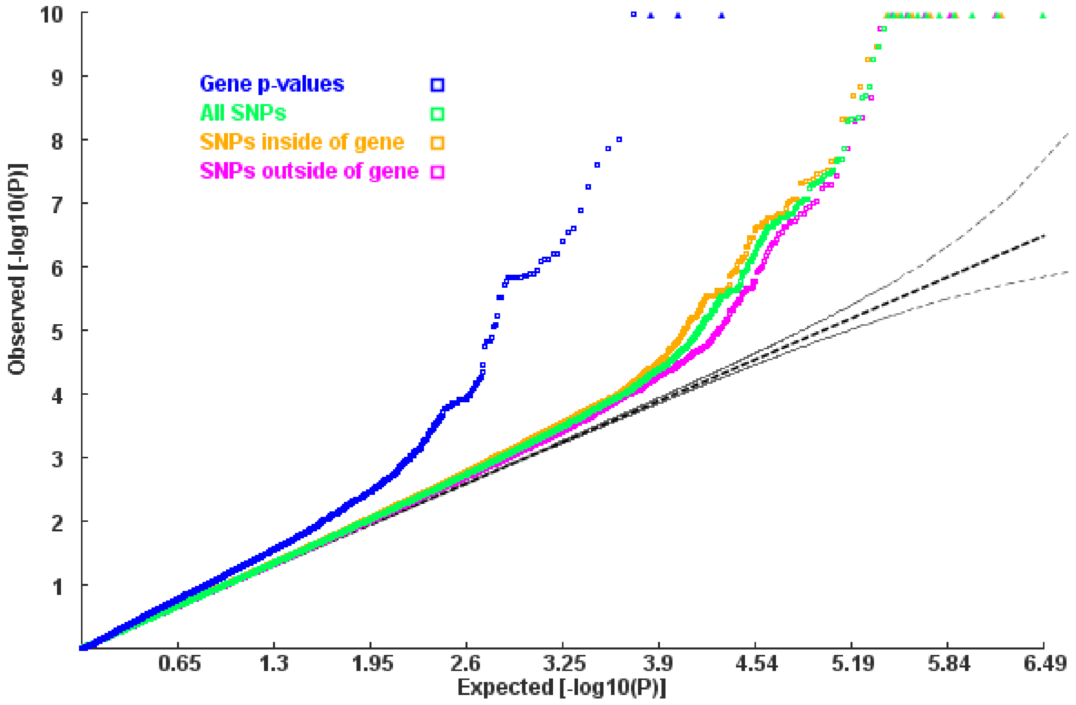

QQplot of the univariate SNP-based p-values (i.e., p-values of all 9 phenotypes in one plot), subdivided in all SNPs (green), SNPs in genes (orange), and SNPs outside genes (pink). The multivariate gene-based p-values (blue) are calculated through the MGAS procedure and are assembled from the SNPs located within genes only: the enrichment of association signals in the multivariate gene-based p-values is due to the selection of smallest weighted p-values by MGAS.

Table S1a
Visual representation of the LD patterns used for the simulations including small genes (10 SNPs)

	Gene 1										Gene 2										Gene 3									
Scenario 1	0 DSL (to control Type I error rate)																													
	1																													
	.9	1																												
	.9	.9	1																											
	.9	.9	.9	1																										
	.9	.9	.9	.9	1																									
	.9	.9	.9	.9	.9	1																								
	.9	.9	.9	.9	.9	.9	1																							
	.9	.9	.9	.9	.9	.9	.9	1																						
	.9	.9	.9	.9	.9	.9	.9	.9	1																					
	.9	.9	.9	.9	.9	.9	.9	.9	.9	1																				
Scenario 2/3											1 DSL (in red)																			
											1																			
											.9	1																		
											.9	.9	1																	
											.9	.9	.9	1																
											.9	.9	.9	.9	1															
											.9	.9	.9	.9	.9	1														
											.9	.9	.9	.9	.9	.9	1													
											.9	.9	.9	.9	.9	.9	.9	1												
											.9	.9	.9	.9	.9	.9	.9	.9	1											
											.9	.9	.9	.9	.9	.9	.9	.9	.9	1										
Scenario 4/5																					2 DSL (in red)									
																					1									
																					.9	1								
																					.9	.9	1							
																					.9	.9	.9	1						
																					.9	.9	.9	.9	1					
																					.9	.9	.9	.9	.9	1				
																					.9	.9	.9	.9	.9	.9	1			
																					.9	.9	.9	.9	.9	.9	.9	1		
																					.9	.9	.9	.9	.9	.9	.9	.9	1	
																					.9	.9	.9	.9	.9	.9	.9	.9	.9	1

Note. Within blocks (grey shading) r=.9, between block (no shading), r=0. Disease Susceptibility Loci (DSL) are indicated in red. All small genes including DSL explain .5% of the phenotypic variance; when a gene includes multiple DSL, the .5% is evenly distributed across all DSL (e.g., in scenario 3, the 2 DSL each explain .25% of the variance). The scenario numbers refer to those in Table 1 of the original manuscript.

Table S1b
Visual representation of the LD patterns used for the simulations including small genes (10 SNPs)

	Gene 4										Gene 5										Gene 6									
Scenario 6/7	4 DSL (in red)																													
	1																													
	.9	1																												
	.9	.9	1																											
	.9	.9	.9	1																										
	.9	.9	.9	.9	1																									
	.9	.9	.9	.9	.9	1																								
	.9	.9	.9	.9	.9	.9	1																							
	.9	.9	.9	.9	.9	.9	.9	1																						
	.9	.9	.9	.9	.9	.9	.9	.9	1																					
	.9	.9	.9	.9	.9	.9	.9	.9	.9	1																				
Scenario 8											1 DSL (in red)																			
											1																			
											.9	1																		
											.9	.9	1																	
											.9	.9	.9	1																
											.9	.9	.9	.9	1															
																1														
																.9	1													
																.9	.9	1												
																.9	.9	.9	1											
																.9	.9	.9	.9	1										
Scenario 9/10																					2 DSL (in red)									
																					1									
																					.9	1								
																					.9	.9	1							
																					.9	.9	.9	1						
																					.9	.9	.9	.9	1					
																										1				
																										.9	1			
																										.9	.9	1		
																										.9	.9	.9	1	
																										.9	.9	.9	.9	1

Note. Within blocks (grey shading) r=.9, between block (no shading), r=0. Disease Susceptibility Loci (DSL) are indicated in red. All small genes including DSL explain .5% of the phenotypic variance; when a gene includes multiple DSL, the .5% is evenly distributed across all DSL (e.g., in scenario 4, the 4 DSL each explain .125% of the variance). The scenario numbers refer to those in Table 1 of the original manuscript.

Table S1c

Visual representation of the LD patterns used for the simulations including larger genes (60 SNPs)

Scenario 11: 0 Disease Susceptibility Loci (to control Type I error rate)

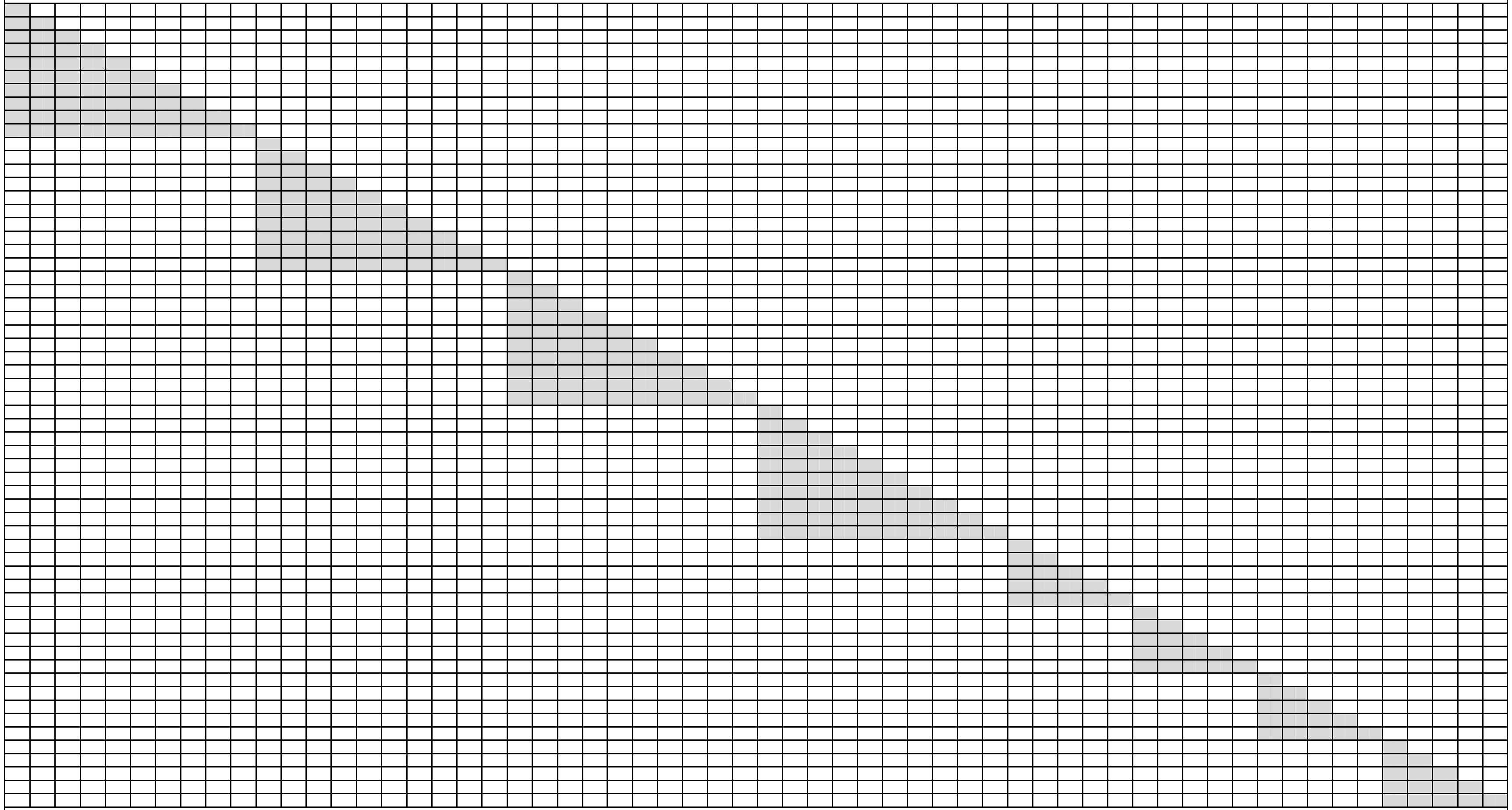

Note. A gene covering 60 SNPs divided over 4 large (10 SNPs) and 4 small (5 SNPs) LD blocks. Within blocks (grey shading) r=.9, between block (no shading), r=0.

The scenario numbers refer to those in Table 1 of the original manuscript.

Table S1d

Visual representation of the LD patterns used for the simulations including large genes (60 SNPs)

Scenario 12: 1 Disease Susceptibility Locus (in red) explaining 1% of the variance

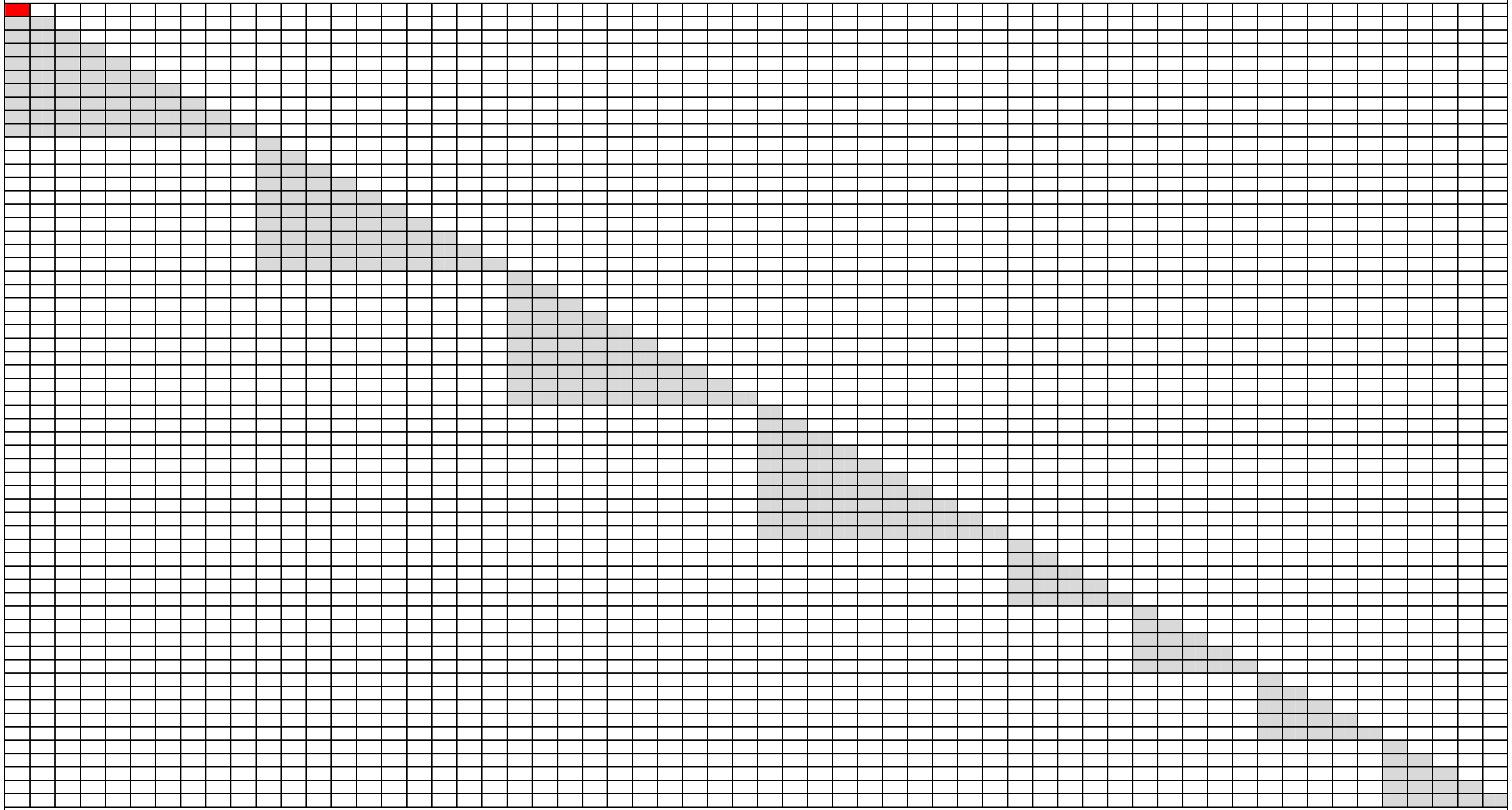

Note. A gene covering 60 SNPs in 4 large (10 SNPs) and 4 small (5 SNPs) LD blocks. Within blocks (grey shading) r=.9, between block (no shading), r=0. Disease Susceptibility Loci (DSL) are indicated in red. The scenario numbers refer to those in Table 1 of the original manuscript.

Table S1e

Visual representation of the LD patterns used for the simulations including large genes (60 SNPs)

Scenario 13/14: 8 Disease Susceptibility Loci (in red) together explaining 1% of the variance (i.e., .125% each)

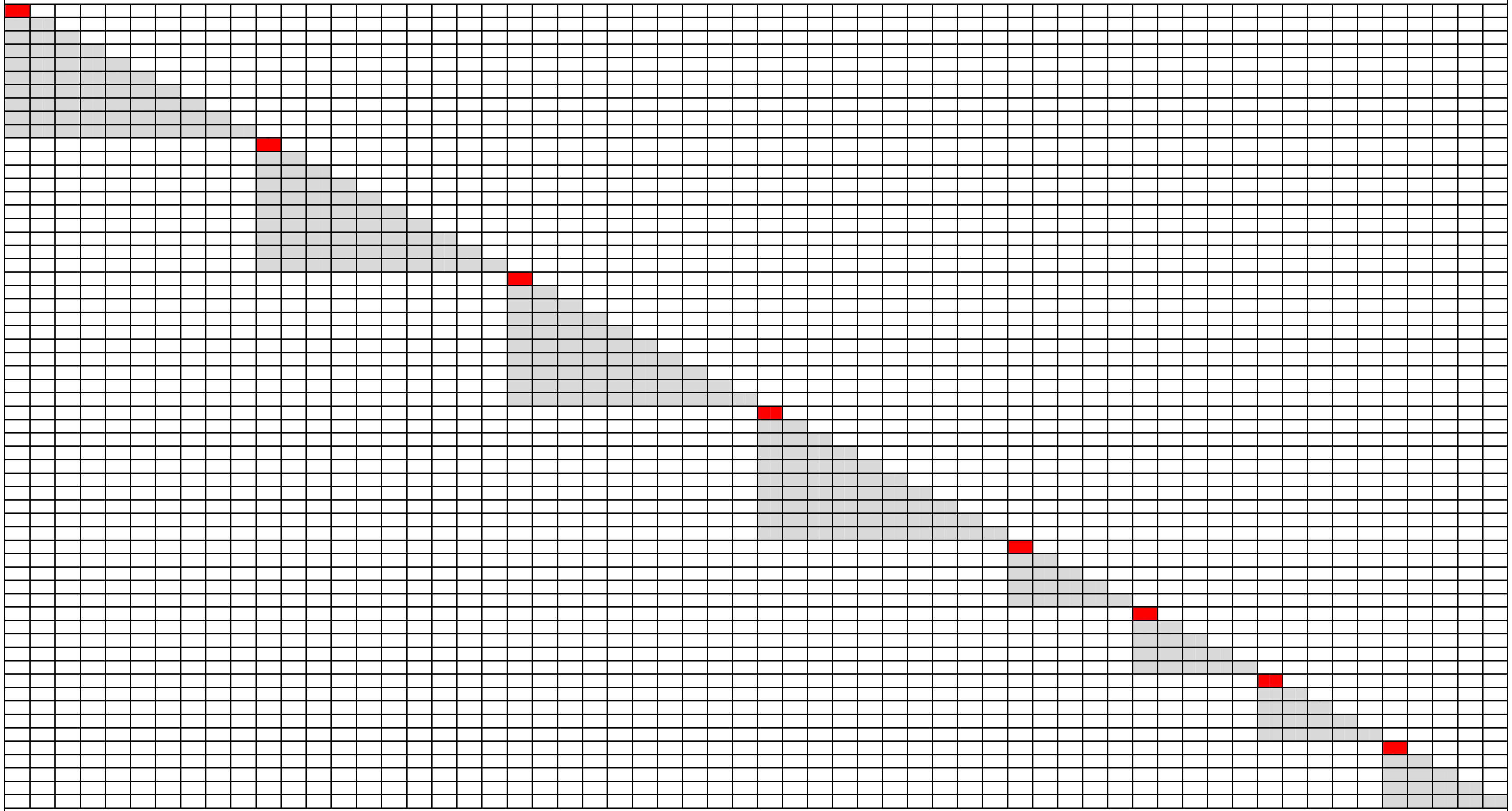

Note. A gene covering 60 SNPs in 4 large (10 SNPs) and 4 small (5 SNPs) LD blocks. Within blocks (grey shading) r=.9, between block (no shading), r=0. Disease Susceptibility Loci (DSL) are indicated in red. The scenario numbers refer to those in Table 1 of the original manuscript.

Table S2

1 factor model, gene-effect on factor

		GATES on sum	GATES on MANOVA	MANOVA	Multiple regression on sum	MGAS
Small gene (10 SNPs; .5% explained)	Scenario 1 *1 block, 0 DSL*	**0.0430**	**0.0445**	**0.0485**	**0.0470**	**0.0435**
	Scenario 2 (IV)[*] *1 block, 1 DSL*	0.8270	0.3280	0.1215	0.5145	0.7750
	Scenario 3 (X) *1 block, 1 DSL (opp[***])*	0.0460	1.0000	1.0000	0.0515	1.0000
	Scenario 4 (V) *1 block, 2 DSL*	0.9830	0.6745	0.2075	0.8465	0.9655
	Scenario 5 *1 block, 2 DSL (opp[**])*	0.0455	0.0460	0.0605	0.0880	0.0445
	Scenario 6 (VI) *1 block, 4 DSL*	1.0000	0.9605	0.4610	0.9965	1.0000
	Scenario 7 *1 block, 4 DSL (opp[**])*	0.0550	0.0465	0.0580	0.0790	0.0530
	Scenario 8 (VII) *2 blocks, 1 DSL*	0.7745	0.2805	0.1175	0.5125	0.7045
	Scenario 9 (VIII) *2 blocks, 2 DSL*	0.6945	0.2135	0.1145	0.5030	0.6070
	Scenario 10 (IX) *2 block, 2 DSL (opp[**])*	0.7170	0.2240	0.1300	0.5165	0.6355
Large gene (60 SNPs; 1% explained)	Scenario 11 *8 blocks, 0 DSL*	**0.0545**	**0.0585**	**0.0520**	**0.0555**	**0.0555**
	Scenario 12 (I) *8 blocks, 1 DSL*	0.9255	0.4280	0.1130	0.4585	0.8740
	Scenario 13 (II) *8 blocks, 8 DSL*	0.6075	0.1525	0.1000	0.4665	0.5250
	Scenario 14 (III) *8 blocks, 8 DSL (opp[**])*	0.6185	0.1565	0.0940	0.4690	0.5440

Note. DSL=Disease Susceptibility Loci. All Nrep=2000 simulations included N=2000 subjects, Nphen=20 phenotypes, and small or large genes covering N_{SNP}=10 or N_{SNP}=60 SNPs, respectively. Simulated data were analyzed in 5 ways all yielding a gene-based p-value. Sum Gates: original Gates procedure conducted on the univariate sum score calculated across the 20 phenotypes; Sum multiple regression: univariate phenotypic sum score regressed on all SNPs within a gene; MANOVA: all 20 phenotypes as dependent variables and all SNPs within a gene as predictors; GATES-MANOVA: all 20 phenotypes as dependent variables and each SNP separately as predictor in MANOVA, the N_{SNP} MANOVA p-values are then combined using GATES; MGAS: weighted selection of the *mxn* p-values obtained in regression of all *m* phenotypes on all *n* SNPs within a gene, while correcting for the expected correlations between these p-values resulting from the phenotypic correlations and the LD-structure within a gene. MAF was .2 for all simulated SNPs. Within and between LD-blocks, LD measure *r* was set to .9 and to 0, respectively. As the MGAS procedure has less power to detect larger genes (see Supplemental Information), small genes and large genes explained .5% and 1% of the variance, respectively. As a consequence, power results are not directly comparable between small and large genes. When a gene harboured multiple DSL, the total gene effect was equally distributed across all DSL.

[*] The Roman numerals refer to Figure 2 in the paper. [**] Half of the DSL in a gene conveyed a positive effect, the others a negative effect on the factors (Figure 1a,c) or phenotypes (Figure 1c,d,e,f). [***] In latent factor models (Figure 1a,c), the one DSL affected half of the phenotypic indicators of a factor positively and the other half negatively. In unclustered networks (Figure 1e), the one DSL affected 1 phenotype positively and another phenotype in the network negatively. In clustered networks (Figure 1f), the one DSL affected either 2 phenotypes in the same cluster, or 2 phenotypes in different clusters, in opposite directions.

Table S3

1 factor model, gene-effect specific to one phenotype

		GATES on sum	GATES on MANOVA	MANOVA	Multiple regression on sum	MGAS
Small gene (10 SNPs; .5% explained)	Scenario 1 *1 block, 0 DSL*	**0.0505**	**0.0495**	**0.0450**	**0.0550**	**0.0495**
	Scenario 2 (IV)* *1 block, 1 DSL*	0.0485	0.7110	0.2665	0.0560	0.5075
	Scenario 4 (V) *1 block, 2 DSL*	0.0510	0.9795	0.5435	0.0540	0.8990
	Scenario 5 *1 block, 2 DSL (opp**)*	0.0435	0.0475	0.0595	0.0570	0.0405
	Scenario 6 (VI) *1 block, 4 DSL*	0.0560	1.0000	0.9390	0.0520	0.9985
	Scenario 7 *1 block, 4 DSL (opp**)*	0.0470	0.0535	0.0665	0.0485	0.0485
	Scenario 8 (VII) *2 blocks, 1 DSL*	0.0500	0.6720	0.2490	0.0415	0.4670
	Scenario 9 (VIII) *2 blocks, 2 DSL*	0.0615	0.4840	0.2445	0.0450	0.2855
	Scenario 10 (XI) *2 block, 2 DSL (opp**)*	0.0500	0.5220	0.2545	0.0455	0.2975
Large gene (60 SNPs; 1% explained)	Scenario 11 *8 blocks, 0 DSL*	**0.0515**	**0.0570**	**0.0495**	**0.0435**	**0.0585**
	Scenario 12 (I) *8 blocks, 1 DSL*	0.0615	0.9520	0.2275	0.0535	0.7800
	Scenario 13 (II) *8 blocks, 8 DSL*	0.0470	0.3370	0.2170	0.0530	0.1755
	Scenario 14 (III) *8 blocks, 8 DSL (opp**)*	0.0560	0.3685	0.2145	0.0555	0.2065

Note. DSL=Disease Susceptibility Loci. All Nrep=2000 simulations included N=2000 subjects, Nphen=20 phenotypes, and small or large genes covering N_{SNP}=10 or N_{SNP}=60 SNPs, respectively. Simulated data were analyzed in 5 ways all yielding a gene-based p-value. Sum Gates: original Gates procedure conducted on the univariate sum score calculated across the 20 phenotypes; Sum multiple regression: univariate phenotypic sum score regressed on all SNPs within a gene; MANOVA: all 20 phenotypes as dependent variables and all SNPs within a gene as predictors; GATES-MANOVA: all 20 phenotypes as dependent variables and each SNP separately as predictor in MANOVA, the N_{SNP} MANOVA p-values are then combined using GATES; MGAS: weighted selection of the *mxn* p-values obtained in regression of all *m* phenotypes on all *n* SNPs within a gene, while correcting for the expected correlations between these p-values resulting from the phenotypic correlations and the LD-structure within a gene. MAF was .2 for all simulated SNPs. Within and between LD-blocks, LD measure *r* was set to .9 and to 0, respectively. As the MGAS procedure has less power to detect larger genes (see Supplemental Information), small genes and large genes explained .5% and 1% of the variance, respectively. As a consequence, power results are not directly comparable between small and large genes. When a gene harboured multiple DSL, the total gene effect was equally distributed across all DSL.
[*] The Roman numerals refer to Figure 2 in the paper. [**] Half of the DSL in a gene conveyed a positive effect, the others a negative effect on the factors (Figure 1a,c) or phenotypes (Figure 1c,d,e,f). [***] In latent factor models (Figure 1a,c), the one DSL affected half of the phenotypic indicators of a factor positively and the other half negatively. In unclustered networks (Figure 1e), the one DSL affected 1 phenotype positively and another phenotype in the network negatively. In clustered networks (Figure 1f), the one DSL affected either 2 phenotypes in the same cluster, or 2 phenotypes in different clusters, in opposite directions.

Table S4
4 factor model, gene-effect on one of the four factors

		GATES on sum	GATES on MANOVA	MANOVA	Multiple regression on sum	MGAS
Small gene (10 SNPs; .5% explained)	Scenario 1 *1 block, 0 DSL*	**0.0465**	**0.0495**	**0.0510**	**0.0485**	**0.0445**
	Scenario 2 (IV)* *1 block, 1 DSL*	0.1765	0.3030	0.1215	0.1050	0.5290
	Scenario 3 (X) *1 block, 1 DSL (opp***)*	0.0530	1.0000	0.9965	0.0455	0.9925
	Scenario 4 (V) *1 block, 2 DSL*	0.3110	0.6680	0.2095	0.1455	0.8830
	Scenario 5 *1 block, 2 DSL (opp**)*	0.0425	0.0405	0.0555	0.0515	0.0555
	Scenario 6 (VI) *1 block, 4 DSL*	0.5465	0.9595	0.4950	0.2525	0.9955
	Scenario 7 *1 block, 4 DSL (opp**)*	0.0520	0.0420	0.0585	0.0545	0.0475
	Scenario 8 (VII) *2 blocks, 1 DSL*	0.1585	0.2660	0.1180	0.0985	0.4850
	Scenario 9 (VIII) *2 blocks, 2 DSL*	0.1605	0.2100	0.1340	0.1050	0.3685
	Scenario 10 (IX) *2 block, 2 DSL (opp**)*	0.1600	0.2205	0.1155	0.1065	0.4010
Large gene (60 SNPs; 1% explained)	Scenario 11 *8 blocks, 0 DSL*	**0.0460**	**0.0555**	**0.0425**	**0.0520**	**0.0565**
	Scenario 12 (I) *8 blocks, 1 DSL*	0.1625	0.4500	0.1135	0.0885	0.7295
	Scenario 13 (II) *8 blocks, 8 DSL*	0.1415	0.1585	0.0960	0.0860	0.2780
	Scenario 14 (III) *8 blocks, 8 DSL (opp**)*	0.1130	0.1570	0.1055	0.0785	0.2705

Note. DSL=Disease Susceptibility Loci. All Nrep=2000 simulations included N=2000 subjects, Nphen=20 phenotypes, and small or large genes covering N_{SNP}=10 or N_{SNP}=60 SNPs, respectively. Simulated data were analyzed in 5 ways all yielding a gene-based p-value. Sum Gates: original Gates procedure conducted on the univariate sum score calculated across the 20 phenotypes; Sum multiple regression: univariate phenotypic sum score regressed on all SNPs within a gene; MANOVA: all 20 phenotypes as dependent variables and all SNPs within a gene as predictors; GATES-MANOVA: all 20 phenotypes as dependent variables and each SNP separately as predictor in MANOVA, the N_{SNP} MANOVA p-values are then combined using GATES; MGAS: weighted selection of the *mxn* p-values obtained in regression of all *m* phenotypes on all *n* SNPs within a gene, while correcting for the expected correlations between these p-values resulting from the phenotypic correlations and the LD-structure within a gene. MAF was .2 for all simulated SNPs. Within and between LD-blocks, LD measure *r* was set to .9 and to 0, respectively. As the MGAS procedure has less power to detect larger genes (see Supplemental Information), small genes and large genes explained .5% and 1% of the variance, respectively. As a consequence, power results are not directly comparable between small and large genes. When a gene harboured multiple DSL, the total gene effect was equally distributed across all DSL.
* The Roman numerals refer to Figure 2 in the paper. ** Half of the DSL in a gene conveyed a positive effect, the others a negative effect on the factors (Figure 1a,c) or phenotypes (Figure 1c,d,e,f). *** In latent factor models (Figure 1a,c), the one DSL affected half of the phenotypic indicators of a factor positively and the other half negatively. In unclustered networks (Figure 1e), the one DSL affected 1 phenotype positively and another phenotype in the network negatively. In clustered networks (Figure 1f), the one DSL affected either 2 phenotypes in the same cluster, or 2 phenotypes in different clusters, in opposite directions.

Table S5
4 factor model, gene-effect specific to one phenotype

		GATES on sum	GATES on MANOVA	MANOVA	Multiple regression on sum	MGAS
Small gene (10 SNPs; .5% explained)	Scenario 1 *1 block, 0 DSL*	**0.0455**	**0.0515**	**0.0510**	**0.0535**	**0.0430**
	Scenario 2 (IV)[*] *1 block, 1 DSL*	0.0460	0.6365	0.2240	0.0515	0.5025
	Scenario 4 (V) *1 block, 2 DSL*	0.0665	0.9490	0.4695	0.0545	0.8655
	Scenario 5 *1 block, 2 DSL (opp[**])*	0.0540	0.0435	0.0515	0.0595	0.0485
	Scenario 6 (VI) *1 block, 4 DSL*	0.0760	1.0000	0.8820	0.0600	0.9995
	Scenario 7 *1 block, 4 DSL (opp[**])*	0.0375	0.0475	0.0540	0.0515	0.0485
	Scenario 8 (VII) *2 blocks, 1 DSL*	0.0590	0.5700	0.1980	0.0475	0.4495
	Scenario 9 (VIII) *2 blocks, 2 DSL*	0.0650	0.4245	0.2165	0.0545	0.3035
	Scenario 10 (XI) *2 block, 2 DSL (opp[**])*	0.0620	0.4510	0.2110	0.0555	0.3280
Large gene (60 SNPs; 1% explained)	Scenario 11 *8 blocks, 0 DSL*	**0.0570**	**0.0540**	**0.0470**	**0.0495**	**0.0605**
	Scenario 12 (I) *8 blocks, 1 DSL*	0.0620	0.8970	0.1745	0.0565	0.7770
	Scenario 13 (II) *8 blocks, 8 DSL*	0.0510	0.3025	0.1810	0.0645	0.2190
	Scenario 14 (III) *8 blocks, 8 DSL (opp[**])*	0.0610	0.2850	0.1725	0.0480	0.2025

Note. DSL=Disease Susceptibility Loci. All Nrep=2000 simulations included N=2000 subjects, Nphen=20 phenotypes, and small or large genes covering N_{SNP}=10 or N_{SNP}=60 SNPs, respectively. Simulated data were analyzed in 5 ways all yielding a gene-based p-value. Sum Gates: original Gates procedure conducted on the univariate sum score calculated across the 20 phenotypes; Sum multiple regression: univariate phenotypic sum score regressed on all SNPs within a gene; MANOVA: all 20 phenotypes as dependent variables and all SNPs within a gene as predictors; GATES-MANOVA: all 20 phenotypes as dependent variables and each SNP separately as predictor in MANOVA, the N_{SNP} MANOVA p-values are then combined using GATES; MGAS: weighted selection of the *mxn* p-values obtained in regression of all *m* phenotypes on all *n* SNPs within a gene, while correcting for the expected correlations between these p-values resulting from the phenotypic correlations and the LD-structure within a gene. MAF was .2 for all simulated SNPs. Within and between LD-blocks, LD measure *r* was set to .9 and to 0, respectively. As the MGAS procedure has less power to detect larger genes (see Supplemental Information), small genes and large genes explained .5% and 1% of the variance, respectively. As a consequence, power results are not directly comparable between small and large genes. When a gene harboured multiple DSL, the total gene effect was equally distributed across all DSL.
[*] The Roman numerals refer to Figure 2 in the paper. [**] Half of the DSL in a gene conveyed a positive effect, the others a negative effect on the factors (Figure 1a,c) or phenotypes (Figure 1c,d,e,f). [***] In latent factor models (Figure 1a,c), the one DSL affected half of the phenotypic indicators of a factor positively and the other half negatively. In unclustered networks (Figure 1e), the one DSL affected 1 phenotype positively and another phenotype in the network negatively. In clustered networks (Figure 1f), the one DSL affected either 2 phenotypes in the same cluster, or 2 phenotypes in different clusters, in opposite directions.

Table S6
Network model mimicking 1-factor model (i.e., all phenotypes equally strongly related), gene-effect specific to one phenotype

		GATES on sum	GATES on MANOVA	MANOVA	Multiple regression on sum	MGAS
Small gene (10 SNPs; .5% explained)	Scenario 1<br>*1 block, 0 DSL*	**0.0495**	**0.0485**	**0.0465**	**0.0470**	**0.0435**
	Scenario 2 (IV)*<br>*1 block, 1 DSL*	0.1600	0.6855	0.2395	0.0780	0.6680
	Scenario 3 (X)<br>*1 block, 1 DSL (opp***)*	0.0460	0.9880	0.6020	0.0525	0.7865
	Scenario 4 (V)<br>*1 block, 2 DSL*	0.2545	0.9660	0.5280	0.1190	0.9615
	Scenario 5<br>*1 block, 2 DSL (opp**)*	0.0450	0.0485	0.0600	0.0510	0.0460
	Scenario 6 (VI)<br>*1 block, 4 DSL*	0.4345	1.0000	0.9145	0.1945	1.0000
	Scenario 7<br>*1 block, 4 DSL (opp**)*	0.0355	0.0610	0.0705	0.0475	0.0400
	Scenario 8 (VII)<br>*2 blocks, 1 DSL*	0.1340	0.6465	0.2435	0.0875	0.6040
	Scenario 9 (VIII)<br>*2 blocks, 2 DSL*	0.1250	0.4980	0.2610	0.0780	0.4350
	Scenario 10 (IX)<br>*2 block, 2 DSL (opp**)*	0.1050	0.2310	0.1290	0.0655	0.1925
Large gene (60 SNPs; 1% explained)	Scenario 11<br>*8 blocks, 0 DSL*	**0.0550**	**0.0575**	**0.0505**	**0.0470**	**0.0535**
	Scenario 12 (I)<br>*8 blocks, 1 DSL*	0.1080	0.9340	0.2070	0.0640	0.9090
	Scenario 13 (II)<br>*8 blocks, 8 DSL*	0.1085	0.3465	0.1710	0.0670	0.2865
	Scenario 14 (III)<br>*8 blocks, 8 DSL (opp**)*	0.0780	0.1605	0.1075	0.0580	0.1275

Note. DSL=Disease Susceptibility Loci. All Nrep=2000 simulations included N=2000 subjects, Nphen=20 phenotypes, and small or large genes covering N_{SNP}=10 or N_{SNP}=60 SNPs, respectively. Simulated data were analyzed in 5 ways all yielding a gene-based p-value. Sum Gates: original Gates procedure conducted on the univariate sum score calculated across the 20 phenotypes; Sum multiple regression: univariate phenotypic sum score regressed on all SNPs within a gene; MANOVA: all 20 phenotypes as dependent variables and all SNPs within a gene as predictors; GATES-MANOVA: all 20 phenotypes as dependent variables and each SNP separately as predictor in MANOVA, the N_{SNP} MANOVA p-values are then combined using GATES; MGAS: weighted selection of the *mxn* p-values obtained in regression of all *m* phenotypes on all *n* SNPs within a gene, while correcting for the expected correlations between these p-values resulting from the phenotypic correlations and the LD-structure within a gene. MAF was .2 for all simulated SNPs. Within and between LD-blocks, LD measure *r* was set to .9 and to 0, respectively. As the MGAS procedure has less power to detect larger genes (see Supplemental Information), small genes and large genes explained .5% and 1% of the variance, respectively. As a consequence, power results are not directly comparable between small and large genes. When a gene harboured multiple DSL, the total gene effect was equally distributed across all DSL.
* The Roman numerals refer to Figure 2 in the paper. ** Half of the DSL in a gene conveyed a positive effect, the others a negative effect on the factors (Figure 1a,c) or phenotypes (Figure 1c,d,e,f). *** In latent factor models (Figure 1a,c), the one DSL affected half of the phenotypic indicators of a factor positively and the other half negatively. In unclustered networks (Figure 1e), the one DSL affected 1 phenotype positively and another phenotype in the network negatively. In clustered networks (Figure 1f), the one DSL affected either 2 phenotypes in the same cluster, or 2 phenotypes in different clusters, in opposite directions.

Table S7
Network model mimicking 4-factor model (i.e., clusters of phenotypes), gene-effect specific to one phenotype

		GATES on sum	GATES on MANOVA	MANOVA	Multiple regression on sum	MGAS
Small gene (10 SNPs; .5% explained)	Scenario 1<br>*1 block, 0 DSL*	**0.0450**	**0.0545**	**0.0465**	**0.0450**	**0.0470**
	Scenario 2 (IV)[*]<br>*1 block, 1 DSL*	0.1235	0.5815	0.1820	0.0710	0.6805
	Scenario 3 (X)<br>*1 block, 1 DSL (opp[***])*	0.0410	0.9500	0.4800	0.0445	0.6700
	Scenario 3 (XI)<br>*1 block, 1 DSL (opp[***])*	0.0405	0.9555	0.4930	0.0435	0.8960
	Scenario 4 (V)<br>*1 block, 2 DSL*	0.2280	0.9245	0.4370	0.1010	0.9620
	Scenario 5<br>*1 block, 2 DSL (opp[**])*	0.0440	0.0515	0.0640	0.0540	0.0540
	Scenario 6 (VI)<br>*1 block, 4 DSL*	0.4005	1.0000	0.8370	0.1745	1.0000
	Scenario 7<br>*1 block, 4 DSL (opp[**])*	0.0440	0.0470	0.0605	0.0530	0.0470
	Scenario 8 (VII)<br>*2 blocks, 1 DSL*	0.1115	0.5240	0.1845	0.0665	0.6250
	Scenario 9 (VIII)<br>*2 blocks, 2 DSL*	0.1095	0.3990	0.2010	0.0735	0.4445
	Scenario 10 (IX)<br>*2 block, 2 DSL (opp[**])*	0.0850	0.2325	0.1175	0.0685	0.2495
Large gene (60 SNPs; 1% explained)	Scenario 11<br>*8 blocks, 0 DSL*	**0.0555**	**0.0575**	**0.0435**	**0.0500**	**0.0590**[****]
	Scenario 12 (I)<br>*8 blocks, 1 DSL*	0.0985	0.8510	0.1615	0.0680	0.9295
	Scenario 13 (II)<br>*8 blocks, 8 DSL*	0.1065	0.2970	0.1890	0.0760	0.3100
	Scenario 14 (III)<br>*8 blocks, 8 DSL (opp)*	0.0740	0.1620	0.1070	0.0490	0.1580

Note. DSL=Disease Susceptibility Loci. All Nrep=2000 simulations included N=2000 subjects, Nphen=20 phenotypes, and small or large genes covering N_{SNP}=10 or N_{SNP}=60 SNPs, respectively. Simulated data were analyzed in 5 ways all yielding a gene-based p-value. Sum Gates: original Gates procedure conducted on the univariate sum score calculated across the 20 phenotypes; Sum multiple regression: univariate phenotypic sum score regressed on all SNPs within a gene; MANOVA: all 20 phenotypes as dependent variables and all SNPs within a gene as predictors; GATES-MANOVA: all 20 phenotypes as dependent variables and each SNP separately as predictor in MANOVA, the N_{SNP} MANOVA p-values are then combined using GATES; MGAS: weighted selection of the *mxn* p-values obtained in regression of all *m* phenotypes on all *n* SNPs within a gene, while correcting for the expected correlations between these p-values resulting from the phenotypic correlations and the LD-structure within a gene. MAF was .2 for all simulated SNPs. Within and between LD-blocks, LD measure *r* was set to .9 and to 0, respectively. As the MGAS procedure has less power to detect larger genes (see Supplemental Information), small genes and large genes explained .5% and 1% of the variance, respectively. As a consequence, power results are not directly comparable between small and large genes. When a gene harboured multiple DSL, the total gene effect was equally distributed across all DSL.
[*] The Roman numerals refer to Figure 2 in the paper. [**] Half of the DSL in a gene conveyed a positive effect, the others a negative effect on the factors (Figure 1a,c) or phenotypes (Figure 1c,d,e,f). [***] In latent factor models (Figure 1a,c), the one DSL affected half of the phenotypic indicators of a factor positively and the other half negatively. In unclustered networks (Figure 1e), the one DSL affected 1 phenotype positively and another phenotype in the network negatively. In clustered networks (Figure 1f), the one DSL affected either 2 phenotypes in the same cluster (X), or 2 phenotypes in different clusters (XI), in opposite directions. [****]Across multiple simulation runs, this scenario showed a slight tendency towards inflation (~.063).

Table S8
Phenotypic correlations between 9 metabolic traits, corrected for sex, oral contraceptive use and pregnancy

	BMI	CRP	DBP	GLU	HDL	INS	LDL	SBP	TG
BMI	1	.220	.304	.182	-.306	.519	.227	.295	.323
CRP	.220	1	.056	.036	-.092	.154	.000	.071	.092
DBP	.304	.056	1	.132	-.068	.296	.136	.599	.230
GLU	.182	.036	.132	1	-.045	.313	.020	.175	.104
HDL	-.306	-.092	-.068	-.045	1	-.273	-.137	-.013	-.325
INS	.519	.154	.296	.313	-.273	1	.189	.261	.443
LDL	.227	.000	.136	.020	-.137	.189	1	.087	.256
SBP	.295	.071	.599	.175	-.013	.261	.087	1	.177
TG	.323	.092	.230	.104	-.325	.443	.256	.177	1

Note: Abbreviations are: body mass index (BMI), C-reactive protein (CRP), diastolic blood pressure (DBP), glucose (GLU), high-density Lipoprotein (HDL), insulin (INS), low-density lipoprotein (LDL), systolic blood pressure (SBP), and triglycerides (TG). These 9 traits are part of the Northern Finland Birth Cohort data (NFBC1966, Sabatti et al. 2009), found at http://www.ncbi.nlm.nih.gov/gap, controlled through dbGaP study accession number phs000276.v1.p1. The NFBC1966 Study is conducted and supported by the National Heart, Lung, and Blood Institute (NHLBI) in collaboration with the Broad Institute, UCLA, University of Oulu, and the National Institute for Health and Welfare in Finland. This manuscript was not prepared in collaboration with investigators of the NFBC1966 Study and does not necessarily reflect the opinions or views of the NFBC1966 Study Investigators, Broad Institute, UCLA, University of Oulu, National Institute for Health and Welfare in Finland, and the NHLBI.

Sabatti, C., Service, S.K., Hartikainen, A-L, Pouta, A., Ripatti, S., Brodsky, J., et al. (2009). Genome-wide association analysis of metabolic traits in a birth cohort from a founder population. Nat. Genet., 41, 35-46.

TABLE S9: List of FDR corrected genes/genetic regions identified by MGAS in the metabolic data

Color coding p-values:		
	red	<.000001
	yellow	.000001-.00001
	green	.00001-.0001
	blue	.0001-.001
	black font	.001-.05
	grey font	>.05

Symbol	MGAS PVal	IsSignificant	Chrom	Start_Position	Length	SNP	GeneFeature	BMI	CRP	DBP	GLU	HDL	INS	LDL	SBP	TG
CETP	2.64E-27	TRUE	16	56995834	21923	rs5882	exonic	0.03337	0.2277	0.8921	0.7482	0.01234	0.6682	0.9856	0.7947	0.2581
CETP	-	-	-	-	-	rs7499892	intronic	0.05331	0.3716	0.2021	0.05806	2.29E-16	0.6125	0.8722	0.7906	0.6591
CETP	-	-	-	-	-	rs1532624	intronic	0.1196	0.3991	0.719	0.125	2.97E-22	0.08552	0.253	0.8547	0.01902
CETP	-	-	-	-	-	rs3764261	upstream	0.7867	0.2128	0.5447	0.2744	6.97E-29	0.5155	0.155	0.9677	0.07756
CETP	-	-	-	-	-	rs4784744	intronic	0.9047	0.2958	0.3813	0.6869	0.001547	0.01043	0.03085	0.64	0.009365
CRP	4.79E-21	TRUE	1	159682078	2302	rs2794520	downstream	0.5436	2.92E-22	0.5156	0.5865	0.2572	0.3577	0.544	0.2896	0.6739
CRP	-	-	-	-	-	rs2808630	downstream	0.7177	0.1592	0.6079	0.8536	0.4855	0.5051	0.6753	0.726	0.5424
PSRC1	3.66E-11	TRUE	1	109822175	3616	rs646776	downstream	0.1401	0.2212	0.6273	0.8053	0.1416	0.07783	2.19E-12	0.5509	0.5495
PSRC1	-	-	-	-	-	rs14000	3UTR	0.1916	0.09687	0.0892	0.2581	0.6445	0.1435	0.1921	0.5668	0.6507
CELSR2	1.07E-10	TRUE	1	109792640	25739	rs646776	downstream	0.1401	0.2212	0.6273	0.8053	0.1416	0.07783	2.19E-12	0.5509	0.5495
CELSR2	-	-	-	-	-	rs14000	3UTR	0.1916	0.09687	0.0892	0.2581	0.6445	0.1435	0.1921	0.5668	0.6507
CELSR2	-	-	-	-	-	rs4970833	intronic	0.4169	0.4745	0.903	0.1187	0.08689	0.957	1.80E-04	0.1492	0.006173
CELSR2	-	-	-	-	-	rs585362	upstream	0.4312	0.1302	0.9314	0.6189	0.1309	0.5631	7.88E-04	0.9039	0.254
CELSR2	-	-	-	-	-	rs608196	intronic	0.5951	0.02572	0.2761	0.2334	0.6895	0.5521	0.2932	0.824	0.7671
CELSR2	-	-	-	-	-	rs611917	intronic	0.62	0.9362	0.6067	0.2556	0.3181	0.4759	2.49E-07	0.7242	0.4669
CELSR2	-	-	-	-	-	rs437444	exonic	0.9746	0.01237	0.2472	0.07295	0.07532	0.5549	0.2433	0.03432	0.6468
GCKR	9.72E-09	TRUE	2	27719705	26846	rs780090	upstream	0.4083	0.6039	0.077	0.7938	0.9573	0.9804	0.001355	0.9441	0.001157
GCKR	-	-	-	-	-	rs1260326	exonic	0.492	0.07309	0.5136	0.3899	0.1862	0.4225	0.3074	0.3802	3.56E-10
GCKR	-	-	-	-	-	rs780092	intronic	0.7381	0.8739	0.9692	0.4828	0.2053	0.8628	0.605	0.4487	0.002774
GCKR	-	-	-	-	-	rs780094	intronic	0.8774	0.1497	0.3194	0.2858	0.3188	0.2942	0.494	0.1694	0.2486
G6PC2	1.38E-08	TRUE	2	169757749	8762	rs3821117	intronic	0.2223	0.485	0.7182	0.6035	0.4264	0.2869	0.7342	0.1685	0.9942
G6PC2	-	-	-	-	-	rs560887	intronic	0.6882	0.4081	0.9984	5.69E-10	0.7242	0.9469	0.5264	0.9864	0.5293
G6PC2	-	-	-	-	-	rs491443	downstream	0.7258	0.3268	0.4481	0.03655	0.7391	0.9486	0.9368	0.4742	0.668

HNF1A-AS1	2.48E-08	TRUE	12	121407640	2456	rs2254779	ncRNA	0.5124	0.1338	0.2373	0.9861	0.7389	0.9154	0.6181	0.2521	0.9738
HNF1A-AS1	-	-	-	-	-	rs7953249	downstream	0.5548	1.44E-09	0.4425	0.9052	0.4757	0.5462	0.2955	0.6843	0.9386
HNF1A	5.55E-08	TRUE	12	121416548	23767	rs1169302	intronic	0.3462	1.20E-07	0.8893	0.02727	0.1185	0.4196	0.3375	0.1404	0.7278
HNF1A	-	-	-	-	-	rs2464196	exonic	0.5168	4.78E-09	0.6792	0.8519	0.7413	0.2968	0.3052	0.6581	0.7054
HNF1A	-	-	-	-	-	rs1169300	intronic	0.6507	2.01E-09	0.6845	0.8948	0.8545	0.3737	0.1907	0.7302	0.6947
HNF1A	-	-	-	-	-	rs735396	intronic	0.7766	2.13E-07	0.939	0.9274	0.926	0.1533	0.1433	0.9335	0.2894
HNF1A	-	-	-	-	-	rs1169307	intronic	0.8254	2.76E-05	0.7082	0.7806	0.0823	0.7835	0.3745	0.813	0.9292
C12orf43	1.30E-07	TRUE	12	121440224	14082	rs12819210	exonic	0.1806	0.09316	0.1123	0.3683	0.3577	0.5646	0.4985	0.4996	0.4686
C12orf43	-	-	-	-	-	rs2464196	exonic	0.5168	4.78E-09	0.6792	0.8519	0.7413	0.2968	0.3052	0.6581	0.7054
C12orf43	-	-	-	-	-	rs735396	intronic	0.7766	2.13E-07	0.939	0.9274	0.926	0.1533	0.1433	0.9335	0.2894
C12orf43	-	-	-	-	-	rs1169307	intronic	0.8254	2.76E-05	0.7082	0.7806	0.0823	0.7835	0.3745	0.813	0.9292
DPEP3	2.58E-07	TRUE	16	68009565	4888	rs255049	intronic	0.361	0.994	0.9463	0.455	3.06E-08	0.686	0.2529	0.6297	0.13
CTCF	2.79E-07	TRUE	16	67596309	76780	rs6499137	3UTR	0.3175	0.604	0.4746	0.5039	2.20E-08	0.7461	0.9509	0.4758	0.9843
CTCF	-	-	-	-	-	rs7191281	intronic	0.4947	0.8051	0.07599	0.7332	7.72E-06	0.9608	0.4549	0.145	0.7895
NUTF2	3.92E-07	TRUE	16	67880818	24402	rs2271293	intronic	0.897	0.8916	0.8827	0.1609	4.66E-08	0.1624	0.4117	0.5012	0.9857
EDC4	6.16E-07	TRUE	16	67906925	11493	rs2271293	intronic	0.897	0.8916	0.8827	0.1609	4.66E-08	0.1624	0.4117	0.5012	0.9857
EDC4	-	-	-	-	-	rs10468274	upstream	0.937	0.8623	0.322	0.08282	3.56E-07	0.2608	0.3686	0.3434	0.6258
NR1H3	6.21E-07	TRUE	11	47279467	11118	rs2167079	exonic	0.04661	0.02844	0.967	0.5867	0.1763	0.7056	0.1238	0.104	0.07277
NR1H3	-	-	-	-	-	rs7120118	intronic	0.0552	0.0234	0.8924	0.5608	3.57E-08	0.8093	0.1874	0.05825	0.08197
NR1H3	-	-	-	-	-	rs11039149	intronic	0.9772	0.09349	0.3054	0.009321	5.09E-04	0.5538	0.5083	0.1374	0.02265
C16orf86	7.44E-07	TRUE	16	67700716	1946	rs7187476	intronic	0.8978	0.3838	0.2378	0.09445	8.84E-08	0.5415	0.383	0.09347	0.5264
ENKD1	7.44E-07	TRUE	16	67696849	3780	rs7187476	intronic	0.8978	0.3838	0.2378	0.09445	8.84E-08	0.5415	0.383	0.09347	0.5264
FHOD1	8.25E-07	TRUE	16	67263291	18135	rs3852700	intronic	0.1658	0.3847	0.2787	0.2168	9.71E-08	0.6705	0.9209	0.5501	0.394
FHOD1	-	-	-	-	-	rs7196989	intronic	0.1727	0.4299	0.2849	0.2615	1.51E-07	0.7042	0.8385	0.5127	0.4318
APOB	1.13E-06	TRUE	2	21224300	42646	rs1713222	upstream	0.2007	0.9986	0.09599	0.9239	0.4318	0.197	9.33E-08	0.07737	0.842
APOB	-	-	-	-	-	rs673548	intronic	0.3226	0.1194	0.9773	0.09501	3.40E-06	0.8316	2.28E-07	0.4084	2.01E-08
APOB	-	-	-	-	-	rs676210	exonic	0.3302	0.115	0.9899	0.1007	4.78E-06	0.8192	2.60E-07	0.4213	3.36E-08
APOB	-	-	-	-	-	rs1801701	exonic	0.4387	0.05612	0.3321	0.7412	0.5346	0.626	0.01127	0.009603	0.3052
APOB	-	-	-	-	-	rs679899	exonic	0.5144	0.9024	0.2513	0.8477	0.02394	0.3973	0.9247	0.1702	0.1086
APOB	-	-	-	-	-	rs550619	intronic	0.5415	0.6963	0.7224	0.8093	0.2593	0.7013	0.06421	0.8872	0.7822
APOB	-	-	-	-	-	rs1042031	exonic	0.7497	0.9532	0.03983	0.5051	0.3252	0.2155	0.2249	0.3996	0.05475
APOB	-	-	-	-	-	rs693	exonic	0.7777	0.7209	0.1151	0.4707	0.4173	0.6898	0.4354	0.7779	0.7897
TMEM208	1.27E-06	TRUE	16	67261015	2168	rs7196989	intronic	0.1727	0.4299	0.2849	0.2615	1.51E-07	0.7042	0.8385	0.5127	0.4318

LRRC29	1.27E-06	TRUE	16	67241041	19681	rs7196989	intronic	0.1727	0.4299	0.2849	0.2615	1.51E-07	0.7042	0.8385	0.5127	0.4318
EXOC3L1	1.38E-06	TRUE	16	67218281	5827	rs868213	intronic	0.1796	0.399	0.3079	0.2276	1.64E-07	0.6852	0.8226	0.5289	0.4248
PARD6A	1.42E-06	TRUE	16	67694850	1832	rs6979	exonic	0.7718	0.6939	0.7108	0.08113	0.0283	0.5256	0.2619	0.4855	0.1681
PARD6A	-	-	-	-	-	rs7187476	intronic	0.8978	0.3838	0.2378	0.09445	8.84E-08	0.5415	0.383	0.09347	0.5264
NOL3	1.45E-06	TRUE	16	67204399	5242	rs2233455	exonic	0.134	0.4108	0.1971	0.2326	1.72E-07	0.5636	0.9906	0.5565	0.4772
HSF4	1.45E-06	TRUE	16	67197287	6562	rs2233455	exonic	0.134	0.4108	0.1971	0.2326	1.72E-07	0.5636	0.9906	0.5565	0.4772
TOMM40	1.47E-06	TRUE	19	45394476	12471	rs405509	upstream	0.2693	0.7263	0.564	0.2019	0.4985	0.9087	0.668	0.4623	0.6398
TOMM40	-	-	-	-	-	rs8106922	intronic	0.7305	6.64E-05	0.4315	0.3706	0.03617	0.9033	0.2162	0.7173	0.8568
TOMM40	-	-	-	-	-	rs157580	intronic	0.8084	0.3781	0.2169	0.3004	0.001642	0.7381	4.96E-08	0.8885	8.61E-04
TOMM40	-	-	-	-	-	rs2075650	intronic	0.9345	0.004446	0.3169	0.7306	0.1825	0.4913	1.05E-05	0.4625	1.81E-05
KIAA0895L	1.48E-06	TRUE	16	67209504	8380	rs2233455	exonic	0.134	0.4108	0.1971	0.2326	1.72E-07	0.5636	0.9906	0.5565	0.4772
KIAA0895L	-	-	-	-	-	rs868213	intronic	0.1796	0.399	0.3079	0.2276	1.64E-07	0.6852	0.8226	0.5289	0.4248
PRMT7	1.49E-06	TRUE	16	68344876	46294	rs3743742	intronic	0.274	0.4489	0.5809	0.3548	0.01252	0.6735	0.4857	0.5307	0.3085
PRMT7	-	-	-	-	-	rs3803650	exonic	0.2847	0.2419	0.7827	0.6362	0.01556	0.7769	0.891	0.7625	0.2056
PRMT7	-	-	-	-	-	rs1111571	intronic	0.3669	0.2148	0.8728	0.3225	0.03644	0.7666	0.5186	0.6334	0.8972
PRMT7	-	-	-	-	-	rs3785123	intronic	0.3996	0.6257	0.5531	0.771	0.07176	0.8639	0.7268	0.4931	0.7163
PRMT7	-	-	-	-	-	rs3961282	intronic	0.4055	0.1729	0.7639	0.3206	0.04415	0.8176	0.5158	0.5594	0.9506
PRMT7	-	-	-	-	-	rs4783554	intronic	0.4801	0.1867	0.9942	0.5747	0.02467	0.9468	0.5136	0.5837	0.859
PRMT7	-	-	-	-	-	rs8058517	intronic	0.9079	0.2653	0.6002	0.4823	4.37E-08	0.8073	0.9179	0.7823	0.3082
MADD	1.50E-06	TRUE	11	47291198	60385	rs326217	exonic	0.01679	0.4316	0.6365	0.02206	0.07119	0.3848	0.2229	0.2298	0.9868
MADD	-	-	-	-	-	rs7120118	intronic	0.0552	0.0234	0.8924	0.5608	3.57E-08	0.8093	0.1874	0.05825	0.08197
MADD	-	-	-	-	-	rs2290149	intronic	0.1168	0.01191	0.3215	0.7586	0.009796	0.09805	0.00883	0.3908	0.1096
MADD	-	-	-	-	-	rs4752979	intronic	0.133	0.3565	0.7863	0.9002	1.49E-05	0.5598	0.841	0.2174	0.3536
MADD	-	-	-	-	-	rs4752977	intronic	0.2809	0.4936	0.6932	0.452	9.72E-06	0.4263	0.4551	0.3559	0.3833
MADD	-	-	-	-	-	rs1051006	exonic	0.2949	0.4965	0.677	0.4561	0.3127	0.418	0.4624	0.3432	0.3799
MADD	-	-	-	-	-	rs749067	intronic	0.9067	0.1021	0.6539	0.004536	3.01E-05	0.552	0.8723	0.4897	0.06264
PLA2G15	1.65E-06	TRUE	16	68279246	15716	rs1975802	intronic	0.4813	0.8637	0.557	0.7194	1.95E-07	0.8525	0.2248	0.6508	0.2139
DPEP2	1.98E-06	TRUE	16	68021292	12130	rs255052	intronic	0.4782	0.8888	0.6575	0.6429	2.36E-07	0.669	0.2896	0.4469	0.3692
NRN1L	3.00E-06	TRUE	16	67918780	1492	rs10468274	upstream	0.937	0.8623	0.322	0.08282	3.56E-07	0.2608	0.3686	0.3434	0.6258
PVRL2	3.04E-06	TRUE	19	45349392	32807	rs1871047	intronic	0.1248	0.3252	0.9286	0.02019	0.08085	0.3568	0.7368	0.2255	0.08093
PVRL2	-	-	-	-	-	rs377702	intronic	0.1839	0.6437	0.326	0.5283	0.7807	0.8546	0.2287	0.3657	0.433
PVRL2	-	-	-	-	-	rs4803766	intronic	0.2928	0.55	0.9738	0.883	0.9175	0.1381	0.716	0.07416	0.09686
PVRL2	-	-	-	-	-	rs6859	3UTR	0.2988	0.1659	0.5211	0.03266	0.05445	0.4336	0.01207	0.4281	0.6264

PVRL2	-	-	-	-	-	rs2075642	intronic	0.4624	0.1421	0.3262	0.3212	0.4169	0.454	0.07954	0.4881	0.08497
PVRL2	-	-	-	-	-	rs11667640	intronic	0.4703	0.7964	0.3047	0.8687	0.9642	0.8738	0.7322	0.5229	0.7691
PVRL2	-	-	-	-	-	rs157580	intronic	0.8084	0.3781	0.2169	0.3004	0.001642	0.7381	4.96E-08	0.8885	8.61E-04
PVRL2	-	-	-	-	-	rs2075650	intronic	0.9345	0.004446	0.3169	0.7306	0.1825	0.4913	1.05E-05	0.4625	1.81E-05
PSKH1	6.07E-06	TRUE	16	67927174	36408	rs12599580	intronic	0.9314	0.5048	0.3158	0.7402	0.8556	0.4018	0.6161	0.1704	0.563
PSKH1	-	-	-	-	-	rs10468274	upstream	0.937	0.8623	0.322	0.08282	3.56E-07	0.2608	0.3686	0.3434	0.6258
CTCFL	7.96E-06	TRUE	20	56093554	6630	rs2902991	downstream	0.1626	0.1221	0.5357	0.1721	0.6693	0.7378	0.05563	0.4624	0.9749
CTCFL	-	-	-	-	-	rs2865387	downstream	0.3611	0.5227	0.9577	0.1151	0.8907	0.9392	0.1316	0.8262	0.7117
CTCFL	-	-	-	-	-	rs11700100	intronic	0.4982	0.9208	0.9785	0.667	1.61E-07	0.2883	0.457	0.9458	0.5406
CTCFL	-	-	-	-	-	rs6025606	exonic	0.5986	0.3143	0.9189	0.7903	0.7312	0.7929	0.3923	0.7978	0.3028
CTCFL	-	-	-	-	-	rs12625924	intronic	0.6118	0.1517	0.1306	0.8342	0.9064	0.5792	0.1079	0.3213	0.8778
CTCFL	-	-	-	-	-	rs12106016	upstream	0.8154	0.0653	0.1938	0.1883	0.3119	0.7773	0.6438	0.7417	0.4654
CTCFL	-	-	-	-	-	rs6025590	downstream	0.9559	0.6856	0.9684	0.6225	0.6592	0.9411	0.04896	0.7404	0.977
AR	8.49E-06	TRUE	X	66788682	161780	rs5031002	intronic	0.1203	0.5493	0.8535	0.5407	0.6375	0.03562	2.37E-07	0.7995	0.09491
AR	-	-	-	-	-	rs12014709	intronic	0.4449	0.2966	0.4357	0.387	0.4634	0.01782	0.7424	0.354	0.3188
AR	-	-	-	-	-	rs1337080	intronic	0.6287	0.524	0.9748	0.901	0.7529	0.2924	0.874	0.9389	0.6503
AR	-	-	-	-	-	rs4827545	intronic	0.8437	0.8471	0.4232	0.6265	0.4827	0.01961	0.8713	0.4208	0.296
AR	-	-	-	-	-	rs5918760	intronic	0.9207	0.7999	0.5648	0.4695	0.4571	0.01636	0.8925	0.5034	0.2908
AR	-	-	-	-	-	rs5918762	intronic	0.9219	0.7404	0.6815	0.5977	0.4623	0.01802	0.8591	0.4948	0.3132
AR	-	-	-	-	-	rs12396249	intronic	0.9392	0.7706	0.5242	0.5048	0.4954	0.01812	0.9013	0.4516	0.3194
AR	-	-	-	-	-	rs2255702	intronic	0.9445	0.807	0.5542	0.5187	0.4938	0.02112	0.8691	0.4871	0.3884
AR	-	-	-	-	-	rs5919393	intronic	0.9482	0.8125	0.5946	0.5814	0.4862	0.01892	0.9775	0.4908	0.4019
AR	-	-	-	-	-	rs5918757	intronic	0.9741	0.7796	0.5218	0.5068	0.501	0.01825	0.8936	0.4612	0.3196
NFATC3	1.29E-05	TRUE	16	68119268	143895	rs7190307	intronic	0.07625	0.7489	0.5496	0.8538	0.05021	0.2247	0.5814	0.7409	0.807
NFATC3	-	-	-	-	-	rs2418736	intronic	0.2063	0.5242	0.6792	0.2401	8.20E-07	0.3154	0.1946	0.9375	0.2773
NFATC3	-	-	-	-	-	rs4359427	intronic	0.2127	0.896	0.5135	0.1745	8.21E-07	0.3812	0.3413	0.5929	0.4306
NFATC3	-	-	-	-	-	rs9932251	intronic	0.2187	0.8761	0.5749	0.1691	7.49E-07	0.3608	0.3065	0.6827	0.4313
NFATC3	-	-	-	-	-	rs12598	exonic	0.3372	0.4047	0.5357	0.6799	0.6527	0.1984	0.3843	0.314	0.6151
NFATC3	-	-	-	-	-	rs7193701	intronic	0.4671	0.9677	0.54	0.7222	3.87E-07	0.8884	0.2406	0.7283	0.1849
CR1L	1.44E-05	TRUE	1	207818457	78580	rs4844614	intronic	3.93E-04	0.6738	0.05741	0.2787	0.06369	0.07123	2.38E-07	0.2643	0.003801
CR1L	-	-	-	-	-	rs10494884	intronic	0.03029	0.2476	0.6795	0.3309	0.1463	0.3309	0.004375	0.1447	0.1018
CR1L	-	-	-	-	-	rs10779339	3UTR	0.03386	0.4428	0.6228	0.3817	0.1414	0.4658	0.00726	0.2465	0.08688
CR1L	-	-	-	-	-	rs12080578	intronic	0.04034	0.1241	0.6667	0.01845	0.008374	0.3558	0.005604	0.899	0.002726

CR1L	-	-	-	-	-	rs11118387	intronic	0.06218	0.5264	0.6856	0.3614	0.2198	0.3516	0.006691	0.2118	0.1064
CR1L	-	-	-	-	-	rs2761424	intronic	0.1591	0.922	0.4849	0.8078	0.04714	0.8984	0.783	0.03793	0.2898
CR1L	-	-	-	-	-	rs10863461	intronic	0.2915	0.7668	0.6063	0.8308	0.8778	0.7623	0.122	0.1774	0.9948
CR1L	-	-	-	-	-	rs6696840	intronic	0.3303	0.8839	0.6872	0.7391	0.9048	0.7815	0.1487	0.2026	0.719
CR1L	-	-	-	-	-	rs1998539	intronic	0.8557	0.5431	0.6009	0.872	0.725	0.1092	0.7893	0.02516	0.4884
CR1L	-	-	-	-	-	rs11118475	intronic	0.8954	0.8329	0.5601	0.9211	0.8348	0.2582	0.6501	0.0394	0.6854
OASL	1.44E-05	TRUE	12	121458094	18952	rs10774580	intronic	0.09104	4.97E-07	0.1349	0.6109	0.3377	0.3766	0.9154	0.04601	0.7461
OASL	-	-	-	-	-	rs12819210	exonic	0.1806	0.09316	0.1123	0.3683	0.3577	0.5646	0.4985	0.4996	0.4686
OASL	-	-	-	-	-	rs7965349	intronic	0.2528	9.29E-04	0.3498	0.5059	0.2891	0.4456	0.09535	0.4228	0.2227
OASL	-	-	-	-	-	rs2859394	upstream	0.7846	0.06544	0.4525	0.6604	0.8046	0.1936	0.2813	0.6025	0.3758
S1PR2	1.47E-05	TRUE	19	10332108	9841	rs2288937	intronic	0.6039	0.2611	0.2739	0.02735	0.6087	0.391	0.009269	0.2712	0.09111
S1PR2	-	-	-	-	-	rs2116941	3UTR	0.6523	0.6345	0.3823	0.3754	0.7905	0.3436	0.08409	0.5394	0.2908
S1PR2	-	-	-	-	-	rs10409243	3UTR	0.9912	0.3927	0.06374	0.5824	0.2796	0.6106	6.23E-07	0.2241	0.04488
TFAP2B	1.81E-05	TRUE	6	50786438	28889	rs987237	intronic	7.37E-07	4.17E-04	0.1634	0.2221	0.01615	0.01493	0.3143	0.02494	0.003847
TFAP2B	-	-	-	-	-	rs6930924	intronic	0.2443	0.2082	0.5596	0.491	0.4341	0.05227	0.1029	0.6772	0.1102
TFAP2B	-	-	-	-	-	rs2143081	upstream	0.2492	0.1208	0.9772	0.4722	0.0955	0.252	0.4908	0.9793	0.6482
MIR1908	3.40E-05	TRUE	11	61582632	81	rs174556	intronic	0.798	0.5286	0.9724	0.1236	0.1947	0.2193	4.04E-06	0.5532	0.0737
FADS1	4.46E-05	TRUE	11	61567096	17434	rs174546	3UTR	0.4424	0.7667	0.8104	0.1885	0.0566	0.3892	6.38E-06	0.4388	0.01346
FADS1	-	-	-	-	-	rs174556	intronic	0.798	0.5286	0.9724	0.1236	0.1947	0.2193	4.04E-06	0.5532	0.0737
SMEK2	5.67E-05	TRUE	2	55774427	70434	rs2627763	intronic	0.01983	0.6394	0.00307	0.3014	0.6473	0.3315	0.2034	0.1507	0.7042
SMEK2	-	-	-	-	-	rs782588	intronic	0.1469	0.008509	0.00147	0.0995	0.3175	0.5432	0.8834	1.23E-06	0.6248
SMEK2	-	-	-	-	-	rs782586	intronic	0.168	0.007237	0.001385	0.08265	0.3724	0.4546	0.8069	1.20E-06	0.6219
SMEK2	-	-	-	-	-	rs782602	upstream	0.1705	0.2558	0.001049	0.1916	0.9432	0.4937	0.6655	8.02E-07	0.6299
SMEK2	-	-	-	-	-	rs2291336	upstream	0.1726	0.2719	0.2915	0.05676	0.5375	0.5128	0.9997	1.21E-04	0.7705
SMEK2	-	-	-	-	-	rs10496050	intronic	0.3345	0.006622	0.003577	0.1598	0.1765	0.4943	0.6935	1.28E-05	0.8011
SMEK2	-	-	-	-	-	rs3748945	3UTR	0.339	0.2775	0.02443	0.9764	0.9899	0.9396	0.6134	0.1259	0.6494
SMEK2	-	-	-	-	-	rs10427222	intronic	0.381	0.03238	0.008004	0.651	0.29	0.8509	0.5896	0.07188	0.9988
SMEK2	-	-	-	-	-	rs6737995	intronic	0.4262	0.03441	0.4462	0.1047	0.6194	0.3782	0.2829	1.00E-03	0.9198
SMEK2	-	-	-	-	-	rs12993624	intronic	0.6212	0.1743	0.3093	0.02992	0.4416	0.3897	0.2676	3.08E-04	0.8914
SMEK2	-	-	-	-	-	rs13010217	intronic	0.6874	0.6947	0.3261	0.04681	0.183	0.346	0.6662	5.40E-04	0.6939
SMEK2	-	-	-	-	-	rs10496047	intronic	0.8104	0.2121	0.4451	0.6791	0.8864	0.6734	0.888	0.9184	0.7099
SMEK2	-	-	-	-	-	rs6545508	intronic	0.8259	0.002266	0.3053	0.3065	0.9521	0.6174	0.1362	0.06501	0.2794
SMEK2	-	-	-	-	-	rs10496048	intronic	0.8853	0.005556	0.6735	0.5537	0.2977	0.8406	0.1577	0.3762	0.5423

LEPR	5.96E-05	TRUE	1	65991371	109741	rs3806318	upstream	0.07325	0.2579	0.4602	0.416	0.107	0.1215	0.9253	0.8748	0.06341
LEPR	-	-	-	-	-	rs3828034	intronic	0.1145	0.01439	0.7584	0.286	0.9196	0.5411	0.6851	0.7409	0.3264
LEPR	-	-	-	-	-	rs1022981	intronic	0.15	0.01186	0.3157	0.6603	0.2159	0.3539	0.5367	0.04135	0.1409
LEPR	-	-	-	-	-	rs1782754	intronic	0.1585	0.01166	0.2606	0.7189	0.2462	0.3611	0.4894	0.03281	0.1415
LEPR	-	-	-	-	-	rs1171269	intronic	0.1605	0.012	0.277	0.6478	0.2379	0.3666	0.5619	0.03656	0.1684
LEPR	-	-	-	-	-	rs4655537	intronic	0.193	0.003228	0.2469	0.28	0.731	0.3657	0.9505	0.009924	0.8233
LEPR	-	-	-	-	-	rs10493380	intronic	0.1954	0.009921	0.9301	0.481	0.8365	0.7819	0.4324	0.9011	0.3406
LEPR	-	-	-	-	-	rs1137100	exonic	0.2482	0.005778	0.2761	0.9265	0.155	0.447	0.7622	0.04746	0.1424
LEPR	-	-	-	-	-	rs2025805	intronic	0.2885	0.001495	0.2606	0.9794	0.6511	0.6925	0.5844	0.1556	0.3145
LEPR	-	-	-	-	-	rs970467	intronic	0.3452	0.7653	0.3539	0.8281	0.7636	0.9383	0.9685	0.1961	0.7154
LEPR	-	-	-	-	-	rs9436297	intronic	0.3765	0.2146	0.7122	0.3677	0.8321	0.8844	0.5789	0.4112	0.4792
LEPR	-	-	-	-	-	rs12409877	intronic	0.4008	0.003603	0.09787	0.6312	0.5767	0.949	0.7449	0.01797	0.8755
LEPR	-	-	-	-	-	rs3790426	intronic	0.4457	0.135	0.8522	0.4159	0.06904	0.5273	0.2814	0.8373	0.6003
LEPR	-	-	-	-	-	rs6588147	intronic	0.5311	0.004121	0.007698	0.9679	0.5587	0.8864	0.9255	0.003467	0.8901
LEPR	-	-	-	-	-	rs6673324	intronic	0.6033	0.1814	0.3964	0.5984	0.7639	0.7843	0.3339	0.112	0.3272
LEPR	-	-	-	-	-	rs1171279	intronic	0.7402	0.46	0.2002	0.3526	0.5992	0.7722	0.2958	0.2528	0.531
LEPR	-	-	-	-	-	rs3790433	intronic	0.799	0.6308	0.08339	0.4571	0.4888	0.8525	0.4068	0.08519	0.213
LEPR	-	-	-	-	-	rs2154380	intronic	0.8103	0.272	0.1032	0.7733	0.9332	0.6799	0.6875	0.009193	0.8372
LEPR	-	-	-	-	-	rs1892534	downstream	0.8243	4.77E-07	0.497	0.3704	0.2597	0.7034	0.3746	0.3003	0.9436
LEPR	-	-	-	-	-	rs10158579	intronic	0.8359	0.7728	0.2495	0.3705	0.8163	0.6613	0.8011	0.05447	0.1116
LEPR	-	-	-	-	-	rs1137101	exonic	0.8748	0.2595	0.1106	0.7626	0.8924	0.7602	0.7227	0.008566	0.7423
LEPR	-	-	-	-	-	rs1171278	intronic	1	0.9624	0.8095	0.9637	0.8363	0.8687	0.9845	0.2978	0.0888
GLT8D1	6.59E-05	TRUE	3	52728499	11601	rs6976	3UTR	0.9028	0.1491	0.2448	6.47E-06	0.8249	0.2806	0.5525	0.4594	0.7002
GLT8D1	-	-	-	-	-	rs6762813	intronic	0.9693	0.8074	0.8957	0.004707	0.4603	0.5976	1.30E-04	0.5094	0.6592
GLT8D1	-	-	-	-	-	rs2289247	exonic	0.9737	0.9694	0.8093	0.007429	0.4166	0.5671	1.75E-04	0.5575	0.6664
SNORD69	6.59E-05	TRUE	3	52726751	78	rs6976	3UTR	0.9028	0.1491	0.2448	6.47E-06	0.8249	0.2806	0.5525	0.4594	0.7002
SNORD69	-	-	-	-	-	rs6762813	intronic	0.9693	0.8074	0.8957	0.004707	0.4603	0.5976	1.30E-04	0.5094	0.6592
SNORD69	-	-	-	-	-	rs2289247	exonic	0.9737	0.9694	0.8093	0.007429	0.4166	0.5671	1.75E-04	0.5575	0.6664
FAM222B	7.07E-05	TRUE	17	27082995	86863	rs11658900	intronic	0.03707	0.8323	0.3279	0.3161	0.05639	0.1982	4.54E-04	0.9439	0.02269
FAM222B	-	-	-	-	-	rs4795457	intronic	0.04174	0.01954	0.2237	0.1416	0.6815	0.6314	3.28E-06	0.005963	0.2035
FAM222B	-	-	-	-	-	rs9303621	intronic	0.04495	0.02207	0.2432	0.1502	0.6754	0.6313	2.84E-06	0.006871	0.2239
FAM222B	-	-	-	-	-	rs2043031	exonic	0.04904	0.01705	0.2421	0.1279	0.7106	0.6429	3.65E-06	0.006623	0.2206
FAM222B	-	-	-	-	-	rs4795461	intronic	0.3649	0.4691	0.8632	0.5642	0.1563	0.9859	0.8111	0.06621	0.6475

TBL2	8.31E-05	TRUE	7	72983276	9738	rs11974409	intronic	0.383	0.5928	0.8293	0.7701	0.08545	0.8739	0.6497	0.9588	4.38E-05
TBL2	-	-	-	-	-	rs2286276	intronic	0.5406	0.6007	0.9271	0.8852	0.02891	0.8377	0.9061	0.8319	6.55E-06
HNF4A	8.73E-05	TRUE	20	42984440	77046	rs2071200	intronic	0.005551	0.5327	0.2163	0.01834	0.06294	0.1389	0.0401	0.8199	0.05181
HNF4A	-	-	-	-	-	rs1800961	exonic	0.03336	0.2932	0.05682	0.6639	7.13E-07	0.01698	0.4652	0.4107	0.001891
HNF4A	-	-	-	-	-	rs717248	intronic	0.04513	0.2514	0.9405	0.1686	0.2258	0.1336	0.2832	0.8928	0.8678
HNF4A	-	-	-	-	-	rs8116574	intronic	0.05253	0.3948	0.1104	0.2546	0.3511	0.615	0.2683	0.5441	0.05949
HNF4A	-	-	-	-	-	rs11699172	upstream	0.06202	0.03885	0.6158	0.1886	0.07262	0.9319	0.02679	0.9985	0.02407
HNF4A	-	-	-	-	-	rs6031551	intronic	0.0694	0.9377	0.2964	0.4522	0.2477	0.3484	0.02853	0.08943	0.1331
HNF4A	-	-	-	-	-	rs6073418	intronic	0.09419	0.1778	0.3375	0.2711	0.9305	0.1645	0.138	0.864	0.5573
HNF4A	-	-	-	-	-	rs1885088	intronic	0.1992	0.1949	0.09095	0.9582	0.2952	0.189	0.01963	0.06757	0.4486
HNF4A	-	-	-	-	-	rs2425637	intronic	0.2946	0.3934	0.4661	0.001837	0.4616	0.5899	0.6927	0.4439	0.9631
HNF4A	-	-	-	-	-	rs6031544	upstream	0.3423	0.09723	0.7847	0.6922	0.833	0.8939	0.06637	0.7452	0.4261
HNF4A	-	-	-	-	-	rs6065725	intronic	0.487	0.6857	0.7932	0.3122	0.8649	0.4849	0.02582	0.3384	0.1081
HNF4A	-	-	-	-	-	rs2425640	intronic	0.743	0.5588	0.205	0.01648	0.3673	0.9115	0.6695	0.2458	0.632
HNF4A	-	-	-	-	-	rs4812829	intronic	0.7936	0.6283	0.9882	0.6729	0.2641	0.827	0.7449	0.7068	0.4461
HNF4A	-	-	-	-	-	rs6031580	intronic	0.8758	0.091	0.3912	0.6703	0.8323	0.4151	0.1766	0.1978	0.4449
HNF4A	-	-	-	-	-	rs745975	intronic	0.876	0.9632	0.1737	0.5846	0.09742	0.6801	0.4243	0.3632	0.1761
HNF4A	-	-	-	-	-	rs3212198	intronic	0.984	0.9016	0.1109	0.7824	0.1608	0.2614	0.1155	0.5909	0.2928
IGSF5	9.72E-05	TRUE	21	41117333	56691	rs2837175	intronic	0.04492	0.8799	0.03859	0.5625	0.4003	0.5885	9.66E-07	0.3369	0.02294
IGSF5	-	-	-	-	-	rs2837225	exonic	0.05096	0.5948	0.1286	0.1747	0.4486	0.7524	2.14E-04	0.4958	0.06842
IGSF5	-	-	-	-	-	rs2837226	intronic	0.06676	0.8451	0.05637	0.5041	0.7182	0.45	1.04E-04	0.3191	0.1309
IGSF5	-	-	-	-	-	rs2837227	intronic	0.06955	0.8429	0.0642	0.4316	0.6895	0.5076	8.46E-05	0.3334	0.09417
IGSF5	-	-	-	-	-	rs2837220	intronic	0.1474	0.5475	0.2057	0.3968	0.105	0.4448	4.84E-04	0.6376	0.2266
IGSF5	-	-	-	-	-	rs2253748	intronic	0.1966	0.3183	0.08838	0.3157	0.6532	0.5561	0.09194	0.354	0.8125
IGSF5	-	-	-	-	-	rs2837173	intronic	0.1966	0.3035	0.09544	0.3238	0.628	0.535	0.08394	0.3725	0.7932
IGSF5	-	-	-	-	-	rs456800	intronic	0.2313	0.5927	0.6669	0.7547	0.6906	0.4299	0.1767	0.6668	0.03802
IGSF5	-	-	-	-	-	rs2837170	intronic	0.2381	0.3486	0.1061	0.3309	0.6316	0.5138	0.09686	0.421	0.7618
IGSF5	-	-	-	-	-	rs2837214	intronic	0.2554	0.4915	0.03801	0.2558	0.4099	0.9588	0.552	0.05737	0.6184
IGSF5	-	-	-	-	-	rs744568	intronic	0.2667	0.1647	0.1068	0.2341	0.6417	0.7031	0.0501	0.3376	0.9757
IGSF5	-	-	-	-	-	rs741867	intronic	0.2852	0.2055	0.08214	0.2428	0.6466	0.7005	0.04886	0.2768	0.9787
IGSF5	-	-	-	-	-	rs2837216	intronic	0.3126	0.4709	0.09146	0.2271	0.2666	0.6155	0.2812	0.06754	0.5707
IGSF5	-	-	-	-	-	rs2837208	intronic	0.3154	0.3674	0.03719	0.3528	0.4555	0.9521	0.5383	0.09089	0.7251
IGSF5	-	-	-	-	-	rs2837196	intronic	0.3701	0.6148	0.5055	0.5559	0.3394	0.5322	0.07715	0.8609	0.1408

IGSF5	-	-	-	-	-	rs2837205	intronic	0.3771	0.2085	0.8399	0.7099	0.2319	0.5102	5.90E-04	0.5952	0.03318
IGSF5	-	-	-	-	-	rs2837212	intronic	0.4543	0.3952	0.0844	0.3063	0.2766	0.7595	0.27	0.132	0.6345
IGSF5	-	-	-	-	-	rs463903	intronic	0.5952	0.6386	0.1338	0.2226	0.366	0.8711	0.4388	0.1028	0.4966
IGSF5	-	-	-	-	-	rs464201	intronic	0.6113	0.7863	0.1131	0.2421	0.2977	0.836	0.5067	0.1333	0.4917
IGSF5	-	-	-	-	-	rs460549	downstream	0.6227	0.8114	0.1063	0.2315	0.3193	0.8166	0.5143	0.1277	0.4863
IGSF5	-	-	-	-	-	rs2837167	intronic	0.7165	0.3977	0.9722	0.8928	0.9684	0.625	0.2877	0.6817	0.5145
IGSF5	-	-	-	-	-	rs2837156	intronic	0.8051	0.9101	0.7028	0.9212	0.1321	0.9414	0.001538	0.7718	0.05984
IGSF5	-	-	-	-	-	rs1888493	intronic	0.8214	0.372	0.1662	0.1652	0.3516	0.5694	0.2527	0.7308	0.2373
IGSF5	-	-	-	-	-	rs2837210	intronic	0.8364	0.6152	0.4277	0.8908	0.08227	0.6878	6.88E-04	0.4389	0.09776
GNL3	1.09E-04	TRUE	3	52719935	8576	rs1108842	5UTR	0.5703	0.8264	0.5703	0.02842	0.1382	0.2629	0.006992	0.8594	0.1843
GNL3	-	-	-	-	-	rs11177	exonic	0.8982	0.7372	0.8531	0.73	0.3026	0.6106	0.9126	0.5312	0.7179
GNL3	-	-	-	-	-	rs6976	3UTR	0.9028	0.1491	0.2448	6.47E-06	0.8249	0.2806	0.5525	0.4594	0.7002
GNL3	-	-	-	-	-	rs6762813	intronic	0.9693	0.8074	0.8957	0.004707	0.4603	0.5976	1.30E-04	0.5094	0.6592
GNL3	-	-	-	-	-	rs2289247	exonic	0.9737	0.9694	0.8093	0.007429	0.4166	0.5671	1.75E-04	0.5575	0.6664
SNORD19B	1.09E-04	TRUE	3	52724753	94	rs1108842	5UTR	0.5703	0.8264	0.5703	0.02842	0.1382	0.2629	0.006992	0.8594	0.1843
SNORD19B	-	-	-	-	-	rs11177	exonic	0.8982	0.7372	0.8531	0.73	0.3026	0.6106	0.9126	0.5312	0.7179
SNORD19B	-	-	-	-	-	rs6976	3UTR	0.9028	0.1491	0.2448	6.47E-06	0.8249	0.2806	0.5525	0.4594	0.7002
SNORD19B	-	-	-	-	-	rs6762813	intronic	0.9693	0.8074	0.8957	0.004707	0.4603	0.5976	1.30E-04	0.5094	0.6592
SNORD19B	-	-	-	-	-	rs2289247	exonic	0.9737	0.9694	0.8093	0.007429	0.4166	0.5671	1.75E-04	0.5575	0.6664
ATP13A5	1.15E-04	TRUE	3	192992830	103685	rs12493236	intronic	0.009704	0.2573	0.1898	0.5439	0.07538	3.14E-04	0.3205	0.3087	1.14E-06
ATP13A5	-	-	-	-	-	rs7641152	intronic	0.01358	0.7237	0.4036	0.2495	0.1057	0.04978	0.6289	0.8657	0.06034
ATP13A5	-	-	-	-	-	rs2367477	intronic	0.04822	0.212	0.03582	0.4418	0.9787	0.004509	0.364	0.2706	0.03756
ATP13A5	-	-	-	-	-	rs13085410	intronic	0.05555	0.7833	0.1512	0.9553	0.2548	0.1429	0.07892	0.8086	0.1735
ATP13A5	-	-	-	-	-	rs4687399	intronic	0.05935	0.2272	0.03316	0.4455	0.9549	0.00438	0.3383	0.2531	0.03459
ATP13A5	-	-	-	-	-	rs4481103	intronic	0.06466	0.8139	0.9216	0.9117	0.292	0.236	0.6522	0.5227	0.5934
ATP13A5	-	-	-	-	-	rs2271791	exonic	0.07485	0.3084	0.06598	0.6734	0.8574	0.01544	0.6978	0.325	0.0539
ATP13A5	-	-	-	-	-	rs9851984	intronic	0.1112	0.3354	0.114	0.5505	0.5906	0.2597	0.8173	0.701	0.8251
ATP13A5	-	-	-	-	-	rs1980263	intronic	0.163	0.3019	0.9926	0.3454	0.8073	0.09868	0.9935	0.687	0.4346
ATP13A5	-	-	-	-	-	rs4687402	intronic	0.3747	0.6306	0.4383	0.8639	0.276	0.4989	0.2268	0.916	0.4484
ATP13A5	-	-	-	-	-	rs7648423	intronic	0.4493	0.7896	0.5821	0.3847	0.07953	0.1563	0.8969	0.1835	0.1129
ATP13A5	-	-	-	-	-	rs2367605	intronic	0.554	0.5004	0.5653	0.3952	0.2087	0.2931	0.4893	0.7215	0.9221
ATP13A5	-	-	-	-	-	rs2367478	intronic	0.7671	0.2973	0.0484	0.9932	0.1132	0.2225	0.6143	0.07177	0.2115
ATP13A5	-	-	-	-	-	rs4687401	intronic	0.7979	0.1483	0.857	0.1031	0.1076	0.7491	0.3178	0.9845	0.2726

ATP13A5	-	-	-	-	-	rs3845934	intronic	0.8163	0.3082	0.106	0.8656	0.1835	0.5584	0.2442	0.02604	0.1167
ATP13A5	-	-	-	-	-	rs951016	intronic	0.8179	0.8243	0.0532	0.5675	0.5126	0.4937	0.5248	0.4513	0.5736
PCDHA12	1.19E-04	TRUE	5	140254886	2589	rs269554	intronic	0.172	0.9766	0.5146	0.4041	0.1262	0.6255	0.8182	0.07802	0.8124
PCDHA12	-	-	-	-	-	rs269546	intronic	0.1805	0.9864	0.4715	0.4044	0.09991	0.6143	0.804	0.06408	0.7864
PCDHA12	-	-	-	-	-	rs155806	intronic	0.2168	0.09095	0.7046	0.7259	0.2123	0.7691	0.8478	0.08984	0.9226
PCDHA12	-	-	-	-	-	rs246037	intronic	0.2583	0.5916	0.6191	0.7621	0.501	0.03542	0.3006	0.4306	0.009449
PCDHA12	-	-	-	-	-	rs251373	intronic	0.2703	0.6915	1.37E-04	0.959	0.4968	0.05143	0.3177	2.32E-06	0.005523
PCDHA12	-	-	-	-	-	rs31874	intronic	0.5322	0.11	0.01766	0.5221	0.1389	0.9701	0.8483	9.46E-04	0.2634
PCDHA12	-	-	-	-	-	rs155818	intronic	0.602	0.8108	3.89E-04	0.3021	0.7364	0.116	0.2696	8.10E-04	0.007192
PCDHA12	-	-	-	-	-	rs246001	intronic	0.6057	0.3398	0.6824	0.376	0.2115	0.8984	0.8386	0.1796	0.9351
PCDHA12	-	-	-	-	-	rs56003	intronic	0.6209	0.7788	3.28E-04	0.3059	0.7124	0.1282	0.2885	6.17E-04	0.008587
PCDHA12	-	-	-	-	-	rs3756325	intronic	0.9869	0.7665	1.14E-04	0.3402	0.3546	0.1971	0.1832	5.02E-04	0.05585
PTPRJ	1.19E-04	TRUE	11	48002109	152158	rs10501322	intronic	0.04156	0.8967	0.04147	0.4022	0.9701	0.3717	0.1639	0.03687	0.9259
PTPRJ	-	-	-	-	-	rs12798703	3UTR	0.07671	0.2195	0.5317	0.8505	0.02903	0.05651	0.7881	0.8296	0.8115
PTPRJ	-	-	-	-	-	rs12099166	intronic	0.1116	0.449	0.9027	0.9566	0.05655	0.1921	0.6551	0.6224	0.8349
PTPRJ	-	-	-	-	-	rs11601310	intronic	0.5728	0.335	0.1467	0.1877	0.1378	0.8408	0.527	0.04301	0.1864
PTPRJ	-	-	-	-	-	rs1566729	intronic	0.5883	0.2087	0.02756	0.4156	1.80E-04	0.8809	0.6454	0.02975	0.8311
PTPRJ	-	-	-	-	-	rs10769317	intronic	0.6342	0.2852	0.02352	0.752	0.008523	0.6036	0.4857	0.009306	0.6683
PTPRJ	-	-	-	-	-	rs1875696	intronic	0.6862	0.289	0.0324	0.7004	0.01341	0.5225	0.5254	0.0183	0.8059
PTPRJ	-	-	-	-	-	rs1910364	intronic	0.7033	0.2947	0.2324	0.5092	0.0152	0.2471	0.948	0.1169	0.7689
PTPRJ	-	-	-	-	-	rs6485802	intronic	0.7415	0.364	0.4883	0.291	0.002874	0.3405	0.8034	0.2229	0.6868
PTPRJ	-	-	-	-	-	rs4752897	intronic	0.7504	0.2933	0.2588	0.4624	0.01248	0.2397	0.9623	0.1336	0.7603
PTPRJ	-	-	-	-	-	rs2270994	intronic	0.7849	0.9343	0.1984	0.3406	0.008102	0.7751	0.6791	0.06182	0.7134
PTPRJ	-	-	-	-	-	rs1113480	intronic	0.8941	0.4773	0.4627	0.818	9.83E-04	0.5182	0.9286	0.1389	0.866
PTPRJ	-	-	-	-	-	rs7946766	intronic	0.9263	0.3948	0.7952	0.5433	1.35E-06	0.3734	0.6843	0.3887	0.8044
PTPRJ	-	-	-	-	-	rs1503185	exonic	0.937	0.2914	0.3694	0.06634	0.7666	0.7513	0.7955	0.6177	0.1593
PTPRJ	-	-	-	-	-	rs1566734	exonic	0.9622	0.2686	0.3947	0.06836	0.714	0.7946	0.7678	0.6288	0.1534
PTPRJ	-	-	-	-	-	rs3741410	intronic	0.9668	0.1554	0.1519	0.365	9.17E-05	0.3665	0.7982	0.1049	0.9464
PTPRJ	-	-	-	-	-	rs905476	intronic	0.9691	0.3896	0.3947	0.06224	0.5952	0.7955	0.7852	0.7104	0.1983
PCDHA8	1.21E-04	TRUE	5	140220906	171024	rs269554	intronic	0.172	0.9766	0.5146	0.4041	0.1262	0.6255	0.8182	0.07802	0.8124
PCDHA8	-	-	-	-	-	rs269546	intronic	0.1805	0.9864	0.4715	0.4044	0.09991	0.6143	0.804	0.06408	0.7864
PCDHA8	-	-	-	-	-	rs155806	intronic	0.2168	0.09095	0.7046	0.7259	0.2123	0.7691	0.8478	0.08984	0.9226
PCDHA8	-	-	-	-	-	rs246037	intronic	0.2583	0.5916	0.6191	0.7621	0.501	0.03542	0.3006	0.4306	0.009449

PCDHA8	-	-	-	-	-	rs251370	intronic	0.2676	0.6527	1.46E-04	0.94	0.4745	0.05209	0.345	2.84E-06	0.007411
PCDHA8	-	-	-	-	-	rs251373	intronic	0.2703	0.6915	1.37E-04	0.959	0.4968	0.05143	0.3177	2.32E-06	0.005523
PCDHA8	-	-	-	-	-	rs31874	intronic	0.5322	0.11	0.01766	0.5221	0.1389	0.9701	0.8483	9.46E-04	0.2634
PCDHA8	-	-	-	-	-	rs155818	intronic	0.602	0.8108	3.89E-04	0.3021	0.7364	0.116	0.2696	8.10E-04	0.007192
PCDHA8	-	-	-	-	-	rs246001	intronic	0.6057	0.3398	0.6824	0.376	0.2115	0.8984	0.8386	0.1796	0.9351
PCDHA8	-	-	-	-	-	rs56003	intronic	0.6209	0.7788	3.28E-04	0.3059	0.7124	0.1282	0.2885	6.17E-04	0.008587
PCDHA8	-	-	-	-	-	rs3756325	intronic	0.9869	0.7665	1.14E-04	0.3402	0.3546	0.1971	0.1832	5.02E-04	0.05585
PCDHA9	1.21E-04	TRUE	5	140227356	164574	rs269554	intronic	0.172	0.9766	0.5146	0.4041	0.1262	0.6255	0.8182	0.07802	0.8124
PCDHA9	-	-	-	-	-	rs269546	intronic	0.1805	0.9864	0.4715	0.4044	0.09991	0.6143	0.804	0.06408	0.7864
PCDHA9	-	-	-	-	-	rs155806	intronic	0.2168	0.09095	0.7046	0.7259	0.2123	0.7691	0.8478	0.08984	0.9226
PCDHA9	-	-	-	-	-	rs246037	intronic	0.2583	0.5916	0.6191	0.7621	0.501	0.03542	0.3006	0.4306	0.009449
PCDHA9	-	-	-	-	-	rs251370	intronic	0.2676	0.6527	1.46E-04	0.94	0.4745	0.05209	0.345	2.84E-06	0.007411
PCDHA9	-	-	-	-	-	rs251373	intronic	0.2703	0.6915	1.37E-04	0.959	0.4968	0.05143	0.3177	2.32E-06	0.005523
PCDHA9	-	-	-	-	-	rs31874	intronic	0.5322	0.11	0.01766	0.5221	0.1389	0.9701	0.8483	9.46E-04	0.2634
PCDHA9	-	-	-	-	-	rs155818	intronic	0.602	0.8108	3.89E-04	0.3021	0.7364	0.116	0.2696	8.10E-04	0.007192
PCDHA9	-	-	-	-	-	rs246001	intronic	0.6057	0.3398	0.6824	0.376	0.2115	0.8984	0.8386	0.1796	0.9351
PCDHA9	-	-	-	-	-	rs56003	intronic	0.6209	0.7788	3.28E-04	0.3059	0.7124	0.1282	0.2885	6.17E-04	0.008587
PCDHA9	-	-	-	-	-	rs3756325	intronic	0.9869	0.7665	1.14E-04	0.3402	0.3546	0.1971	0.1832	5.02E-04	0.05585
PCDHA10	1.21E-04	TRUE	5	140235467	156463	rs269554	intronic	0.172	0.9766	0.5146	0.4041	0.1262	0.6255	0.8182	0.07802	0.8124
PCDHA10	-	-	-	-	-	rs269546	intronic	0.1805	0.9864	0.4715	0.4044	0.09991	0.6143	0.804	0.06408	0.7864
PCDHA10	-	-	-	-	-	rs155806	intronic	0.2168	0.09095	0.7046	0.7259	0.2123	0.7691	0.8478	0.08984	0.9226
PCDHA10	-	-	-	-	-	rs246037	intronic	0.2583	0.5916	0.6191	0.7621	0.501	0.03542	0.3006	0.4306	0.009449
PCDHA10	-	-	-	-	-	rs251370	intronic	0.2676	0.6527	1.46E-04	0.94	0.4745	0.05209	0.345	2.84E-06	0.007411
PCDHA10	-	-	-	-	-	rs251373	intronic	0.2703	0.6915	1.37E-04	0.959	0.4968	0.05143	0.3177	2.32E-06	0.005523
PCDHA10	-	-	-	-	-	rs31874	intronic	0.5322	0.11	0.01766	0.5221	0.1389	0.9701	0.8483	9.46E-04	0.2634
PCDHA10	-	-	-	-	-	rs155818	intronic	0.602	0.8108	3.89E-04	0.3021	0.7364	0.116	0.2696	8.10E-04	0.007192
PCDHA10	-	-	-	-	-	rs246001	intronic	0.6057	0.3398	0.6824	0.376	0.2115	0.8984	0.8386	0.1796	0.9351
PCDHA10	-	-	-	-	-	rs56003	intronic	0.6209	0.7788	3.28E-04	0.3059	0.7124	0.1282	0.2885	6.17E-04	0.008587
PCDHA10	-	-	-	-	-	rs3756325	intronic	0.9869	0.7665	1.14E-04	0.3402	0.3546	0.1971	0.1832	5.02E-04	0.05585
PCDHA11	1.21E-04	TRUE	5	140247097	4089	rs269554	intronic	0.172	0.9766	0.5146	0.4041	0.1262	0.6255	0.8182	0.07802	0.8124
PCDHA11	-	-	-	-	-	rs269546	intronic	0.1805	0.9864	0.4715	0.4044	0.09991	0.6143	0.804	0.06408	0.7864
PCDHA11	-	-	-	-	-	rs155806	intronic	0.2168	0.09095	0.7046	0.7259	0.2123	0.7691	0.8478	0.08984	0.9226
PCDHA11	-	-	-	-	-	rs246037	intronic	0.2583	0.5916	0.6191	0.7621	0.501	0.03542	0.3006	0.4306	0.009449

PCDHA11	-	-	-	-	-	rs251370	intronic	0.2676	0.6527	1.46E-04	0.94	0.4745	0.05209	0.345	2.84E-06	0.007411
PCDHA11	-	-	-	-	-	rs251373	intronic	0.2703	0.6915	1.37E-04	0.959	0.4968	0.05143	0.3177	2.32E-06	0.005523
PCDHA11	-	-	-	-	-	rs31874	intronic	0.5322	0.11	0.01766	0.5221	0.1389	0.9701	0.8483	9.46E-04	0.2634
PCDHA11	-	-	-	-	-	rs155818	intronic	0.602	0.8108	3.89E-04	0.3021	0.7364	0.116	0.2696	8.10E-04	0.007192
PCDHA11	-	-	-	-	-	rs246001	intronic	0.6057	0.3398	0.6824	0.376	0.2115	0.8984	0.8386	0.1796	0.9351
PCDHA11	-	-	-	-	-	rs56003	intronic	0.6209	0.7788	3.28E-04	0.3059	0.7124	0.1282	0.2885	6.17E-04	0.008587
PCDHA11	-	-	-	-	-	rs3756325	intronic	0.9869	0.7665	1.14E-04	0.3402	0.3546	0.1971	0.1832	5.02E-04	0.05585
LDLR	1.22E-04	TRUE	19	11200037	44469	rs2228671	exonic	0.01898	0.6	0.1552	0.02482	0.9062	0.2885	2.34E-06	0.7329	0.9259
LDLR	-	-	-	-	-	rs688	exonic	0.2571	0.2648	0.7494	0.7488	0.3062	0.2784	0.4781	0.7456	0.7798
LDLR	-	-	-	-	-	rs1433099	3UTR	0.2671	0.1432	0.7847	0.7041	0.08323	0.3458	0.1243	0.3984	0.1992
LDLR	-	-	-	-	-	rs1799898	exonic	0.2866	0.01184	0.2402	0.07395	0.544	0.3625	0.5266	0.5658	0.937
LDLR	-	-	-	-	-	rs2569538	intronic	0.4305	0.4183	0.5935	0.09449	0.3066	0.5847	0.05369	0.3916	0.3261
LDLR	-	-	-	-	-	rs5930	exonic	0.7831	0.6627	0.3461	0.7082	0.5653	0.8159	0.2087	0.9924	0.631
LDLR	-	-	-	-	-	rs2569537	intronic	0.8101	0.9703	0.5642	0.8918	0.2059	0.9675	0.5865	0.15	0.7931
PCDHA6	1.35E-04	TRUE	5	140207506	2583	rs269554	intronic	0.172	0.9766	0.5146	0.4041	0.1262	0.6255	0.8182	0.07802	0.8124
PCDHA6	-	-	-	-	-	rs269546	intronic	0.1805	0.9864	0.4715	0.4044	0.09991	0.6143	0.804	0.06408	0.7864
PCDHA6	-	-	-	-	-	rs155806	intronic	0.2168	0.09095	0.7046	0.7259	0.2123	0.7691	0.8478	0.08984	0.9226
PCDHA6	-	-	-	-	-	rs246037	intronic	0.2583	0.5916	0.6191	0.7621	0.501	0.03542	0.3006	0.4306	0.009449
PCDHA6	-	-	-	-	-	rs251370	intronic	0.2676	0.6527	1.46E-04	0.94	0.4745	0.05209	0.345	2.84E-06	0.007411
PCDHA6	-	-	-	-	-	rs251373	intronic	0.2703	0.6915	1.37E-04	0.959	0.4968	0.05143	0.3177	2.32E-06	0.005523
PCDHA6	-	-	-	-	-	rs31874	intronic	0.5322	0.11	0.01766	0.5221	0.1389	0.9701	0.8483	9.46E-04	0.2634
PCDHA6	-	-	-	-	-	rs10038174	intronic	0.5817	0.652	8.43E-04	0.9749	0.9338	0.4384	0.2682	2.64E-04	0.03247
PCDHA6	-	-	-	-	-	rs155818	intronic	0.602	0.8108	3.89E-04	0.3021	0.7364	0.116	0.2696	8.10E-04	0.007192
PCDHA6	-	-	-	-	-	rs246001	intronic	0.6057	0.3398	0.6824	0.376	0.2115	0.8984	0.8386	0.1796	0.9351
PCDHA6	-	-	-	-	-	rs56003	intronic	0.6209	0.7788	3.28E-04	0.3059	0.7124	0.1282	0.2885	6.17E-04	0.008587
PCDHA6	-	-	-	-	-	rs3756325	intronic	0.9869	0.7665	1.14E-04	0.3402	0.3546	0.1971	0.1832	5.02E-04	0.05585
PCDHA7	1.35E-04	TRUE	5	140213968	2371	rs269554	intronic	0.172	0.9766	0.5146	0.4041	0.1262	0.6255	0.8182	0.07802	0.8124
PCDHA7	-	-	-	-	-	rs269546	intronic	0.1805	0.9864	0.4715	0.4044	0.09991	0.6143	0.804	0.06408	0.7864
PCDHA7	-	-	-	-	-	rs155806	intronic	0.2168	0.09095	0.7046	0.7259	0.2123	0.7691	0.8478	0.08984	0.9226
PCDHA7	-	-	-	-	-	rs246037	intronic	0.2583	0.5916	0.6191	0.7621	0.501	0.03542	0.3006	0.4306	0.009449
PCDHA7	-	-	-	-	-	rs251370	intronic	0.2676	0.6527	1.46E-04	0.94	0.4745	0.05209	0.345	2.84E-06	0.007411
PCDHA7	-	-	-	-	-	rs251373	intronic	0.2703	0.6915	1.37E-04	0.959	0.4968	0.05143	0.3177	2.32E-06	0.005523
PCDHA7	-	-	-	-	-	rs31874	intronic	0.5322	0.11	0.01766	0.5221	0.1389	0.9701	0.8483	9.46E-04	0.2634

PCDHA7	-	-	-	-	-	rs10038174	intronic	0.5817	0.652	8.43E-04	0.9749	0.9338	0.4384	0.2682	2.64E-04	0.03247
PCDHA7	-	-	-	-	-	rs155818	intronic	0.602	0.8108	3.89E-04	0.3021	0.7364	0.116	0.2696	8.10E-04	0.007192
PCDHA7	-	-	-	-	-	rs246001	intronic	0.6057	0.3398	0.6824	0.376	0.2115	0.8984	0.8386	0.1796	0.9351
PCDHA7	-	-	-	-	-	rs56003	intronic	0.6209	0.7788	3.28E-04	0.3059	0.7124	0.1282	0.2885	6.17E-04	0.008587
PCDHA7	-	-	-	-	-	rs3756325	intronic	0.9869	0.7665	1.14E-04	0.3402	0.3546	0.1971	0.1832	5.02E-04	0.05585
PCDHA5	1.42E-04	TRUE	5	140201360	190570	rs269554	intronic	0.172	0.9766	0.5146	0.4041	0.1262	0.6255	0.8182	0.07802	0.8124
PCDHA5	-	-	-	-	-	rs269546	intronic	0.1805	0.9864	0.4715	0.4044	0.09991	0.6143	0.804	0.06408	0.7864
PCDHA5	-	-	-	-	-	rs155806	intronic	0.2168	0.09095	0.7046	0.7259	0.2123	0.7691	0.8478	0.08984	0.9226
PCDHA5	-	-	-	-	-	rs246037	intronic	0.2583	0.5916	0.6191	0.7621	0.501	0.03542	0.3006	0.4306	0.009449
PCDHA5	-	-	-	-	-	rs251370	intronic	0.2676	0.6527	1.46E-04	0.94	0.4745	0.05209	0.345	2.84E-06	0.007411
PCDHA5	-	-	-	-	-	rs251373	intronic	0.2703	0.6915	1.37E-04	0.959	0.4968	0.05143	0.3177	2.32E-06	0.005523
PCDHA5	-	-	-	-	-	rs7732179	intronic	0.4129	0.8263	0.001359	0.9532	0.9488	0.2113	0.2028	4.25E-04	0.02154
PCDHA5	-	-	-	-	-	rs31874	intronic	0.5322	0.11	0.01766	0.5221	0.1389	0.9701	0.8483	9.46E-04	0.2634
PCDHA5	-	-	-	-	-	rs10038174	intronic	0.5817	0.652	8.43E-04	0.9749	0.9338	0.4384	0.2682	2.64E-04	0.03247
PCDHA5	-	-	-	-	-	rs155818	intronic	0.602	0.8108	3.89E-04	0.3021	0.7364	0.116	0.2696	8.10E-04	0.007192
PCDHA5	-	-	-	-	-	rs246001	intronic	0.6057	0.3398	0.6824	0.376	0.2115	0.8984	0.8386	0.1796	0.9351
PCDHA5	-	-	-	-	-	rs56003	intronic	0.6209	0.7788	3.28E-04	0.3059	0.7124	0.1282	0.2885	6.17E-04	0.008587
PCDHA5	-	-	-	-	-	rs3756325	intronic	0.9869	0.7665	1.14E-04	0.3402	0.3546	0.1971	0.1832	5.02E-04	0.05585
PCDHA4	1.43E-04	TRUE	5	140186658	205272	rs269554	intronic	0.172	0.9766	0.5146	0.4041	0.1262	0.6255	0.8182	0.07802	0.8124
PCDHA4	-	-	-	-	-	rs269546	intronic	0.1805	0.9864	0.4715	0.4044	0.09991	0.6143	0.804	0.06408	0.7864
PCDHA4	-	-	-	-	-	rs155806	intronic	0.2168	0.09095	0.7046	0.7259	0.2123	0.7691	0.8478	0.08984	0.9226
PCDHA4	-	-	-	-	-	rs246037	intronic	0.2583	0.5916	0.6191	0.7621	0.501	0.03542	0.3006	0.4306	0.009449
PCDHA4	-	-	-	-	-	rs251370	intronic	0.2676	0.6527	1.46E-04	0.94	0.4745	0.05209	0.345	2.84E-06	0.007411
PCDHA4	-	-	-	-	-	rs251373	intronic	0.2703	0.6915	1.37E-04	0.959	0.4968	0.05143	0.3177	2.32E-06	0.005523
PCDHA4	-	-	-	-	-	rs7732179	intronic	0.4129	0.8263	0.001359	0.9532	0.9488	0.2113	0.2028	4.25E-04	0.02154
PCDHA4	-	-	-	-	-	rs31874	intronic	0.5322	0.11	0.01766	0.5221	0.1389	0.9701	0.8483	9.46E-04	0.2634
PCDHA4	-	-	-	-	-	rs3822346	exonic	0.5805	0.7103	6.72E-04	0.9879	0.851	0.3846	0.2975	3.23E-04	0.03016
PCDHA4	-	-	-	-	-	rs10038174	intronic	0.5817	0.652	8.43E-04	0.9749	0.9338	0.4384	0.2682	2.64E-04	0.03247
PCDHA4	-	-	-	-	-	rs1030166	intronic	0.5961	0.7974	5.70E-04	0.9921	0.8504	0.3942	0.345	2.69E-04	0.0367
PCDHA4	-	-	-	-	-	rs155818	intronic	0.602	0.8108	3.89E-04	0.3021	0.7364	0.116	0.2696	8.10E-04	0.007192
PCDHA4	-	-	-	-	-	rs246001	intronic	0.6057	0.3398	0.6824	0.376	0.2115	0.8984	0.8386	0.1796	0.9351
PCDHA4	-	-	-	-	-	rs56003	intronic	0.6209	0.7788	3.28E-04	0.3059	0.7124	0.1282	0.2885	6.17E-04	0.008587
PCDHA4	-	-	-	-	-	rs3756325	intronic	0.9869	0.7665	1.14E-04	0.3402	0.3546	0.1971	0.1832	5.02E-04	0.05585

PCDHA1	1.43E-04	TRUE	5	140165720	226210	rs269554	intronic	0.172	0.9766	0.5146	0.4041	0.1262	0.6255	0.8182	0.07802	0.8124
PCDHA1	-	-	-	-	-	rs269546	intronic	0.1805	0.9864	0.4715	0.4044	0.09991	0.6143	0.804	0.06408	0.7864
PCDHA1	-	-	-	-	-	rs155806	intronic	0.2168	0.09095	0.7046	0.7259	0.2123	0.7691	0.8478	0.08984	0.9226
PCDHA1	-	-	-	-	-	rs246037	intronic	0.2583	0.5916	0.6191	0.7621	0.501	0.03542	0.3006	0.4306	0.009449
PCDHA1	-	-	-	-	-	rs251370	intronic	0.2676	0.6527	1.46E-04	0.94	0.4745	0.05209	0.345	2.84E-06	0.007411
PCDHA1	-	-	-	-	-	rs251373	intronic	0.2703	0.6915	1.37E-04	0.959	0.4968	0.05143	0.3177	2.32E-06	0.005523
PCDHA1	-	-	-	-	-	rs7732179	intronic	0.4129	0.8263	0.001359	0.9532	0.9488	0.2113	0.2028	4.25E-04	0.02154
PCDHA1	-	-	-	-	-	rs31874	intronic	0.5322	0.11	0.01766	0.5221	0.1389	0.9701	0.8483	9.46E-04	0.2634
PCDHA1	-	-	-	-	-	rs3822346	exonic	0.5805	0.7103	6.72E-04	0.9879	0.851	0.3846	0.2975	3.23E-04	0.03016
PCDHA1	-	-	-	-	-	rs10038174	intronic	0.5817	0.652	8.43E-04	0.9749	0.9338	0.4384	0.2682	2.64E-04	0.03247
PCDHA1	-	-	-	-	-	rs3756338	intronic	0.5832	0.6992	6.68E-04	0.9808	0.8712	0.3875	0.3033	3.52E-04	0.03019
PCDHA1	-	-	-	-	-	rs1030166	intronic	0.5961	0.7974	5.70E-04	0.9921	0.8504	0.3942	0.345	2.69E-04	0.0367
PCDHA1	-	-	-	-	-	rs155818	intronic	0.602	0.8108	3.89E-04	0.3021	0.7364	0.116	0.2696	8.10E-04	0.007192
PCDHA1	-	-	-	-	-	rs246001	intronic	0.6057	0.3398	0.6824	0.376	0.2115	0.8984	0.8386	0.1796	0.9351
PCDHA1	-	-	-	-	-	rs56003	intronic	0.6209	0.7788	3.28E-04	0.3059	0.7124	0.1282	0.2885	6.17E-04	0.008587
PCDHA1	-	-	-	-	-	rs3756325	intronic	0.9869	0.7665	1.14E-04	0.3402	0.3546	0.1971	0.1832	5.02E-04	0.05585
PCDHA3	1.43E-04	TRUE	5	140180782	2476	rs269554	intronic	0.172	0.9766	0.5146	0.4041	0.1262	0.6255	0.8182	0.07802	0.8124
PCDHA3	-	-	-	-	-	rs269546	intronic	0.1805	0.9864	0.4715	0.4044	0.09991	0.6143	0.804	0.06408	0.7864
PCDHA3	-	-	-	-	-	rs155806	intronic	0.2168	0.09095	0.7046	0.7259	0.2123	0.7691	0.8478	0.08984	0.9226
PCDHA3	-	-	-	-	-	rs246037	intronic	0.2583	0.5916	0.6191	0.7621	0.501	0.03542	0.3006	0.4306	0.009449
PCDHA3	-	-	-	-	-	rs251370	intronic	0.2676	0.6527	1.46E-04	0.94	0.4745	0.05209	0.345	2.84E-06	0.007411
PCDHA3	-	-	-	-	-	rs251373	intronic	0.2703	0.6915	1.37E-04	0.959	0.4968	0.05143	0.3177	2.32E-06	0.005523
PCDHA3	-	-	-	-	-	rs7732179	intronic	0.4129	0.8263	0.001359	0.9532	0.9488	0.2113	0.2028	4.25E-04	0.02154
PCDHA3	-	-	-	-	-	rs31874	intronic	0.5322	0.11	0.01766	0.5221	0.1389	0.9701	0.8483	9.46E-04	0.2634
PCDHA3	-	-	-	-	-	rs3822346	exonic	0.5805	0.7103	6.72E-04	0.9879	0.851	0.3846	0.2975	3.23E-04	0.03016
PCDHA3	-	-	-	-	-	rs10038174	intronic	0.5817	0.652	8.43E-04	0.9749	0.9338	0.4384	0.2682	2.64E-04	0.03247
PCDHA3	-	-	-	-	-	rs3756338	intronic	0.5832	0.6992	6.68E-04	0.9808	0.8712	0.3875	0.3033	3.52E-04	0.03019
PCDHA3	-	-	-	-	-	rs1030166	intronic	0.5961	0.7974	5.70E-04	0.9921	0.8504	0.3942	0.345	2.69E-04	0.0367
PCDHA3	-	-	-	-	-	rs155818	intronic	0.602	0.8108	3.89E-04	0.3021	0.7364	0.116	0.2696	8.10E-04	0.007192
PCDHA3	-	-	-	-	-	rs246001	intronic	0.6057	0.3398	0.6824	0.376	0.2115	0.8984	0.8386	0.1796	0.9351
PCDHA3	-	-	-	-	-	rs56003	intronic	0.6209	0.7788	3.28E-04	0.3059	0.7124	0.1282	0.2885	6.17E-04	0.008587
PCDHA3	-	-	-	-	-	rs3756325	intronic	0.9869	0.7665	1.14E-04	0.3402	0.3546	0.1971	0.1832	5.02E-04	0.05585
PCDHA2	1.43E-04	TRUE	5	140174443	2620	rs269554	intronic	0.172	0.9766	0.5146	0.4041	0.1262	0.6255	0.8182	0.07802	0.8124

PCDHA2	-	-	-	-	-	rs269546	intronic	0.1805	0.9864	0.4715	0.4044	0.09991	0.6143	0.804	0.06408	0.7864
PCDHA2	-	-	-	-	-	rs155806	intronic	0.2168	0.09095	0.7046	0.7259	0.2123	0.7691	0.8478	0.08984	0.9226
PCDHA2	-	-	-	-	-	rs246037	intronic	0.2583	0.5916	0.6191	0.7621	0.501	0.03542	0.3006	0.4306	0.009449
PCDHA2	-	-	-	-	-	rs251370	intronic	0.2676	0.6527	1.46E-04	0.94	0.4745	0.05209	0.345	2.84E-06	0.007411
PCDHA2	-	-	-	-	-	rs251373	intronic	0.2703	0.6915	1.37E-04	0.959	0.4968	0.05143	0.3177	2.32E-06	0.005523
PCDHA2	-	-	-	-	-	rs7732179	intronic	0.4129	0.8263	0.001359	0.9532	0.9488	0.2113	0.2028	4.25E-04	0.02154
PCDHA2	-	-	-	-	-	rs31874	intronic	0.5322	0.11	0.01766	0.5221	0.1389	0.9701	0.8483	9.46E-04	0.2634
PCDHA2	-	-	-	-	-	rs3822346	exonic	0.5805	0.7103	6.72E-04	0.9879	0.851	0.3846	0.2975	3.23E-04	0.03016
PCDHA2	-	-	-	-	-	rs10038174	intronic	0.5817	0.652	8.43E-04	0.9749	0.9338	0.4384	0.2682	2.64E-04	0.03247
PCDHA2	-	-	-	-	-	rs3756338	intronic	0.5832	0.6992	6.68E-04	0.9808	0.8712	0.3875	0.3033	3.52E-04	0.03019
PCDHA2	-	-	-	-	-	rs1030166	intronic	0.5961	0.7974	5.70E-04	0.9921	0.8504	0.3942	0.345	2.69E-04	0.0367
PCDHA2	-	-	-	-	-	rs155818	intronic	0.602	0.8108	3.89E-04	0.3021	0.7364	0.116	0.2696	8.10E-04	0.007192
PCDHA2	-	-	-	-	-	rs246001	intronic	0.6057	0.3398	0.6824	0.376	0.2115	0.8984	0.8386	0.1796	0.9351
PCDHA2	-	-	-	-	-	rs56003	intronic	0.6209	0.7788	3.28E-04	0.3059	0.7124	0.1282	0.2885	6.17E-04	0.008587
PCDHA2	-	-	-	-	-	rs3756325	intronic	0.9869	0.7665	1.14E-04	0.3402	0.3546	0.1971	0.1832	5.02E-04	0.05585
ASB18	1.47E-04	TRUE	2	237103514	69475	rs13386897	downstream	2.47E-06	0.8287	0.1551	0.3079	0.005644	0.0215	0.473	0.0262	0.9886
ASB18	-	-	-	-	-	rs10177957	exonic	5.23E-06	0.9639	0.173	0.3605	0.006755	0.03403	0.6251	0.03719	0.9019
ASB18	-	-	-	-	-	rs1530952	intronic	2.57E-05	0.2764	0.5421	0.4565	0.009487	0.08064	0.3651	0.1312	0.6673
ASB18	-	-	-	-	-	rs6757571	intronic	1.73E-04	0.1497	0.7752	0.3153	0.004396	0.07963	0.6301	0.3757	0.7732
ASB18	-	-	-	-	-	rs6431443	intronic	3.47E-04	0.8836	0.5678	0.3374	0.04232	0.2846	0.3886	0.2773	0.4349
ASB18	-	-	-	-	-	rs6756597	exonic	0.004905	0.1725	0.796	0.7264	0.05103	0.6014	0.2579	0.04511	0.8992
ASB18	-	-	-	-	-	rs2015983	intronic	0.0456	0.3355	0.8039	0.214	0.4633	0.2372	0.523	0.9838	0.502
ASB18	-	-	-	-	-	rs1377446	intronic	0.2548	0.8725	0.09521	0.952	0.5182	0.5436	0.6881	0.7001	0.9632
ASB18	-	-	-	-	-	rs13390159	intronic	0.7346	0.3824	0.5828	0.8681	0.1266	0.2073	0.2332	0.8883	0.9934
HAS3	1.54E-04	TRUE	16	69140128	11443	rs3785079	intronic	0.02107	0.2826	0.6739	0.5005	1.98E-04	0.734	0.6707	0.9628	0.4028
HAS3	-	-	-	-	-	rs2232228	exonic	0.04286	0.007179	0.7189	0.5688	7.15E-06	0.3812	0.02884	0.4314	0.008064
HAS3	-	-	-	-	-	rs8047014	upstream	0.07218	0.03269	0.8194	0.226	1.32E-04	0.9262	0.9125	0.5256	0.05329
ZNF101	1.59E-04	TRUE	19	19779662	11477	rs7247433	downstream	0.6669	0.7983	0.3423	0.45	0.07522	0.4211	0.04665	0.6371	0.04472
ZNF101	-	-	-	-	-	rs2304130	intronic	0.7992	0.3232	0.669	0.3771	0.672	0.8025	0.003638	0.3919	9.71E-06
PPP1R2P3	1.61E-04	TRUE	5	156277548	1992	rs4704810	upstream	0.02801	0.193	0.7231	0.5071	0.8873	0.5869	0.4176	0.1802	0.07457
PPP1R2P3	-	-	-	-	-	rs4476703	ncRNA	0.2789	0.6061	0.5614	0.0284	0.3274	5.59E-04	0.8088	0.5761	9.42E-06
PPP1R2P3	-	-	-	-	-	rs10060710	downstream	0.5482	0.7822	0.4348	0.05966	0.4248	0.001767	0.6839	0.5201	1.87E-05
FEN1	1.65E-04	TRUE	11	61560108	4607	rs695867	intronic	0.1076	0.4374	0.7756	0.3997	0.1468	0.09912	0.4622	0.4798	0.0539

FEN1	-	-	-	-	-	rs102275	intronic	0.4241	0.6986	0.9772	0.2076	0.04303	0.4165	6.51E-06	0.3705	0.01715
FEN1	-	-	-	-	-	rs412334	5UTR	0.5315	0.04424	0.442	0.9345	0.5911	0.3789	0.06239	0.301	0.7839
MIR611	1.65E-04	TRUE	11	61559966	68	rs695867	intronic	0.1076	0.4374	0.7756	0.3997	0.1468	0.09912	0.4622	0.4798	0.0539
MIR611	-	-	-	-	-	rs102275	intronic	0.4241	0.6986	0.9772	0.2076	0.04303	0.4165	6.51E-06	0.3705	0.01715
MIR611	-	-	-	-	-	rs412334	5UTR	0.5315	0.04424	0.442	0.9345	0.5911	0.3789	0.06239	0.301	0.7839
DNM2	1.67E-04	TRUE	19	10828728	113859	rs4804528	intronic	0.03157	0.7681	0.1921	0.1958	0.1698	0.7902	0.1504	0.05531	0.5621
DNM2	-	-	-	-	-	rs4804523	intronic	0.04364	0.7893	0.367	0.1006	0.007919	0.9186	0.6791	0.7825	0.04678
DNM2	-	-	-	-	-	rs12974306	intronic	0.04808	0.8278	0.6042	0.3159	0.09428	0.8086	0.2042	0.8379	0.2581
DNM2	-	-	-	-	-	rs7247567	intronic	0.0604	0.8542	0.4653	0.04002	0.002847	0.8967	0.8851	0.9333	0.03646
DNM2	-	-	-	-	-	rs10404348	intronic	0.06073	0.9344	0.3047	0.1589	0.01078	0.9715	0.8548	0.8939	0.08716
DNM2	-	-	-	-	-	rs2043332	intronic	0.0871	0.536	0.2756	0.1703	0.1096	0.7182	0.1121	0.03938	0.304
DNM2	-	-	-	-	-	rs11881315	intronic	0.221	0.3192	0.3849	0.7057	0.1112	0.7115	3.05E-06	0.03572	0.67
DNM2	-	-	-	-	-	rs3760781	upstream	0.2492	0.1677	0.9382	0.5944	0.3132	0.3131	2.90E-04	0.3736	0.4357
DNM2	-	-	-	-	-	rs4334414	intronic	0.8992	0.9775	0.7445	0.08525	0.6641	0.4224	0.3655	0.6707	0.9155
DNM2	-	-	-	-	-	rs11672431	exonic	0.9145	0.3328	0.6379	0.03874	0.1204	0.3157	0.4984	0.5622	0.2097
TMEM258	1.69E-04	TRUE	11	61556601	3485	rs695867	intronic	0.1076	0.4374	0.7756	0.3997	0.1468	0.09912	0.4622	0.4798	0.0539
TMEM258	-	-	-	-	-	rs102275	intronic	0.4241	0.6986	0.9772	0.2076	0.04303	0.4165	6.51E-06	0.3705	0.01715
TMEM258	-	-	-	-	-	rs174537	intronic	0.5105	0.8084	0.929	0.157	0.05822	0.4036	0.4902	0.4465	0.01814
TMEM258	-	-	-	-	-	rs412334	5UTR	0.5315	0.04424	0.442	0.9345	0.5911	0.3789	0.06239	0.301	0.7839
VPS4A	1.74E-04	TRUE	16	69345286	13661	rs246129	3UTR	0.04314	0.01158	0.7735	0.714	4.06E-05	0.4269	0.02144	0.966	0.00443
VPS4A	-	-	-	-	-	rs153050	intronic	0.06378	0.8522	0.9179	0.497	6.09E-06	0.2841	0.01775	0.6722	0.3665
VPS4A	-	-	-	-	-	rs12258	3UTR	0.3105	0.1679	0.3671	0.8741	0.1439	0.7094	0.5366	0.2689	0.4724
VPS4A	-	-	-	-	-	rs1127231	exonic	0.8817	0.03402	0.9768	0.938	0.1605	0.3116	0.2159	0.3674	0.3851

TABLE S10: Annotation of the genes/genetic regions identified by MGAS by reference to the Catalog of Published Genome-wide Association Studies (http://www.genome.gov/gwastudies/)

gene name	Gene name
unique (y/n)	Does the gene overlap with other genes identified in the MGAS analysis?
	That is: do some of the SNPs that represent this gene also represent other genes that were significant in this MGAS analysis?
gene cluster nr	Overlapping genes are assigned to a cluster; these clusters are given an arbitrary number

gene name	unique (y/n)	gene-cluster nr	previously related to metabolic (related) traits (Y/N)?	Reported in orginal papar
PCDHA1	N	1	Y: Visceral adipose tissue	N
PCDHA10	N	1		
PCDHA11	N	1		
PCDHA12	N	1		
PCDHA2	N	1		
PCDHA3	N	1		
PCDHA4	N	1		
PCDHA5	N	1		
PCDHA6	N	1		
PCDHA7	N	1		
PCDHA8	N	1		
PCDHA9	N	1		
FEN1	N	2	Y: Oleic Palmitoleic and Stearic acid plasma levels	N
MIR611	N	2		
TMEM258	N	2		
EDC4	N	3	Y: metabolic syndrome, coronary heart disease	N
NRN1L	N	3		
NUTF2	N	3		
PSKH1	N	3		
GLT8D1	N	4	N: osteoarthritis, bipolar, combination of: autism, bipolar, ADHD, major depression, schizophrenia	
GNL3	N	4		

SNORD19B	N	4		
SNORD69	N	4		
C12orf43	N	5	Y: e.g. metabolic traits, LDL, C-reactive protein	N
HNF1A	N	5		
OASL	N	5		
CELSR2	N	6	Y: e.g., LCL, total cholesterol, lipid metabolism phenotypes	Y
PSRC1	N	6		
PVRL2	N	7	Y: e.g., HDL, waist-to-hip ratio	Y
TOMM40	N	7		
FADS1	N	8	Y: Oleic Palmitoleic and Stearic acid plasma levels	Y
MIR1908	N	8		
EXOC3L1	N	9	Y: coronary heart disease	N
HSF4	N	9		
KIAA0895L	N	9		
NOL3	N	9		
MADD	N	10	Y: e.g. proinsulin, fasting glucose	Y
NR1H3	N	10		
C16orf86	N	11	Y: obesity related traits	N
ENKD1	N	11		
PARD6A	N	11		
FHOD1	N	12	Y: lipoproteins, HDL	N
LRRC29	N	12		
TMEM208	N	12		
APOB	Y	13	Y: LDL	Y
AR	Y	14	Y: LDL	Y
ASB18	Y	15	Y: response to statin therapy	N
ATP13A5	Y	16	Y: triglicerides	N
CETP	Y	17	Y: e.g., HDL, metabolites, metabolic syndrome	Y
CR1L	Y	18	Y: LDL, Erythrocyte sedimentation rate	Y
CRP	Y	19	Y: C-reactive protein, metabolic traits	Y
CTCF	Y	20	Y: HDL	N
CTCFL	Y	21	Y: Type 2 diabetes, response to statine therapy	N
DNM2	Y	22	Y: coronary heart disease	N

DPEP2	Y	23	Y: HDL	N
DPEP3	Y	24	Y: HDL	Y
FAM222B	Y	25	No phenotypes yet reported to our knowledge	N
G6PC2	Y	26	Y: e.g., fasting glucose, metabolite levels, metabolic syndrome	Y
GCKR	Y	27	Y: e.g., glycemic traits, fasting glucose, metabolic syndrome	Y
HAS3	Y	28	N: ADHD	N
HNF1A-AS1	Y	29	Y: LDL, C-reactive protein, Type 2 diabetes	N
HNF4A	Y	30	Y: HDL, C-reactive protein, Type 2 diabetes	N
IGSF5	Y	31	Y: coronary heart disease	N
LDLR	Y	32	Y: LDL, metabolite levels, metabolic syndrome	Y
LEPR	Y	33	Y: obesity, c-reactive proteins, metabolic traits	Y
NFATC3	Y	34	Y: HDL	N
PLA2G15	Y	35	Y: HDL, LDL	N
PPP1R2P3	Y	36	Y: triglicerides	N
PRMT7	Y	37	Y: HDL, lipoproteins, schizophrenia	N
PTPRJ	Y	38	N: D-dimer levels, Acute lymphoblastic leukemia*	N
S1PR2	Y	39	N: thiazide-induced adverse metabolic effects in hypertensive patients	N
SMEK2	Y	40	Y: metabolic syndrome	N
TBL2	Y	41	Y: triglicerides	N
TFAP2B	Y	42	Y: obesity, metabolic syndrome, BMI	N
VPS4A	Y	43	Y: HDL, lipoproteins	N
ZNF101	Y	44	Y: LDL, triglycerides	N

* Note: Glucose metabolism side effects of acute lymphoblastic leukemia have been reported

Table S11: Genome-wide significant SNPs identified by TATES using the 9 continuous metabolic phenotypes

Color coding p-values:		
	red	<.000001
	yellow	.000001-.00001
	green	.00001-.0001
	blue	.0001-.001
	black font	.001-.05
	grey font	>.05

CHRnr	location	rsnr	gene_feature	closest gene*	BMI	CRP	DBP	GLU	HDL	INS	LDL	SBP	TG	TATES p-value
1	109818530	rs646776	downstream	CELSR2, PSRC1, SORT1	0.1401	2.21E-01	0.6273	8.05E-01	1.42E-01	0.07783	2.19E-12	0.5509	5.50E-01	1.84E-11
1	159642349	rs1811472	intergenic	OR10J6P,CRPP1	0.0402	2.27E-15	0.4431	3.39E-01	2.34E-02	0.3052	7.18E-01	0.1728	7.71E-01	1.91E-14
1	159647988	rs12093699	intergenic	OR10J6P,CRPP1, CRP	0.1673	1.62E-13	0.6687	7.37E-01	9.89E-01	0.5228	6.10E-01	0.1764	7.33E-01	1.36E-12
1	159652939	rs2592887	intergenic	OR10J6P,CRPP1	0.06223	1.51E-16	0.3529	1.90E-01	3.41E-02	0.09759	8.22E-01	0.1163	5.97E-01	1.27E-15
1	159678816	rs2794520	downstream	CRPP1, CRP	0.5436	2.92E-22	0.5156	5.87E-01	2.57E-01	0.3577	5.44E-01	0.2896	6.74E-01	2.46E-21
1	159700039	rs11265260	intergenic	CRP, RPL27P2	0.3323	4.45E-09	0.1153	8.43E-01	2.75E-02	0.6661	7.82E-02	0.566	5.81E-01	3.74E-08
2	21206183	rs6754295	intergenic	APOB, C2orf43	0.3326	5.01E-02	0.9046	1.00E-01	3.78E-06	0.8925	1.40E-08	0.5249	9.47E-08	1.18E-07
2	27730940	rs1260326	exonic	GCKR, IFT172, FNDC4, C2orf16,	0.492	7.31E-02	0.5136	3.90E-01	1.86E-01	0.4225	3.07E-01	0.3802	3.56E-10	3.00E-09
				ZNF512, PPM1G, NRBP1, KRTCAP3										
2	169763148	rs560887	intonic	SPC25, FGQTL1, G6PC2, ABCB11	0.6882	4.08E-01	0.9984	5.69E-10	7.24E-01	0.9469	5.26E-01	0.9864	5.29E-01	4.79E-09
6	32765671	rs6932969	intergenic	HLA-DRB,HLA-DQB1	0.3091	1.57E-01	0.5939	5.30E-01	3.91E-03	0.5943	2.99E-11	0.705	7.34E-01	2.51E-10
7	114940068	rs7800941	regulatory reg	MDFIC	0.03904	7.92E-01	0.7859	4.49E-01	4.99E-02	0.4561	8.98E-01	0.4445	5.03E-09	4.24E-08
12	121388962	rs2650000	intergenic	HNF1A, LEF1, RPL12P33,NCRNA00262	0.9205	2.71E-11	0.2698	8.53E-01	6.27E-01	0.6767	3.70E-01	0.5234	7.09E-01	2.28E-10
12	121403724	rs7953249	downstream	HNF1A, C12orf43, OASL, HNF1A-AS1,	0.5548	1.44E-09	0.4425	9.05E-01	4.76E-01	0.5462	2.96E-01	0.6843	9.39E-01	1.21E-08
				AC079602										
12	121431225	rs1169300	intronic	HNF1A	0.6507	2.01E-09	0.6845	8.95E-01	8.55E-01	0.3737	1.91E-01	0.7302	6.95E-01	1.69E-08
12	121435427	rs2464196	exonic	HNF1A, C12orf43, RP11-216P16	0.5168	4.78E-09	0.6792	8.52E-01	7.41E-01	0.2968	3.05E-01	0.6581	7.05E-01	4.03E-08
15	58683366	rs1532085	intronic	LIPC, ALDH1A2, RPL28P4	0.1369	9.61E-01	0.7193	1.32E-01	1.77E-10	0.4153	6.20E-01	0.5251	2.09E-01	1.49E-09
16	56985139	rs9989419	regulatory reg	HERPUD1,CETP	0.4489	5.83E-01	0.8875	9.64E-02	2.21E-09	0.5066	5.87E-01	0.9386	2.36E-01	1.86E-08
16	56993324	rs3764261	upstream	HERPUD1, CETP, AC012181	0.7867	2.13E-01	0.5447	2.74E-01	6.97E-29	0.5155	1.55E-01	0.9677	7.76E-02	5.87E-28
16	57005479	rs1532624	intronic	HERPUD1, CETP, SLC12A3, NLRC5,	0.1196	3.99E-01	0.719	1.25E-01	2.97E-22	0.08552	2.53E-01	0.8547	1.90E-02	2.50E-21
16	57006590	rs7499892	intronic	CETP	0.05331	3.72E-01	0.2021	5.81E-02	2.29E-16	0.6125	8.72E-01	0.7906	6.59E-01	1.93E-15

* Information obtained from Ensembl.org

Table S12: Genome-wide significant genes identified by GATES on

a) the univariate sum score of five metabolic syndrome risk factors and
b) the individual five dichotomous metabolic risk factors

Color coding p-values:		
	red	<.000001
	yellow	.000001-.00001
	green	.00001-.0001
	blue	.0001-.001
	black font	.001-.05
	grey font	>.05

Symbol	GATES PValue	IsSignificant	Chromosome	Start_Position	Length	SNP	Position	GeneFeature	IsGATESKeySNP	P

Results Gates on the sum of metabolic syndrome risk factors

Sum of endorsed metabolic syndrome risk factors (see description individual factors below)

SUM 5 metabolic syndrome criteria

No hits

Results Gates on the dichotomous risk factors underlying the sum score

Cut off criteria for metabolic syndrom risk factors were obtained from this website http://www.ncbi.nlm.nih.gov/pmc/articles/PMC1880831/

For TG, HDL, GLU and blood pressure, we used the ATP III criteria for Clinical Identification of the Metabolic Syndrome

For BMI, we used the AACE Clinical Criteria for Diagnosis of the Insulin Resistance Syndrome

For a full description, see Online Methods.

BMI-dichotomous

No hits

TG-dichotomous

GCKR	1.31E-06	true	2	27719705	26846	rs1260326	27730940	exonic	Y	4.02E-07
-	-	-	-	-	-	rs780094	27741237	intronic	N	1.32E-06
-	-	-	-	-	-	rs1260333	27748624	downstream	N	9.56E-04
-	-	-	-	-	-	rs780092	27743154	intronic	N	0.004631
-	-	-	-	-	-	rs780090	27718474	upstream	N	0.09013

HDL-dichotomous

CETP	1.78E-11	true	16	56995834	21923	rs3764261	56993324	upstream	Y	4.03E-12
-	-	-	-	-	-	rs1532624	57005479	intronic	N	2.35E-07
-	-	-	-	-	-	rs7499892	57006590	intronic	N	2.70E-07
-	-	-	-	-	-	rs4784744	57011185	intronic	N	0.01433
-	-	-	-	-	-	rs5882	57016092	exonic	N	0.04149

GLU-dichotomous

No hits

BLOOD PRESSURE - dichotomous

DTX3	5.70E-07	true	12	57998404	5184	rs812315	57993490	intronic	Y	5.71E-07
PIP4K2C	5.70E-07	true	12	57984941	12271	rs812315	57993490	intronic	Y	5.71E-07
KIAA0895	9.95E-07	true	7	36363758	43121	rs197328	36396358	intronic	Y	3.68E-07
-	-	-	-	-	-	rs197324	36387944	intronic	N	6.78E-07
-	-	-	-	-	-	rs6976779	36427461	intronic	N	1.54E-05
-	-	-	-	-	-	rs3779230	36374799	intronic	N	0.198

x

Table S13: Genome-wide significant genes identified by GATES on the 9 continuous metabolic phenotypes separately

Color coding p-values:		
	red	<.000001
	yellow	.000001-.00001
	green	.00001-.0001
	blue	.0001-.001
	black font	.001-.05
	grey font	>.05

Symbol	GATES Gene- p-value	IsSignificant	Chromosome	Start_Position	Length	SNP	Position	GeneFeature	IsGATESKeySNP	SNP p-value
BMI	-									-
TFAP2B	2.14E-06	true	6	50786438	28889	rs987237	50803050	intronic	Y	7.37E-07
	-									-
CRP	-									-
CRP	5.51E-22	true	1	159682078	2302	rs2794520	159678816	downstream	Y	2.92E-22
-	-	-	-	-	-	rs2808630	159680868	downstream	N	1.59E-01
HNF1A-AS1	2.85E-09	true	12	121407640	2456	rs7953249	121403724	downstream	Y	1.44E-09
-	-	-	-	-	-	rs2254779	121409242	ncRNA	N	1.34E-01
HNF1A	6.37E-09	true	12	121416548	23767	rs1169300	121431225	intronic	Y	2.01E-09
-	-	-	-	-	-	rs2464196	121435427	exonic	N	4.78E-09
-	-	-	-	-	-	rs1169302	121432302	intronic	N	1.20E-07
-	-	-	-	-	-	rs735396	121438844	intronic	N	2.13E-07
-	-	-	-	-	-	rs1169307	121438382	intronic	N	2.76E-05
C12orf43	1.48E-08	true	12	121440224	14082	rs2464196	121435427	exonic	Y	4.78E-09
-	-	-	-	-	-	rs735396	121438844	intronic	N	2.13E-07
-	-	-	-	-	-	rs1169307	121438382	intronic	N	2.76E-05
-	-	-	-	-	-	rs12819210	121458400	exonic	N	9.32E-02
OASL	1.70E-06	true	12	121458094	18952	rs10774580	121476423	intronic	Y	4.97E-07
-	-	-	-	-	-	rs7965349	121471931	intronic	N	9.29E-04
-	-	-	-	-	-	rs2859394	121479265	upstream	N	6.54E-02
-	-	-	-	-	-	rs12819210	121458400	exonic	N	9.32E-02
LEPR	1.19E-05	true	1	65991371	109741	rs1892534	66105944	downstream	Y	4.77E-07
-	-	-	-	-	-	rs2025805	65949878	intronic	N	1.50E-03

-	-	-	-	-	-		rs4655537	66058801	intronic	N	3.23E-03
-	-	-	-	-	-		rs12409877	65943872	intronic	N	3.60E-03
-	-	-	-	-	-		rs6588147	65935494	intronic	N	0.004121
-	-	-	-	-	-		rs1137100	66036441	exonic	N	0.005778
-	-	-	-	-	-		rs10493380	66046117	intronic	N	0.009921
-	-	-	-	-	-		rs1782754	65993348	intronic	N	0.01166
-	-	-	-	-	-		rs1022981	66000034	intronic	N	0.01186
-	-	-	-	-	-		rs1171269	65996802	intronic	N	0.012
-	-	-	-	-	-		rs3828034	66062325	intronic	N	0.01439
-	-	-	-	-	-		rs3790426	66043019	intronic	N	0.135
-	-	-	-	-	-		rs6673324	66031063	intronic	N	0.1814
-	-	-	-	-	-		rs9436297	65888854	intronic	N	0.2146
-	-	-	-	-	-		rs3806318	65885357	upstream	Y	0.2579
-	-	-	-	-	-		rs1137101	66058513	exonic	N	0.2595
-	-	-	-	-	-		rs2154380	66057441	intronic	N	0.272
-	-	-	-	-	-		rs1171279	65988493	intronic	N	0.46
-	-	-	-	-	-		rs3790433	65894342	intronic	N	0.6308
-	-	-	-	-	-		rs970467	65906762	intronic	N	7.65E-01
-	-	-	-	-	-		rs10158579	65950056	intronic	N	0.7728
-	-	-	-	-	-		rs1171278	65988146	intronic	N	0.9624
HDL	-										-
CETP	3.07E-28	true		16	56995834	21923	rs3764261	56993324	upstream	Y	6.97E-29
-	-	-	-	-	-		rs1532624	57005479	intronic	N	2.97E-22
-	-	-	-	-	-		rs7499892	57006590	intronic	N	2.29E-16
-	-	-	-	-	-		rs4784744	57011185	intronic	N	0.001547
-	-	-	-	-	-		rs5882	57016092	exonic	N	0.01234
DPEP3	3.06E-08	true		16	68009565	4888	rs255049	68013471	intronic	Y	3.06E-08
CTCF	3.28E-08	true		16	67596309	76780	rs6499137	67671804	3UTR	Y	2.20E-08
-	-	-	-	-	-		rs7191281	67655133	intronic	N	7.72E-06
NUTF2	4.66E-08	true		16	67880818	24402	rs2271293	67902070	intronic	Y	4.66E-08
NR1H3	7.13E-08	true		11	47279467	11118	rs7120118	47286290	intronic	Y	3.57E-08
-	-	-	-	-	-		rs11039149	47276675	intronic	N	5.09E-04

EDC4	7.13E-08	true	16	67906925	11493	rs2271293	67902070	intronic	Y	4.66E-08
-	-	-	-	-	-	rs10468274	67922342	upstream	N	3.56E-07
C16orf86	8.84E-08	true	16	67700716	1946	rs7187476	67699948	intronic	Y	8.84E-08
ENKD1	8.84E-08	true	16	67696849	3780	rs7187476	67699948	intronic	Y	8.84E-08
FHOD1	9.80E-08	true	16	67263291	18135	rs3852700	67271858	intronic	Y	9.71E-08
-	-	-	-	-	-	rs7196989	67259663	intronic	N	1.51E-07
TMEM208	1.51E-07	true	16	67261015	2168	rs7196989	67259663	intronic	Y	1.51E-07
LRRC29	1.51E-07	true	16	67241041	19681	rs7196989	67259663	intronic	Y	1.51E-07
EXOC3L1	1.64E-07	true	16	67218281	5827	rs868213	67220457	intronic	Y	1.64E-07
PARD6A	1.67E-07	true	16	67694850	1832	rs7187476	67699948	intronic	Y	8.84E-08
-	-	-	-	-	-	rs6979	67691668	exonic	N	0.0283
PRMT7	1.70E-07	true	16	68344876	46294	rs8058517	68379860	intronic	Y	4.37E-08
-	-	-	-	-	-	rs3743742	68345036	intronic	N	0.01252
-	-	-	-	-	-	rs3803650	68344696	exonic	N	0.01556
-	-	-	-	-	-	rs4783554	68384010	intronic	N	0.02467
-	-	-	-	-	-	rs1111571	68363181	intronic	N	0.03644
-	-	-	-	-	-	rs3961282	68365157	intronic	N	0.04415
MADD	1.71E-07	true	11	47291198	60385	rs7120118	47286290	intronic	Y	3.57E-08
-	-	-	-	-	-	rs4752977	47300429	intronic	N	9.72E-06
-	-	-	-	-	-	rs4752979	47339180	intronic	N	1.49E-05
-	-	-	-	-	-	rs749067	47318157	intronic	N	3.01E-05
-	-	-	-	-	-	rs2290149	47345916	intronic	N	0.009796
KIAA0895L	1.72E-07	true	16	67209504	8380	rs868213	67220457	intronic	N	1.64E-07
-	-	-	-	-	-	rs2233455	67207933	exonic	Y	1.72E-07
NOL3	1.72E-07	true	16	67204399	5242	rs2233455	67207933	exonic	Y	1.72E-07
HSF4	1.72E-07	true	16	67197287	6562	rs2233455	67207933	exonic	Y	1.72E-07
PLA2G15	1.95E-07	true	16	68279246	15716	rs1975802	68285847	intronic	Y	1.95E-07
DPEP2	2.36E-07	true	16	68021292	12130	rs255052	68024995	intronic	Y	2.36E-07
NRN1L	3.56E-07	true	16	67918780	1492	rs10468274	67922342	upstream	Y	3.56E-07
PSKH1	6.92E-07	true	16	67927174	36408	rs10468274	67922342	upstream	Y	3.56E-07
CTCFL	9.05E-07	true	20	56093554	6630	rs11700100	56080049	intronic	Y	1.61E-07
NFATC3	1.49E-06	true	16	68119268	143895	rs7193701	68185160	intronic	N	3.87E-07
-	-	-	-	-	-	rs9932251	68131732	intronic	N	7.49E-07

-	-	-	-	-	-	-	rs2418736	68154862	intronic	N	8.20E-07
-	-	-	-	-	-	-	rs4359427	68202747	intronic	Y	8.21E-07
HNF4A	9.90E-06	true		20	42984440	77046	rs1800961	43042364	exonic	Y	7.13E-07
PTPRJ	1.36E-05	true		11	48002109	152158	rs7946766	48004369	intronic	Y	1.35E-06
-	-	-	-	-	-	-	rs3741410	48168664	intronic	N	9.17E-05
-	-	-	-	-	-	-	rs1566729	48164721	intronic	N	1.80E-04
-	-	-	-	-	-	-	rs1113480	48058313	intronic	N	9.83E-04
-	-	-	-	-	-	-	rs6485802	48051442	intronic	N	0.002874
-	-	-	-	-	-	-	rs2270994	48157869	intronic	N	0.008102
-	-	-	-	-	-	-	rs10769317	48150712	intronic	N	0.008523
-	-	-	-	-	-	-	rs4752897	48136990	intronic	N	0.01248
-	-	-	-	-	-	-	rs1875696	48133526	intronic	N	0.01341
-	-	-	-	-	-	-	rs1910364	48113232	intronic	N	0.0152
-	-	-	-	-	-	-	rs12798703	48190777	3UTR	N	0.02903
HAS3	1.80E-05	true		16	69140128	11443	rs2232228	69143577	exonic	Y	7.15E-06
-	-	-	-	-	-	-	rs8047014	69135049	upstream	N	1.32E-04
-	-	-	-	-	-	-	rs3785079	69145476	intronic	N	1.98E-04
VPS4A	2.03E-05	true		16	69345286	13661	rs153050	69349738	intronic	Y	6.09E-06
-	-	-	-	-	-	-	rs246129	69341781	3UTR	N	4.06E-05
APOB	2.16E-05	true		2	21224300	42646	rs673548	21237544	intronic	Y	3.40E-06
-	-	-	-	-	-	-	rs676210	21231524	exonic	N	4.78E-06
-	-	-	-	-	-	-	rs679899	21250914	exonic	N	0.02394
PABPC4	2.65E-05	true		1	40026484	16038	rs4660293	40028180	intronic	Y	2.65E-05
SNORA55	2.65E-05	true		1	40033045	138	rs4660293	40028180	intronic	Y	2.65E-05
SLC9A5	2.86E-05	true		16	67282854	23241	rs7186310	67305513	3UTR	Y	2.86E-05
PLEKHG4	3.43E-05	true		16	67313491	9913	rs11860295	67316234	exonic	Y	1.23E-05
-	-	-	-	-	-	-	rs3868142	67320223	exonic	N	2.17E-05
-	-	-	-	-	-	-	rs8044843	67318242	exonic	N	3.48E-05
KCTD19	4.35E-05	true		16	67323392	37270	rs3868142	67320223	exonic	Y	2.17E-05
-	-	-	-	-	-	-	rs3868143	67328453	intronic	N	2.73E-05
-	-	-	-	-	-	-	rs4783754	67358816	intronic	N	0.01607
LOC284801	5.22E-05	true		20	26167654	22216	rs845785	26194104	upstream	Y	2.65E-05
-	-	-	-	-	-	-	rs12625395	26182690	ncRNA	N	0.02154

DDB2	5.96E-05	true		11	47236492	24278	rs2957873	47249294	intronic	Y	2.04E-05
LRRC36	6.27E-05	true		16	67360746	58364	rs8047080	67402588	intronic	N	3.13E-05
-	-	-	-	-	-		rs11859352	67390448	intronic	Y	3.76E-05
-	-	-	-	-	-		rs4783754	67358816	intronic	N	0.01607
PPIEL	8.29E-05	true		1	39987951	37420	rs4660293	40028180	intronic	Y	2.65E-05
-	-	-	-	-	-		rs3738676	39991588	3UTR	N	0.001231
OR2W3	8.70E-05	true		1	248058888	946	rs6695104	248060894	downstream	Y	3.90E-05
-	-	-	-	-	-		rs11204546	248059712	exonic	N	0.002048
-	-	-	-	-	-		rs10888267	248059423	exonic	N	0.005547
RAPSN	8.99E-05	true		11	47459307	11424	rs7126210	47460306	exonic	Y	8.91E-05
-	-	-	-	-	-		rs3824867	47468569	intronic	N	1.85E-04
	-										-
INS	-										-
No hits	-										-
	-										-
LDL	-										-
PSRC1	4.32E-12	true		1	109822175	3616	rs646776	109818530	downstream	Y	2.19E-12
CELSR2	1.22E-11	true		1	109792640	25739	rs646776	109818530	downstream	Y	2.19E-12
-	-	-	-	-	-		rs611917	109815252	intronic	N	2.49E-07
-	-	-	-	-	-		rs4970833	109804646	intronic	N	1.80E-04
-	-	-	-	-	-		rs585362	109789795	upstream	N	7.88E-04
TOMM40	1.67E-07	true		19	45394476	12471	rs157580	45395266	intronic	Y	4.96E-08
-	-	-	-	-	-		rs2075650	45395619	intronic	N	1.05E-05
PVRL2	3.47E-07	true		19	45349392	32807	rs157580	45395266	intronic	Y	4.96E-08
-	-	-	-	-	-		rs2075650	45395619	intronic	N	1.05E-05
-	-	-	-	-	-		rs6859	45382034	3UTR	N	0.01207
APOB	5.92E-07	true		2	21224300	42646	rs1713222	21271323	upstream	Y	9.33E-08
-	-	-	-	-	-		rs673548	21237544	intronic	N	2.28E-07
-	-	-	-	-	-		rs676210	21231524	exonic	N	2.60E-07
-	-	-	-	-	-		rs1801701	21228827	exonic	N	0.01127
AR	9.83E-07	true	X		66788682	161780	rs5031002	66942625	intronic	Y	2.37E-07
CR1L	1.63E-06	true		1	207818457	78580	rs4844614	207875175	intronic	Y	2.38E-07
-	-	-	-	-	-		rs10494884	207847876	intronic	N	0.004375

-	-	-	-	-	-		rs12080578	207820612	intronic	N	0.005604
-	-	-	-	-	-		rs11118387	207867176	intronic	N	0.006691
-	-	-	-	-	-		rs10779339	207814451	3UTR	N	0.00726
S1PR2	1.68E-06	true		19	10332108	9841	rs10409243	10332988	3UTR	Y	6.23E-07
-	-	-	-	-	-		rs2288937	10340912	intronic	N	0.009269
MIR1908	4.04E-06	true		11	61582632	81	rs174556	61580635	intronic	Y	4.04E-06
FADS1	5.27E-06	true		11	61567096	17434	rs174556	61580635	intronic	Y	4.04E-06
-	-	-	-	-	-		rs174546	61569830	3UTR	N	6.38E-06
FAM222B	8.13E-06	true		17	27082995	86863	rs9303621	27161138	intronic	Y	2.84E-06
-	-	-	-	-	-		rs4795457	27087929	intronic	N	3.28E-06
-	-	-	-	-	-		rs2043031	27085564	exonic	N	3.65E-06
-	-	-	-	-	-		rs11658900	27095485	intronic	N	4.54E-04
LDLR	1.41E-05	true		19	11200037	44469	rs2228671	11210912	exonic	Y	2.34E-06
IGSF5	1.48E-05	true		21	41117333	56691	rs2837175	41131945	intronic	Y	9.66E-07
-	-	-	-	-	-		rs2837227	41172166	intronic	Y	8.46E-05
-	-	-	-	-	-		rs2837226	41167005	intronic	N	1.04E-04
-	-	-	-	-	-		rs2837225	41165462	exonic	N	2.14E-04
-	-	-	-	-	-		rs2837220	41160938	intronic	N	4.84E-04
-	-	-	-	-	-		rs2837205	41146524	intronic	N	5.90E-04
-	-	-	-	-	-		rs2837210	41148235	intronic	N	6.88E-04
-	-	-	-	-	-		rs2837156	41126687	intronic	N	0.001538
-	-	-	-	-	-		rs741867	41134055	intronic	N	0.04886
FEN1	1.90E-05	true		11	61560108	4607	rs102275	61557803	intronic	Y	6.51E-06
MIR611	1.90E-05	true		11	61559966	68	rs102275	61557803	intronic	Y	6.51E-06
DNM2	1.94E-05	true		19	10828728	113859	rs11881315	10909953	intronic	Y	3.05E-06
-	-	-	-	-	-		rs3760781	10947522	upstream	N	2.90E-04
TMEM258	1.95E-05	true		11	61556601	3485	rs102275	61557803	intronic	Y	6.51E-06
FADS2	3.13E-05	true		11	61583674	51153	rs174556	61580635	intronic	Y	4.04E-06
-	-	-	-	-	-		rs2072114	61605215	intronic	N	9.58E-05
-	-	-	-	-	-		rs174570	61597212	intronic	N	5.24E-04
-	-	-	-	-	-		rs2851682	61616012	intronic	N	8.62E-04
-	-	-	-	-	-		rs174611	61627881	intronic	N	0.01518
HMGCR	4.11E-05	true		5	74632992	24935	rs3761740	74632133	upstream	Y	1.44E-05

-	-	-	-	-	-		rs3846662	74651084	intronic	N	1.11E-04
MYRF	4.26E-05	true		11	61522860	33130	rs102275	61557803	intronic	Y	6.51E-06
-	-	-	-	-	-		rs579383	61536583	intronic	N	3.52E-04
-	-	-	-	-	-		rs509360	61548559	intronic	N	0.03373
-	-	-	-	-	-		rs198475	61526071	intronic	N	0.0428
TDRG1	4.26E-05	true		6	40346162	1470	rs4145199	40351841	downstream	Y	9.06E-06
	-										-
SBP	-										-
SMEK2	6.58E-06	true		2	55774427	70434	rs782602	55849309	upstream	Y	8.02E-07
-	-	-	-	-	-		rs782586	55836165	intronic	N	1.20E-06
-	-	-	-	-	-		rs782588	55841640	intronic	N	1.23E-06
-	-	-	-	-	-		rs10496050	55806313	intronic	N	1.28E-05
-	-	-	-	-	-		rs2291336	55845351	upstream	N	1.21E-04
-	-	-	-	-	-		rs12993624	55793476	intronic	N	3.08E-04
-	-	-	-	-	-		rs13010217	55806462	intronic	N	5.40E-04
-	-	-	-	-	-		rs6737995	55819752	intronic	N	1.00E-03
PCDHA12	1.39E-05	true		5	140254886	2589	rs251373	140252615	intronic	Y	2.32E-06
-	-	-	-	-	-		rs3756325	140368859	intronic	N	5.02E-04
-	-	-	-	-	-		rs56003	140298532	intronic	N	6.17E-04
-	-	-	-	-	-		rs155818	140302162	intronic	N	8.10E-04
-	-	-	-	-	-		rs31874	140369318	intronic	N	9.46E-04
PCDHA8	1.42E-05	true		5	140220906	171024	rs251373	140252615	intronic	Y	2.32E-06
-	-	-	-	-	-		rs251370	140246287	intronic	N	2.84E-06
-	-	-	-	-	-		rs3756325	140368859	intronic	N	5.02E-04
-	-	-	-	-	-		rs56003	140298532	intronic	N	6.17E-04
-	-	-	-	-	-		rs155818	140302162	intronic	N	8.10E-04
-	-	-	-	-	-		rs31874	140369318	intronic	N	9.46E-04
PCDHA9	1.42E-05	true		5	140227356	164574	rs251373	140252615	intronic	Y	2.32E-06
-	-	-	-	-	-		rs251370	140246287	intronic	N	2.84E-06
-	-	-	-	-	-		rs3756325	140368859	intronic	N	5.02E-04
-	-	-	-	-	-		rs56003	140298532	intronic	N	6.17E-04
-	-	-	-	-	-		rs155818	140302162	intronic	N	8.10E-04
-	-	-	-	-	-		rs31874	140369318	intronic	N	9.46E-04

PCDHA10	1.42E-05	true		5	140235467	156463	rs251373	140252615	intronic	Y	2.32E-06
-	-	-	-	-	-		rs251370	140246287	intronic	N	2.84E-06
-	-	-	-	-	-		rs3756325	140368859	intronic	N	5.02E-04
-	-	-	-	-	-		rs56003	140298532	intronic	N	6.17E-04
-	-	-	-	-	-		rs155818	140302162	intronic	N	8.10E-04
-	-	-	-	-	-		rs31874	140369318	intronic	N	9.46E-04
PCDHA11	1.42E-05	true		5	140247097	4089	rs251373	140252615	intronic	Y	2.32E-06
-	-	-	-	-	-		rs251370	140246287	intronic	N	2.84E-06
-	-	-	-	-	-		rs3756325	140368859	intronic	N	5.02E-04
-	-	-	-	-	-		rs56003	140298532	intronic	N	6.17E-04
-	-	-	-	-	-		rs155818	140302162	intronic	N	8.10E-04
-	-	-	-	-	-		rs31874	140369318	intronic	N	9.46E-04
PCDHA6	1.59E-05	true		5	140207506	2583	rs251373	140252615	intronic	Y	2.32E-06
-	-	-	-	-	-		rs251370	140246287	intronic	N	2.84E-06
-	-	-	-	-	-		rs10038174	140210616	intronic	N	2.64E-04
-	-	-	-	-	-		rs3756325	140368859	intronic	N	5.02E-04
-	-	-	-	-	-		rs56003	140298532	intronic	N	6.17E-04
-	-	-	-	-	-		rs155818	140302162	intronic	N	8.10E-04
-	-	-	-	-	-		rs31874	140369318	intronic	N	9.46E-04
PCDHA7	1.59E-05	true		5	140213968	2371	rs251373	140252615	intronic	Y	2.32E-06
-	-	-	-	-	-		rs251370	140246287	intronic	N	2.84E-06
-	-	-	-	-	-		rs10038174	140210616	intronic	N	2.64E-04
-	-	-	-	-	-		rs3756325	140368859	intronic	N	5.02E-04
-	-	-	-	-	-		rs56003	140298532	intronic	N	6.17E-04
-	-	-	-	-	-		rs155818	140302162	intronic	N	8.10E-04
-	-	-	-	-	-		rs31874	140369318	intronic	N	9.46E-04
PCDHA5	1.67E-05	true		5	140201360	190570	rs251373	140252615	intronic	Y	2.32E-06
-	-	-	-	-	-		rs251370	140246287	intronic	N	2.84E-06
-	-	-	-	-	-		rs10038174	140210616	intronic	N	2.64E-04
-	-	-	-	-	-		rs7732179	140199039	intronic	N	4.25E-04
-	-	-	-	-	-		rs3756325	140368859	intronic	N	5.02E-04
-	-	-	-	-	-		rs56003	140298532	intronic	N	6.17E-04
-	-	-	-	-	-		rs155818	140302162	intronic	N	8.10E-04

-	-	-	-	-	-	rs31874	140369318	intronic	N	9.46E-04
PCDHA4	1.68E-05	true	5	140186658	205272	rs251373	140252615	intronic	Y	2.32E-06
-	-	-	-	-	-	rs251370	140246287	intronic	N	2.84E-06
-	-	-	-	-	-	rs10038174	140210616	intronic	N	2.64E-04
-	-	-	-	-	-	rs1030166	140185473	intronic	N	2.69E-04
-	-	-	-	-	-	rs3822346	140187322	exonic	N	3.23E-04
-	-	-	-	-	-	rs7732179	140199039	intronic	N	4.25E-04
-	-	-	-	-	-	rs3756325	140368859	intronic	N	5.02E-04
-	-	-	-	-	-	rs56003	140298532	intronic	N	6.17E-04
-	-	-	-	-	-	rs155818	140302162	intronic	N	8.10E-04
-	-	-	-	-	-	rs31874	140369318	intronic	N	9.46E-04
PCDHA1	1.68E-05	true	5	140165720	226210	rs251373	140252615	intronic	Y	2.32E-06
-	-	-	-	-	-	rs251370	140246287	intronic	N	2.84E-06
-	-	-	-	-	-	rs10038174	140210616	intronic	N	2.64E-04
-	-	-	-	-	-	rs1030166	140185473	intronic	N	2.69E-04
-	-	-	-	-	-	rs3822346	140187322	exonic	N	3.23E-04
-	-	-	-	-	-	rs3756338	140180561	intronic	N	3.52E-04
-	-	-	-	-	-	rs7732179	140199039	intronic	N	4.25E-04
-	-	-	-	-	-	rs3756325	140368859	intronic	N	5.02E-04
-	-	-	-	-	-	rs56003	140298532	intronic	N	6.17E-04
-	-	-	-	-	-	rs155818	140302162	intronic	N	8.10E-04
-	-	-	-	-	-	rs31874	140369318	intronic	N	9.46E-04
PCDHA3	1.68E-05	true	5	140180782	2476	rs251373	140252615	intronic	Y	2.32E-06
-	-	-	-	-	-	rs251370	140246287	intronic	N	2.84E-06
-	-	-	-	-	-	rs10038174	140210616	intronic	N	2.64E-04
-	-	-	-	-	-	rs1030166	140185473	intronic	N	2.69E-04
-	-	-	-	-	-	rs3822346	140187322	exonic	N	3.23E-04
-	-	-	-	-	-	rs3756338	140180561	intronic	N	3.52E-04
-	-	-	-	-	-	rs7732179	140199039	intronic	N	4.25E-04
-	-	-	-	-	-	rs3756325	140368859	intronic	N	5.02E-04
-	-	-	-	-	-	rs56003	140298532	intronic	N	6.17E-04
-	-	-	-	-	-	rs155818	140302162	intronic	N	8.10E-04
-	-	-	-	-	-	rs31874	140369318	intronic	N	9.46E-04

PCDHA2	1.68E-05	true		5	140174443	2620	rs251373	140252615	intronic	Y	2.32E-06
-	-	-	-	-	-		rs251370	140246287	intronic	N	2.84E-06
-	-	-	-	-	-		rs10038174	140210616	intronic	N	2.64E-04
-	-	-	-	-	-		rs1030166	140185473	intronic	N	2.69E-04
-	-	-	-	-	-		rs3822346	140187322	exonic	N	3.23E-04
-	-	-	-	-	-		rs3756338	140180561	intronic	N	3.52E-04
-	-	-	-	-	-		rs7732179	140199039	intronic	N	4.25E-04
-	-	-	-	-	-		rs3756325	140368859	intronic	N	5.02E-04
-	-	-	-	-	-		rs56003	140298532	intronic	N	6.17E-04
-	-	-	-	-	-		rs155818	140302162	intronic	N	8.10E-04
-	-	-	-	-	-		rs31874	140369318	intronic	N	9.46E-04
PNPT1	3.22E-05	true		2	55861197	59849	rs782606	55886602	intronic	Y	5.82E-06
-	-	-	-	-	-		rs2586954	55892261	intronic	N	1.40E-05
-	-	-	-	-	-		rs782637	55894247	intronic	N	2.68E-05
-	-	-	-	-	-		rs2627775	55877113	intronic	N	1.91E-04
-	-	-	-	-	-		rs1822513	55903016	intronic	N	0.01665
	-										-
TG	-										-
GCKR	1.11E-09	true		2	27719705	26846	rs1260326	27730940	exonic	Y	3.56E-10
-	-	-	-	-	-		rs780090	27718474	upstream	N	0.001157
-	-	-	-	-	-		rs780092	27743154	intronic	N	0.002774
APOB	1.28E-07	true		2	21224300	42646	rs673548	21237544	intronic	Y	2.01E-08
-	-	-	-	-	-		rs676210	21231524	exonic	N	3.36E-08

Table S14: Comparison results MGAS, TATES, and GATES when all analyses are conducted on the original 9 continuous metabolic phenotypes

(Method specific results in Supplemental Tables S9, S11, and S13)

Overlapping MGAS/univariate GATES
APOB
AR
C16orf86
CELSR2
CETP
CR1L
CTCF
CTCFL
DNM2
DPEP2
DPEP3
EDC4
ENKD1
EXOC3L1
FADS1
FAM222B
FEN1
FHOD1
HAS3
HNF4A
HSF4
IGSF5
KIAA0895L
LDLR
LEPR
LRRC29
MADD
MIR1908
MIR611

Overlapping MGAS/TATES/Univariate GATES
CRP
C12orf43
CETP
G6PC2
GCKR
HNF1A
HNF1A-AS1
OASL

Unique multivariate Gates
ASB18
ATP13A5
GLT8D1
GNL3
PTPRJ
SNORD19B
SNORD69
TBL2
ZNF101

Unique univariate Gates
DDB2
FADS2
HMGCR
KCTD19
LOC284801
LRRC36
MYRF
OR2W3
PABPC4
PNPT1
PPIEL
PTPRJ
RAPSN
SLC9A5
SNORA55
TDRG1

Unique TATES
HLA-DRB / HLA-DQB1
MDFIC
LIPC / ALDH1A2 / RPL28P4

NFATC3
NOL3
NR1H3
NRN1L
NUTF2
PARD6A
PCDHA1
PCDHA10
PCDHA11
PCDHA12
PCDHA2
PCDHA3
PCDHA4
PCDHA5
PCDHA6
PCDHA7
PCDHA8
PCDHA9
PLA2G15
PPP1R2P3
PRMT7
PSKH1
PSRC1
PVRL2
S1PR2
SMEK2
TFAP2B
TMEM208
TMEM258
TOMM40
VPS4A
